# Nutrient Intake Adequacy among Adults in Indonesia and Malaysia: A Systematic Review and Meta-Analysis

**DOI:** 10.1016/j.cdnut.2025.106010

**Published:** 2025-03-24

**Authors:** Rina Agustina, Rachmi Mufida, Wanda Lasepa, Ajeng Mustika, Ardini Debilauralita, Sepriani T Limbong, Deviana AS Siregar, Erfi Prafiantini, Nurul RM Manikam, Pradana Soewondo

**Affiliations:** 1 Department of Nutrition, Faculty of Medicine, Universitas Indonesia - Dr. Cipto Mangunkusumo General Hospital, Jakarta, Indonesia; 2 Human Nutrition Research Center, Indonesian Medical Education and Research Institute, Faculty of Medicine, Universitas Indonesia, Jakarta, Indonesia; 3 Division of Metabolism and Endocrinology, Department of Internal Medicine, Faculty of Medicine, Universitas Indonesia, Dr. Cipto Mangunkusumo General Hospital, Jakarta, Indonesia

**Keywords:** adults, Indonesia, Malaysia, nutrient adequacy, nutrient intake

## Abstract

Under- or overconsumption of nutrients significantly increases the likelihood of future health impairments. However, comprehensive studies that systematically analyzed nutrient intake, especially among adults, are limited. This systematic review and meta-analysis explored studies reporting macro- and micronutrient intake among adults aged ≥18 y in Indonesia and Malaysia (PROSPERO: CRD42023464054). In total, 4501 studies were retrieved from 4 databases (PubMed, Scopus, ProQuest, and Cochrane) and searched manually from January 1980 to December 2023. Nutrient adequacy was determined by calculating the percentage of Recommended Daily Allowance (RDA)/Recommended Nutrient Intake (RNI), Estimated Average Recommendation (EAR), and Standardized Mean Differences (SMDs) for several nutrients. The systematic review of 82 studies revealed variations in energy and macronutrient intake among Indonesian and Malaysian adults. The meta-analysis showed that protein intake among Malaysians exceeded the recommendation [SMD: 0.56; 95% CI: 0.29, 0.684] but was insufficient among Indonesians (SMD: −0.86 (95% CI: −2.11, 0.39). Twenty-six studies reported insufficient fiber intake (10.7%–72.7% RDA/RNI) in both countries. Fat-soluble vitamin intake, except for vitamin A, was lower than 100% EAR. Meanwhile, a wide range of water-soluble vitamin intake was observed (13%–838% EAR). Calcium intake was reported as insufficient in 18 studies (*N* = 5394) (Overall SMD: −3.69; 95% CI: −4.18, −3.19; Indonesia SMD: −5.55; Malaysia SMD: −3.35). Magnesium intake was inadequate, although phosphorus and sodium intake were excessive in Malaysian adults. Moreover, there was inadequate intake (<100% EAR) of potassium, manganese, and copper among adults in both countries, and also iron and zinc in Indonesia. Selenium intake exceeded the recommendation (33–103 μg/158%–450% EAR) for Indonesians but not for Malaysian adults. In conclusion, Indonesian and Malaysian adults had a wide range of adequacy in energy and nutrient intake particularly for macronutrients and water-soluble vitamins. Some deficiencies in nutrients include fiber, fat-soluble vitamins, calcium, potassium, manganese, and copper persisted in both countries, Indonesians lacked iron and zinc intake, while Malaysians had low magnesium intake. In contrast, excessive sodium and phosphorus intake were observed in Malaysians, while Indonesians showed excessive selenium intake. Multistakeholder collaboration is essential to promote a healthy diet while maintaining regulations for individual dietary intake.

## Introduction

The adult population in the world accounts for 5.16 billion and has been increasing in recent years. Over 10% of the 250 million population are adults aged 19 to 50 y. Sixteen million of the adult population aged <70 suffer premature death caused by noncommunicable diseases (NCDs). Most of the NCD deaths (82%) occur in low- and middle-income countries (LMICs) and have become the number one cause of death in Southeast Asian countries [1,2]. Cardiovascular diseases (CVDs) account for the highest number of deaths of NCDs annually (17.5 million people), followed by cancers (8.2 million), respiratory diseases (4 million), and diabetes (1.5 million) [3]. According to the global burden of diseases, prevalent cases of total CVD nearly doubled in 3 decades, from 271 million in 1990 to 523 million in 2019, remaining the leading cause of disease burden in the world [4]. According to the Health Survey Indonesia 2023, the prevalence of overweight and obesity among adults in Indonesia, as measured by body mass index (BMI), is 37.8%. In comparison, Malaysia has a higher prevalence of 54.4%, according to the National Health and Morbidity Survey 2023 [5,6].

The majority of risk factors are caused by modifiable factors such as high systolic blood pressure, elevated fasting plasma glucose, high levels of low-density lipoprotein cholesterol, obesity meaured by high BMI, impaired kidney function, ambient and household air pollution, low physical activity, dietary intake (under or overconsumed), excessive alcohol consumption and exposure to tobacco smoke [4]. There is an expected sharp increase in health impairments related to dietary risks such as overconsumption of processed meats, excessive sodium intake and trans fats intake, and high-sugar foods and beverages in LMICs in the future [7]. Proper dietary intake is crucial in preventing and reducing the burden of NCDs. However, a shift from a diet based on minimally processed staple foods to consuming fruits, vegetables, and fiber remains below the optimal levels. Furthermore, understanding the dietary risk factors is challenging due to the difficulty of accurately assessing and quantifying the exposure and separating the effects from the other essential covariates.

Indonesia and Malaysia stand out as diverse countries in Southeast Asia with rich culinary traditions and dietary habits influenced by cultural, economic, and environmental factors [8]. Both countries have similarities in cultures, eating habits, and genetic susceptibility to obesity [9]. Indonesia and Malaysia also share common dietary patterns, primarily characterized by the consumption of rice as a staple food accompanied by animal protein and vegetables [10]. Shahar et al. [11] examined the macronutrient intake among Malaysian adults aged 19 to 59 y and found that both protein and fat intake exceeded the Malaysian Recommended Nutrient Intake (RNI) guidelines. However, there is limited evidence of a systematic analysis of macro- and micronutrient intake, particularly among adults.

This thorough review aimed to provide an overview of nutrient intake among adults in Indonesia and Malaysia. The findings can indicate how well Indonesian and Malaysian adults adhere to their respective dietary guidelines. They can also be the basis of recommendations for clinical practice, public health policies, and future research.

## Methods

### Search strategy

We performed a systematic review and meta-analysis to identify studies reporting nutrient (macronutrient and micronutrient) intake. Four databases (PubMed, Scopus, ProQuest, and Cochrane) were searched for relevant studies published from January 1980 to December 2023. The search terms used in this review were Indonesia, Malaysia, adults, young adults, male, female, nutrition intake, consumption, macronutrient, energy, calorie, protein, fat, carbohydrate, micronutrient, and dietary assessment methods.

After screening the studies found in the databases, a manual search was conducted. In addition, the authors' findings in related articles were not restricted to this study. The entire search terms and strategy from each database are reported in Supplementary Table 1. Preferred Reporting Items for Systematic Review and Meta-Analysis (PRISMA) were used as a guideline for the study. The protocol was submitted to the International Prospective Register of Systematic Reviews (PROSPERO) database (registration number CRD42023464054).

### Eligibility criteria

This review included studies that assessed the quantity of dietary intake based on several measurements in units of: kilocalories (kcal) for energy, grams (g) for macronutrients, milligrams (mg) or micrograms (μg) for micronutrients. These studies employed various nutritional assessment methods to quantify nutrient intake including 24-h food recall, food record, and semiquantitative food frequency questionnaire (SQ-FFQ). All studies were conducted among adults aged ≥18 y. We included randomized controlled trials, prospective cohort, case-control, and observational studies in this review. However, we excluded abstracts, unpublished studies or practice guidelines, animal studies, in vitro studies, thesis or dissertation, secondary research, and other review articles. The baseline data were utilized for studies that provided >1 dataset on nutrient intake. This review excluded nonhuman subjects, articles not published in English or Indonesian, and topic deemed irrelevant.

### Data collection and extraction

**FIGURE 1:**
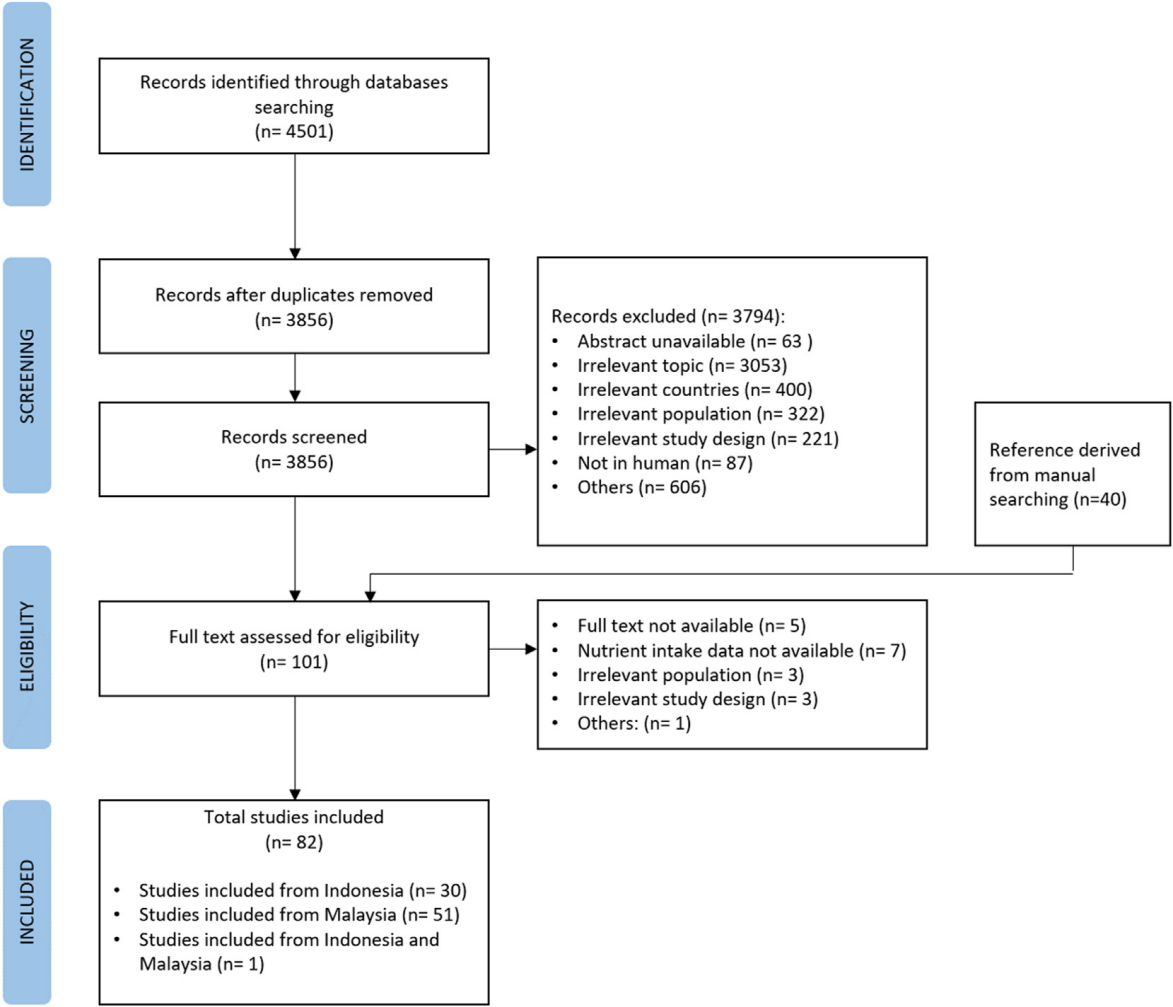
Preferred Reporting Items for Systematic Review and Meta-Analysis (PRISMA) flow chart of the study selection process.

Studies identified through database searches were imported to Rayyan software, where each study was first screened for duplicate. The title and abstract were screened by 4 reviewers (AM, AD, DAS, RM) according to the exclusion and inclusion criteria. The full texts of the selected articles were then further assessed for their eligibility. Disagreements on study selection were resolved through discussions among the reviewers. We extracted data from the included studies, focusing on characteristics of the study population, sample size, dietary assessment methods, nutrient of interest, and nutrient intake references. This study employed various dietary references since each country has its nutritional intake standard tailored to the specific characteristics of its population. The RNI is the national standard for planning and evaluating individual dietary nutrient intake in Malaysia. Indonesia uses the Recommended Dietary Allowance (RDA), which outlines individuals’ mean daily nutrient requirements [12,13]. The Estimated Average Requirement (EAR) represents the amount of a nutrient necessary to meet the estimated nutritional needs of 50% of healthy individuals. The EAR serves as a basis for establishing the RDA. It was chosen as an alternative due to the absence of standardized nutrient values for micronutrients in the guidelines of either country. This approach is grounded in scientific evidence and functions as an international reference. The flow diagram illustrating the studies that follow the PRISMA guidelines is provided in Figure 1.

### Data analysis (meta-analysis)

Selected nutrients included in the meta-analysis were protein, fat, thiamine, pyridoxine, calcium, zinc, iron, and sodium. We analyzed studies that reported continuous data using means and SDs to calculate the standardized mean difference (SMD) for effect size. This approach allowed us to combine all the studies into a single measurement. Studies that did not present data with means and SDs were excluded from the analysis to avoid bias in the results. The meta-analysis was conducted using the RevMan Web, and a random effects model was applied to account for the heterogeneity among the studies, such as differences in sex, culture, sample size, and methods. Understanding this variation is essential for interpreting the findings of the review. Moreover, we presented the result with a 95% confidence interval (CI) for the SMD. The findings were illustrated with each country's mean and RNI/RDA, as shown in the forest plot.

### Risk of bias assessment

Risk of bias appraisal was adapted from Shahar et al. [11] in 2018, and designed exclusively for nutrition research focusing on dietary assessment. The following criteria were used to assess bias in the included studies: Selection bias was evaluated based on the sampling technique, with a score of 1 assigned for random sampling, while a score 2 were given for purposive or convenience sampling. Additionally, studies earned score of 1 if the sample represented adults of all ages and a score of 2 if it focused on specific groups only. Performance bias was assessed based on the dietary assessment method, with the following scores assigned: 1 for food weighing, 2 for multiple 24-h recalls, 3 for food diaries over multiple days, 4 for diet history, 5 for a single 24-h recall, and 6 for food frequency questionnaires (FFQs) measuring usual consumption. Studies also received a score of 1 if they measured usual intake and a score of 2 if they did not. The reporting bias was assessed using 2 questions: whether the study excluded over- or underreporters (score 1 if excluded, 2 if included) and whether all available databases were used (score 1 if all databases were used, 2 if only free databases were used). The overall risk of bias was classified based on the total scores as follows low risk of bias for scores between 6 and 9, moderate risk of scores between 10 and 13, and high risk of scores between 14 and 16. The detailed scoring system and its interpretation can be found in Supplementary Table 2.

## Results

### Summary of study

In total, 4501 articles were retrieved through database screening. Six hundred forty-five articles were removed for duplication. Following this, 3856 articles were screened for the title and abstract review, resulting in the exclusion of 3794 articles (Figure 1). After that, 40 articles were retrieved through manual searched, and 101 articles were assessed for full-text screening and eligibility. Eventually, 82 studies were identified and included in the systematic review. Among the included articles, 30 studies were carried out in Indonesia, 51 were from Malaysia, and a study was conducted in Indonesia and Malaysia. The included studies mainly evaluated both macronutrient (Table 1) and micronutrient intake (Table 2). Food records were the most frequently used dietary assessment methods, followed by food recall and SQ-FFQ. This study identified a low-to-high risk of bias among studies. The complete bias assessment score can be seen in Supplementary Table 2.

**TABLE 1:** Energy and macronutrient intake among adults in Indonesia and Malaysia.

Author, year	Country	Age, y	Sex	Characteristics	Dietary assessment method	Dietary intake	%RNI/RDA
**1. Carbohydrate**							
Wibowo et al. [14], 2015	Indonesia	18–40	F	Women reproductive age	24-h recall	170.6 g	51.2
Yulia et al. [15], 2016	Indonesia	19–49	F	Women reproductive age obese and normal weight	24-h recall	237.6–263.9 g	67.9–75.4
Luglio et al. [16], 2017	Indonesia	21–56	M/F	Healthy adults; urban population	SQ-FFQ	331.8 g	92.0
Sari et al. [17], 2018	Indonesia	18–60	M/F	Tuberculosis patients	2 × 24-h food recall	162.3 g	45.0
Muhammad et al. [18], 2019	Indonesia	41.6 ± 10.2 (19–56)	M/F	Healthy adults; urban population	SQ-FFQ	416.9 g	121.3
Gusnedi et al. [19], 2019	Indonesia	20–44	F	Women reproductive age	2-d 24-h recall; 5-d food record	316.0 g	90.3
Daya et al. [20], 2019	Indonesia	19–59 (31 and 33)	M/F	Obese group and non-obese group	2 × 24-h food recall	167.9–184.7 g	44.5–48.9
Muhammad et al. [18], 2019	Indonesia	19–56 (41.6 ± 10.9)	M/F	Healthy adults; urban population	SQ-FFQ	410.7 g	108.8
Kartiko Sari et al. [21], 2020	Indonesia	55 ± 3.2; 55.8 ± 3.2	F	Menopausal women; Control and Intervention group; Baseline	3-d 24-h recall	258.0–260.0 g	92.1–92.9
Gusnedi et al. [22], 2020	Indonesia	39.5 (21–44); 35.5 (21–44)	F	Food-Based Recommendation and Non-Food-Based Recommendation (Baseline)	SQ-FFQ	258.0–295.0 g	73.8–84.3
Nuryani et al. [23], 2021	Indonesia	16–45	M/F	Adults with normal weight; Adults with central obesity	SQ-FFQ	234.2–235.4 g	62.6–62.9
Alathari et al. [24], 2021	Indonesia	43.8 ± 7.8; 40 ± 10.9; 34.3 ± 10.3	M/F	Vitamin D sufficiency; Vitamin D insufficiency; Vitamin D deficiency	SQ-FFQ	225.9–246.0 g	59.8–65.2
Syauqy et al. [25], 2021	Indonesia	54.8 ± 9.6	M/F	Healthy adults	9-d food recall	264.0 g	85.2
Sari et al. [26], 2021	Indonesia	20–60	M/F	Healthy adults	2-d food recall	41.1 g	11.4
Sari et al. [27], 2021	Indonesia	41.32 ± 10.68	M/F	Healthy adults not consuming vitamin D supplementation (near rubber and oil palm plantations)	2-d food recall	39.2 g	10.4
Yen et al. [28], 2022	Indonesia	18–55	M/F	A T2DM working at telecommunication company-Intervention Group and Adults with T2DM working at the same company-Control Group; Baseline	24-h recall	237.0–240.0 g	66.2–67.0
Sakai et al. [29], 2022	Indonesia	19–29	M	University Students	5-d of observation questionnaire	186.5–245.5 g	54.9–57.1
Khusun et al. [30], 2023	Indonesia	18–88	M/F	Adults in urban and rural population	24-h recall	215.0 g	60.0
Zahra et al. [31], 2023	Indonesia	39.5 ± 5.1	M	Offshore and onshore workers at the oil and gas worksite	1-d weighed food record; 24-h dietary recall; 5-d food tally	247.3–313.4 g	59.6–75.5
Chee et al. [32], 1996	Malaysia	37–39 (18–58)	M	Healthy Malay and Indian adults	3-d weighed food record	251.0–307.0 g	105.5–112.0
Shimbo et al. [33], 1996	Malaysia	18–47	F	Healthy women reproductive age	24-h food recall	280.4–287.5 g	78.8–93.1
Gan et al. [34], 2011	Malaysia	19.98–20.62	M/F	University students	2-d 24-h dietary recall	214.0–279.8 g	93.0–99.9
Karupaiah et al. [35], 2013	Malaysia	19–65	F	Women living in high-rise dwellings	3-d diet record	241.0–260.0 g	109.0–113.0
Md Yusop et al. [36], 2013	Malaysia	49.7±14.1	M/F	HD patients	24-h food recall; 1-d food record	213.4 g	83.4
Hejazi et al. [37], 2014	Malaysia	≥18	M/F	HIV patients with and without hypertension	24-h recall	169.2–170.7 g	67.8–68.4
Menon et al. [38], 2014	Malaysia	20–50	M/F	Newly diagnosed cancer patients	24-h recall	193.0 g	75.5
Chan et al. [39], 2015	Malaysia	63.3 ± 10.5	M/F	Diabetes patients with and without NAFLD	FFQ	165.0–171.0 g	74.7–77.4
Yang et al. [40], 2016	Malaysia	39.3 (38.6; 40.0); 41.5 (40.4; 42.7)	M/F	Healthy adults; Urban population	2 × 24-h food recall	201.5–264.1 g	84.7–96.4
Juliana et al. [41], 2017	Malaysia	40–60	F	Obese; urban population; Control and Intervention; Baseline	DHQ	92.8–97.6 g	40.4–42.5
Karupaiah et al. [42], 2019	Malaysia	38.3 ± 11.4 (20–65)	M/F	Mixed-racial population of healthy free-living adults; Urban population	3-d self-recorded food records; 24-h recall	246.6 g	98.8
Alaini et al. [43], 2019	Malaysia	≥19	M/F	University students and staff; Healthy adults	3-d food record	203.0 g	81.4
Lee & Wan Muda [44], 2019	Malaysia	20–65	M/F	Healthy adults; Urban population	3-d 24-h food recall	200.2 g	80.3
Mitra et al. [45], 2019	Malaysia	18–60 (43.1 and 44.8)	M/F	Overweight/obese adults; Control and Intervention; Baseline	FFQ	251.9–255.6 g	98.4–99.8
Koo et al. [46], 2019	Malaysia	18–29	M/F	Healthy adults; Urban population	3-d 24-h food recall	212.7–235.4 g	64.7–71.1
Chai et al. [47], 2019	Malaysia	30 to >50	F	Adult vegetarians	3-d 24-h food recall	279.4 g	121.7
Chong & Appanah [48], 2019	Malaysia	19–59	F	Women reproductive age; Healthy adults; Rural population	2 × 24-h food recall	174.80–185.50 g	73.45–80.65
Mohd Noh et al. [49], 2020	Malaysia	22.24 ± 1.67; 21.13 ± 1.64	M/F	Athlete in university	FFQ; 3-d food record	386.3–464.8 g	166.0–168.0
Ali et al. [50], 2020	Malaysia	20 to >60 (53 ± 12)	M/F	Multiethnic Asian Dialysis Population	HD-FFQ; 3-d 24-h recall	231.0 g	76.0
Law et al. [51], 2020	Malaysia	20–49	F	Women reproductive age; Healthy adults; Rural population	2 × 24-h food recall	278.8 g	119.2
Hasbullah et al. [52], 2021	Malaysia	21.7	M/F	Undergraduate Student with a family history of T2DM; Undergraduate Student without a family history of T2DM	SQ-FFQ	314.8–321.0 g	123.5–125.9
Shahar et al. [53], 2021	Malaysia	56.3 (6.5)	M/F	Validated method	3-d food recall	167.9 g	65.6
Mazri et al. [54], 2021	Malaysia	40.3 ± 6.9	M/F	Metabolic Healthy Obesity (MHO); Metabolic Unhealthy Obesity (MUO)	7-d DHQ	222.3–231.7 g	86.8–90.5
Zainordin et al. [55], 2021	Malaysia	55 (13); 57.5 (10)	M/F	Diabetic kidney disease patients (Intervention); Diabetic kidney disease patients (Control); Baseline	3-d food diary	124.0–126.5 g	48.5–49.4
Chang et al. [56], 2021	Malaysia	29 ± 7	M/F	Healthy Malaysian adults-Yellowstripe Scad Group; Healthy Malaysian adults-Salmon Group (Baseline)	2-d 24-h food recall	58.6–58.8 g	23.0–23.1
Ong et al. [57], 2022	Malaysia	18–59	M/F	Adults at risk of T2DM (Placebo); Adults at risk of T2DM (Intervention)	24-h recall	224.2–237.4 g	87.8–89.9
Mognard et al. [58], 2023	Malaysia	18–60 and above	M/F	Urban and rural population	24-h recall	204.7 g	80.1
Ghazali & Isa [59], 2023	Malaysia	22.8 ± 0.9	M/F	Validated method in young adults in university	3-d food record	151.0 g	59.2
Amsah et al. [60], 2023	Malaysia	52.30 (6.70)	M/F	T2DM patients	3-d 24-h dietary recall data	281.5 g	110.0
Ching et al. [61], 2023	Malaysia	50±5	M/F	Vegetarian - Chinese Group and Vegetarian - Indian Group	FFQ	452.0–581.0 g	176.6–227.0
**2. Energy**							
Pakasi et al. [62], 2009	Indonesia	28 (22–39)	M/F	Tuberculosis Patients	24-h recall; SQ-FFQ	922.4 kcal	34.8
Wibowo et al. [14], 2015	Indonesia	18–40	F	Women reproductive age	24-h recall	1089 kcal	50.2
Fabiana, 2016 [63]	Indonesia	14–50	F	Women reproductive age	24-h recall	1147 kcal	55.5
Yulia, et al. [15], 2016	Indonesia	19–49	F	Women reproductive age obese and normal weight	24-h recall	1794–2016 kcal	81.5–91.7
Luglio et al. [16], 2017	Indonesia	21–56	M/F	Healthy adults; Urban population	SQ-FFQ	2044 kcal	90.5
Sari et al. [17], 2018	Indonesia	18–60	M/F	Tuberculosis patients	2 × 24-h food recall	1113 kcal	49.3
Muhammad et al. [18], 2019	Indonesia	41.6 ± 10.2 (19–56)	M/F	Healthy adults; Urban population	SQ-FFQ	590.3 kcal	119.8
Gusnedi et al. [19], 2019	Indonesia	20–44	F	Women reproductive age; Healthy adults; Rural population	2-d 24-h recall; 5-d food record	1858 kcal	86.4
Daya et al. [20], 2019	Indonesia	19–59 (31 and 33)	M/F	Obese group and non-obese group	2 × 24-h food recall	1599–1604 kcal	68.1–68.3
Muhammad et al. [18], 2019	Indonesia	19–56 (41.6 ± 10.9)	M/F	Healthy adults; Urban population	SQ-FFQ	2562 kcal	109.0
Kartiko Sari et al. [21], 2020	Indonesia	55 ± 3.2; 55.8 ± 3.2	F	Menopausal women; Control and Intervention group; Baseline	3-d 24-h recall	1943–2055 kcal	108.0–114.2
Alsulami et al. [64], 2020	Indonesia	25–60 (37.08 ± 11.68 and 42.58 ± 8.62)	F	Minangkabau Women (Non-centrally obese WC ≤80 cm and Centrally obese WC >80 cm)	SQ-FFQ	1756–1790 kcal	81.7–83.2
Gusnedi et al. [22], 2020	Indonesia	39.5 (21–44); 35.5 (21–44)	F	Food-Based Recommendation and Non-Food-Based Recommendation (Baseline)	SQ-FFQ	1816–1918 kcal	82.6–87.2
Weta et al. [65], 2020	Indonesia	20.9 ± 1.8 (18–25); 20.6 ± 1.5 (18–25)	F	Obese Young Women (Intervention); Obese Young Women (Control); Baseline	FFQ; SQ-FFQ	1803–1812 kcal	80.1–80.5
Nuryani et al. [23], 2021	Indonesia	16–45	M/F	Adults with normal weight; Adults with central Obesity	SQ-FFQ	1990–2005 kcal	83.2–83.8
Alathari et al. [24], 2021	Indonesia	43.8 ± 7.8; 40 ± 10.9; 34.3 ± 10.3	M/F	Vitamin D sufficiency; Vitamin D insufficiency; Vitamin D deficiency	SQ-FFQ	1695–1894 kcal	72.1–80.6
Syauqy et al. [25], 2021	Indonesia	54.8 ± 9.6	M/F	Validated method	9-d food recall	1751 kcal	88.7
Sari et al. [26], 2021	Indonesia	20–60	M/F	Healthy adults	2-d food recall	1255 kcal	55.6
Muhammad et al. [66], 2021	Indonesia	20–56	M/F	Healthy Adults - Baseline	SQ-FFQ	2570 kcal	113.8
Sari et al. [27], 2021	Indonesia	41.32 ± 10.68	M/F	Healthy adults not consuming vitamin D supplementation (near rubber and oil palm plantations)	2-d food recall	1105 kcal	47.0
Widhani et al. [67], 2022	Indonesia	18–60	M/F	SLE Patients (Synbiotic Group); SLE Patients (Control Group); Baseline	FFQ	1319–1352 kcal	58.0–59.5
Yen et al. [28], 2022	Indonesia	18–55	M/F	Adults with T2DM working at telecommunication company-Intervention Group and Adults with T2DM working at the same company-Control Group; Baseline	24-h recall	1543–1598 kcal	67.9–70.3
Budiyati et al. [68], 2022	Indonesia	29.1 ± 4.3; 29 ± 3.4	M	T2DM Patients Offspring (Non-First Degree Relative); T2DM Patients Offspring (First Degree Relative); Baseline	3-d 24-h dietary recall data	1,484–1,580 kcal	56.0–59.6
Sakai et al. [29], 2022	Indonesia	19–29	M	University Students	5-d of observation questionnaire	1393–1693 kcal	61.9–63.9
Khusun et al. [30], 2023	Indonesia	18–88	M/F	Adults in Urban and rural population	24-h recall	1626 kcal	71.6
Zahra et al. [31], 2023	Indonesia	39.5 ± 5.1	M	Offshore and onshore workers at the oil and gas worksite	1-d weighed food record; 24-h dietary recall; 5-d food tally	2067–2438 kcal	81.7–95.6
Chee et al. [32], 1996	Malaysia	37–39 (18–58)	M	Healthy Malay and Indian adults	3-d weighed food record	1538–2032 kcal	82.8–91.1
Shimbo et al. [33], 1996	Malaysia	18–47	F	Healthy women reproductive age	24-h food recall	1843–1961 kcal	97.0–102.7
Ting et al. [69], 2007	Malaysia	59 ± 3	F	Postmenopausal Chinese women intervention and control group	3-d food records	1366–1569 kcal	77.2–82.6
Yong et al. [70], 2009	Malaysia	20–65	M/F	University students and staff	2-d 24-h recall	1641 kcal	82.3
Gan et al. [34], 2011	Malaysia	19.98–20.62	M/F	University students	2-d 24-h dietary recall	1624–2120 kcal	88.3–94.6
Ismail et al. [71], 2012	Malaysia	18–30	M/F	Acne vulgaris patients (case) and control	3-d food diaries	1590–2056 kcal	85.0–92.8
Karupaiah et al. [35], 2013	Malaysia	19–65	F	Women living in high-rise dwellings	3-d diet record	1703–1867 kcal	96.2–109.0
Menon et al. [38], 2014	Malaysia	20–50	M/F	Newly diagnosed cancer patients	24-h recall	1499 kcal	73.8
Chan et al. [39], 2015	Malaysia	63.3 ± 10.5	M/F	Diabetes patients with and without NAFLD	FFQ	1242–1272 kcal	65.4–66.9
Yang et al. [40], 2016	Malaysia	39.3 (38.6; 40.0); 41.5 (40.4; 42.7)	M/F	Healthy adults; Urban population	2 × 24-h food recall	1511–1957 kcal	79.5–89.4
Juliana et al. [41], 2017	Malaysia	40–60	F	Obese; Urban population; Control and Intervention; Baseline	DHQ	1221–1672 kcal	66.5–91.1
Karupaiah et al. [42], 2019	Malaysia	38.3 ± 11.4 (20–65)	M/F	Mixed-racial population of healthy free-living adults; Urban population	3-d self-recorded food records; 24-h recall	1825 kcal	91.5
Alaini et al. [43], 2019	Malaysia	≥19	M/F	University students and staff; Healthy adults	3-d food record	1580 kcal	79.2
Lee & Wan Muda [44], 2019	Malaysia	20–65	M/F	Healthy adults; Urban population	3-d 24-h food recall	1550 kcal	77.7
Mitra et al. [45], 2019	Malaysia	18–60 (43.1 and 44.8)	M/F	Overweight/obese adults; Control and Intervention; Baseline	FFQ	1995–2021 kcal	97.6–98.8
Koo et al. [46], 2019	Malaysia	18–29	M/F	Healthy adults; Urban population	3-d 24-h food recall	1984–2280 kcal	101.8–107.8
Chai et al. [47], 2019	Malaysia	30 to >50	F	Adult vegetarians	3-d 24-h food recall	1780 kcal	93.7
Chong & Appanah [48], 2019	Malaysia	19–59	F	Women reproductive age; Healthy adults; Rural population	2 × 24-h food recall	1303–1370 kcal	68.6–74.5
Mohd Noh et al. [49], 2020	Malaysia	22.24 ± 1.67; 21.13 ± 1.64	M/F	Athlete in University	FFQ; 3-d food record	2628–2865 kcal	127.9–142.8
Mohd Yusof et al. [72], 2020	Malaysia	48 ± 9 and 48 ± 10 (18–65)	M/F	T2DM Patients; Standard group and structured Ramadan nutrition therapy group; Baseline	3-d food record	1429–1472 kcal	69.9–72.0
Khor & Chong [73], 2020	Malaysia	50.3 ± 14.7; 54.5 ± 13.3	M/F	HD patients	2 × 24-h food recall	1403 kcal	71.1
Ali et al. [50], 2020	Malaysia	20 to >60 (53 ± 12)	M/F	Multiethnic Asian Dialysis Population	HD-FFQ; 3-d 24-h recall	1555 kcal	76.0
Law et al. [51], 2020	Malaysia	20–29 and 30–49	F	Women reproductive age; Healthy adults; Rural population	2 × 24-h food recall	1910–1988 kcal	100.5–108.0
Hasbullah et al. [52], 2021	Malaysia	21:07	M/F	Undergraduate Student with a family history of T2DM; Undergraduate Student without a family history of T2DM	SQ-FFQ	2471–2505 kcal	134.3–136.1
Shahar et al. [53], 2021	Malaysia	56.3 (6.5)	M/F	Validated method	3-d food recall	1152 kcal	56.3
Mazri et al. [54], 2021	Malaysia	40.3 ± 6.9	M/F	Metabolic Healthy Obesity (MHO); Metabolic Unhealthy Obesity (MUO)	7-d DHQ	1819–1893 kcal	89.0–92.6
Zainordin et al. [55], 2021	Malaysia	55 (13); 57.5 (10)	M/F	Diabetic kidney disease patients (Intervention); Diabetic kidney disease patients (Control); Baseline	3-d food diary	955.9–965.4 kcal	46.7–47.2
Chang et al. [56], 2021	Malaysia	29 ± 7	M/F	Healthy Malaysian adults-Yellowstripe Scad Group; Healthy Malaysian adults-Salmon Group (Baseline)	2-d 24-h food recall	1356–1359 kcal	66.5–66.6
Ong et al. [57], 2022	Malaysia	18–59	M/F	Adults at risk of T2DM (Placebo); Adults at risk of T2DM (Intervention)	24-h recall	1837–1920 kcal	89.9–94.0
Abu Bakar et al. [74], 2022	Malaysia	51.6 ± 9.1	M/F	Adults having 3 metabolic syndrome risk factors	FFQ	1815 kcal	88.8
Teong et al. [75], 2022	Malaysia	47.5 ± 15.3; 49.15 ± 13.63	M/F	HD treatment patients (Intervention); HD treatment patients (Control); Baseline	3-d dietary recall	1609–1623 kcal	78.7–79.4
Foo et al. [76], 2023	Malaysia	58.2 ± 6.7	M/F	Chinese-origin adults	FFQ	1817–2061 kcal	95.6–101.5
Mognard et al. [58], 2023	Malaysia	18 to ≥60	M/F	Urban and rural population	24-h recall	1808 kcal	88.5
Ghazali & Isa [59], 2023	Malaysia	22.8 ± 0.9	M/F	Validated Method in young adults in university	3-d food record	1286 kcal	63.0
Amsah et al. [60], 2023	Malaysia	52.30 (6.70)	M/F	T2DM patients	3-d 24-h dietary recall data	1611 kcal	78.8
Abd Rashid et al. [77], 2023	Malaysia	61.0 (11.8)	M/F	Patient with colorectal cancer risk (Cases); Patient with colorectal cancer risk (Control); Baseline	FFQ	1881–1946 kcal	99.0–102.4
Ching et al. [61], 2023	Malaysia	50 ± 5	M/F	Vegetarian - Chinese Group and Vegetarian - Indian Group	FFQ	2532–3597 kcal	123.8–175.9
**3. Fat**							
Wibowo et al. [14], 2015	Indonesia	18–40	F	Women reproductive age	24-h recall	28.0 g	43.1
Yulia et al. [15], 2016	Indonesia	19–49	F	Women reproductive age obese and normal weight	24-h recall	70.2–83.1 g	112.3–133.0
Luglio et al. [16], 2017	Indonesia	21–56	M/F	Healthy adults; Urban population	SQ-FFQ	53.5 g	84.5
Sari et al. [17], 2018	Indonesia	18–60	M/F	Tuberculosis patients	2 × 24-h food recall	26.2 g	41.4
Muhammad et al. [18], 2019	Indonesia	41.6 ± 10.2 (19–56)	M/F	Healthy adults; Urban population	SQ-FFQ	66.6 g	111.0
Gusnedi et al. [19], 2019	Indonesia	20–44	F	Women reproductive age; Healthy adults; Rural population	2-d 24-h recall; 5-d food record	40.0 g	83.3
Daya et al. [20], 2019	Indonesia	19–59 (31 and 33)	M/F	Obese group and non-obese group	2 × 24-h food recall	61.0–66.2 g	93.9–101.9
Muhammad et al. [18], 2019	Indonesia	19–56 (41.6 ± 10.9)	M/F	Healthy adults; Urban population	SQ-FFQ	66.7 g	102.6
Kartiko Sari et al. [21], 2020	Indonesia	55 ± 3.2 and 55.8 ± 3.2	F	Menopausal women; Control and Intervention group; Baseline	3-d 24-h recall	83.0–89.0 g	166.0–178.0
Gusnedi et al. [22], 2020	Indonesia	39.5 (21–44); 35.5 (21–44)	F	Food-Based Recommendation; Non-Food-Based Recommendation (Baseline)	SQ-FFQ	53.9–59.6 g	86.2–95.4
Weta et al. [65], 2020	Indonesia	20.9 ± 1.8 (18–25); 20.6 ± 1.5 (18–25)	F	Obese Young Women (Intervention); Obese Young Women (Control); Baseline	FFQ; SQ-FFQ	66.0–78.6 g	101.5–120.9
Nuryani et al. [23], 2021	Indonesia	16–45	M/F	Adults with normal weight; Adults with central Obesity	SQ-FFQ	76.6–81.4 g	78.6–83.5
Alathari et al. [24], 2021	Indonesia	43.8 ± 7.8; 40 ± 10.9; 34.3 ± 10.3	M/F	Vitamin D sufficiency; Vitamin D insufficiency; Vitamin D deficiency	SQ-FFQ	54.7–64.1 g	84.2–98.7
Syauqy et al. [25], 2021	Indonesia	54.8 ± 9.6	M/F	Validated method	9-d food recall	55.0 g	100.0
Sari et al. [26], 2021	Indonesia	20–60	M/F	Healthy adults	2-d food recall	41.1 g	64.9
Sari et al. [27], 2021	Indonesia	41.32 ± 10.68	M/F	Healthy adults not consuming vitamin D supplementation (near rubber and oil palm plantations)	2-d food recall	39.2 g	60.3
Yen et al. [28], 2022	Indonesia	18–55	M/F	Adults with T2DM working at telecommunication company-Intervention Group and Adults with T2DM working at the same company-Control Group; Baseline	24-h recall	38.0–46.0 g	56.8–68.8
Sakai et al. [29], 2022	Indonesia	19–29	M	University students	5-d of observation questionnaire	50.1–51.7 g	68.9–77.1
Khusun et al. [30], 2023	Indonesia	18–88	M/F	Adults in urban and rural population	24-h recall	60.2 g	90.0
Zahra et al. [31], 2023	Indonesia	39.5 ± 5.1	M	Offshore and onshore workers at the oil and gas worksite	1-d weighed food record, 24-h dietary recall, and 5-d food tally	81.6–93.4 g	136.0–155.7
Chee et al. [32], 1996	Malaysia	37–39 (18–58)	M	Healthy Malay and Indian adults	3-d weighed food record	40.0–51.0 g	75.5–83.6
Shimbo et al. [33], 1996	Malaysia	18–47	F	Healthy women reproductive age	24-h food recall	54.7–62.2 g	99.1–109.1
Yong et al. [70], 2009	Malaysia	20–65	M/F	University students and staff	2-d 24-h recall	50.3 g	90.8
Gan et al. [34], 2011	Malaysia	19.98–20.62	M/F	University students	2-d 24-h dietary recall	75.7–110.6 g	122.1–216.9
Karupaiah et al. [35], 2013	Malaysia	19–65	F	Women living in high-rise dwellings	3-d diet record	49.0–53.0 g	94.3–103.9
Md Yusop et al. [36], 2013	Malaysia	49.7 ± 14.1	M/F	HD patients	24-h food recall; 1-d food record	42.8 g	81.5
Hejazi et al. [37], 2014	Malaysia	≥18	M/F	HIV patients with and without hypertension	24-h recall	54.1–55.5 g	97.8–100.3
Menon et al. [38], 2014	Malaysia	20–50	M/F	Newly diagnosed cancer patients	24-h recall	42.0 g	74.0
Chan et al. [39], 2015	Malaysia	63.3 ± 10.5	M/F	Diabetes patients with and without NAFLD	FFQ	37.4 g	71.2
Yang et al. [40], 2016	Malaysia	39.3 (38.6; 40.0); 41.5 (40.4; 42.7)	M/F	Healthy adults; Urban population	2 × 24-h food recall	52.5–67.0 g	99.1–109.8
Juliana et al. [41], 2017	Malaysia	40–60	F	Obese; Urban population; Control and intervention; Baseline	DHQ	57.3–122.0 g	110.2–234.6
Karupaiah et al. [42], 2019	Malaysia	38.3 ± 11.4 (20–65)	M/F	Mixed-racial population of healthy free-living adults; Urban population	3-d self-recorded food records; 24-h recall	64.5 g	116.6
Alaini et al. [43], 2019	Malaysia	≥19	M/F	University students and staff; Healthy adults	3-d food record	48.0 g	86.8
Lee & Wan Muda [44], 2019	Malaysia	20–65	M/F	Healthy adults; Urban population	3-d 24-h food recall	50.8 g	91.8
Mitra et al. [45], 2019	Malaysia	18–60 (43.1 and 44.8)	M/F	Overweight/obese adults; Control and Intervention; Baseline	FFQ	80.8–84.7 g	141.8–148.6
Koo et al. [46], 2019	Malaysia	18–29	M/F	Healthy adults; Urban population	3-d 24-h food recall	54.5–62.1 g	100.2–106.9
Chai et al. [47], 2019	Malaysia	30 to >50	F	Adult vegetarians	3-d 24-h food recall	47.0 g	90.3
Chong & Appanah [48], 2019	Malaysia	19–59	F	Women reproductive age; Healthy adults; Rural population	2 × 24-h food recall	41.7–45.6 g	78.7–89.4
Mohd Noh et al. [49], 2020	Malaysia	22.24 ± 1.67; 21.13 ± 1.64	M/F	Athlete in university	FFQ; 3-d food record	61.8–74.5 g	99.7–146.1
Ali et al. [50], 2020	Malaysia	20 to >60 (53 ± 12)	M/F	Multiethnic Asian Dialysis Population	HD-FFQ; 3-d 24-h recall	47.0 g	82.5
Law et al. [51], 2020	Malaysia	20–49	F	Women reproductive age; Healthy adults; Rural population	2 × 24-h food recall	54.5 g	104.9
Hasbullah et al. [52], 2021	Malaysia	21:07	M/F	Undergraduate Student with a family history of T2DM; Undergraduate Student without a family history of T2DM	SQ-FFQ	85.8–86.0 g	151.9–152.2
Shahar et al. [53], 2021	Malaysia	56.3 (6.5)	M/F	Validated method	3-d food recall	34.9 g	61.2
Mazri et al. [54], 2021	Malaysia	40.3 ± 6.9	M/F	Metabolic Healthy Obesity (MHO); Metabolic Unhealthy Obesity (MUO)	7-d DHQ	72.6–76.0 g	127.4–133.3
Zainordin et al. [55], 2021	Malaysia	55 (13); 57.5 (10)	M/F	Diabetic kidney disease patients (Intervention); Diabetic kidney disease patients (Control); Baseline	3-d food diary	33.0–45.9 g	52.8–73.5
Chang et al. [56], 2021	Malaysia	29 ± 7	M/F	Healthy Malaysian adults - Yellowstripe Scad Group and Healthy Malaysian adults - Salmon Group (Baseline)	2-d 24-h food recall	47.5–47.6 g	84.1–84.3
Ong et al. [57], 2022	Malaysia	18–59	M/F	Adults at risk of T2DM (Placebo); Adults at risk of T2DM (Intervention)	24-h recall	74.9–80.2 g	123.0–131.7
Foo et al. [76], 2023	Malaysia	58.2 ± 6.7	M/F	Chinese-origin adults	FFQ	52.1–53.6 g	80.0–84.2
Mognard et al. [58], 2023	Malaysia	18 to ≥60	M/F	Urban and rural population	24-h recall	77.7 g	127.6
Ghazali & Isa [59], 2023	Malaysia	22.8 ± 0.9	M/F	Validated method in young adults in university	3-d food record	52.9 g	85.0
Amsah et al. [60], 2023	Malaysia	52.30 (6.70)	M/F	T2DM patients	3-d 24-h dietary recall data	35.3 g	56.5
Ching et al. [61], 2023	Malaysia	50 ± 5	M/F	Vegetarian - Chinese Group and Vegetarian - Indian Group	FFQ	60.5–112.0 g	96.8–179.2
**4. Fiber**							
Sari et al. [17], 2018	Indonesia	18–60	M/F	Tuberculosis patients	2 × 24-h food recall	4.8 g	15.2
Muhammad et al. [18], 2019	Indonesia	41.6 ± 10.2 (19–56)	M/F	Healthy adults; Urban population	SQ-FFQ	24.3 g	80.3
Gusnedi et al. [19], 2019	Indonesia	20–44	F	Women reproductive age; Healthy adults; Rural population	2-d 24-h recall and 5-d food record	9.1 g	30.2
Muhammad et al. [18], 2019	Indonesia	19–56 (41.6 ± 10.9)	M/F	Healthy adults; Urban population	SQ-FFQ	24.0 g	72.7
Kartiko Sari et al. [21], 2020	Indonesia	55 ± 3.2; 55.8 ± 3.2	F	Menopausal women; Control and Intervention group; Baseline	3-d 24-h recall	9.5–10.0 g	38.0–40.0
Alsulami et al. [64], 2020	Indonesia	25–60 (37.08 ± 11.68 and 42.58±8.62)	F	Minangkabau Women (Non-centrally obese WC ≤80 cm and Centrally obese WC >80 cm)	SQ-FFQ	8.6–9.1 g	28.5–30.4
Gusnedi et al. [22], 2020	Indonesia	39.5 (21–44); 35.5 (21–44)	F	Food-Based Recommendation; Non-Food-Based Recommendation (Baseline)	SQ-FFQ	11.2–12.3 g	36.1–39.7
Weta et al. [65], 2020	Indonesia	20.9 ± 1.8 (18–25); 20.6 ± 1.5 (18–25)	F	Obese Young Women (Intervention); Obese Young Women (Control); Baseline	FFQ; SQ-FFQ	8.1–11.9 g	26.6–39.0
Nuryani et al. [23], 2021	Indonesia	16–45	M/F	Adults with normal weight; Adults with central Obesity	SQ-FFQ	15.0–15.6 g	44.8–46.5
Alathari et al. [24], 2021	Indonesia	43.8 ± 7.8; 40 ± 10.9; 34.3 ± 10.3	M/F	Vitamin D sufficiency; Vitamin D insufficiency; Vitamin D deficiency	SQ-FFQ	8.3–10.2 g	25.2–30.9
Syauqy et al. [25], 2021	Indonesia	54.8 ± 9.6	M/F	Validated method	9-d food recall	15.0 g	54.6
Sari et al. [26], 2021	Indonesia	20–60	M/F	Healthy adults	2-d food recall	8.9 g	28.1
Widhani et al. [67], 2022	Indonesia	18–60	M/F	SLE Patients (Synbiotic Group); SLE Patients (Control Group); Baseline	FFQ	8.6–9.6 g	26.8–30.0
Sakai et al. [29], 2022	Indonesia	19–29	M	University students	5-d of observation questionnaire	7.2–9.3 g	22.5–25.1
Khusun et al. [30], 2023	Indonesia	18–88	M/F	Adults in urban and rural population	24-h recall	6.2 g	19.0
Zahra et al. [31], 2023	Indonesia	39.5 ± 5.1	M	Offshore and onshore workers at the oil and gas worksite	1-d weighed food record; 24-h dietary recall; 5-d food tally	13.2–15.8 g	40.0–47.9
Shimbo et al. [33], 1996	Malaysia	18–47	F	Healthy women reproductive age	24-h food recall	3.0–4.3 g	10.7–17.2
Ismail et al. [71], 2012	Malaysia	18–30	M/F	Acne vulgaris patients (case) and control	3-d food diaries	5.3–7.6 g	26.5–38.0
Karupaiah et al. [35], 2013	Malaysia	19–65	F	Women living in high-rise dwellings	3-d diet record	17.4–21.4 g	86.8–106.8
Alaini et al. [43], 2019	Malaysia	≥19	M/F	University students and staff; Healthy adults	3-d food record	7.5 g	37.5
Lee & Wan Muda [44], 2019	Malaysia	20–65	M/F	Healthy adults; Urban population	3-d 24-h food recall	3.0 g	15.5
Koo et al. [46], 2019	Malaysia	18–29	M/F	Healthy adults; Urban population	3-d 24-h food recall	2.2 g	11.0
Chai et al. [47], 2019	Malaysia	30 to >50	F	Adult vegetarians	3-d 24-h food recall	10.4 g	52.0
Mohd Noh et al. [49], 2020	Malaysia	22.24 ± 1.67; 21.13 ± 1.64	M/F	Athlete in university	FFQ; 3-d food record	20.2–20.5 g	100.8–102.6
Mohd Yusof et al. [72], 2020	Malaysia	48 ± 9 and 48 ± 10 (18–65)	M/F	T2DM Patients; Standard group and structured Ramadan nutrition therapy group; Baseline	3-d food record	4.0–5.0 g	20.0–25.0
Hasbullah et al. [52], 2021	Malaysia	21:07	M/F	Undergraduate Student with a family history of T2DM; Undergraduate Student without a family history of T2DM	SQ-FFQ	6.2–6.4 g	31.0–32.0
Ong et al. [57], 2022	Malaysia	18–59	M/F	Adults at risk of T2DM (Placebo); Adults at risk of T2DM (Intervention)	24-h recall	6.3–10.9 g	25.2–43.6
Mognard et al. [58], 2023	Malaysia	18 to ≥60	M/F	Urban and rural population	24-h recall	11.0 g	44.0
Ghazali & Isa [59], 2023	Malaysia	22.8 ± 0.9	M/F	Validated method in young adults in university	3-d food record	4.1 g	16.4
Ching et al. [61], 2023	Malaysia	50 ± 5	M/F	Vegetarian - Chinese Group and Vegetarian - Indian Group	FFQ	53.1–61.0 g	212.4–244.0
**5. Monounsaturated fatty acid**							
Muhammad et al. [18], 2019	Indonesia	41.6 ± 10.2 (19–56)	M/F	Healthy adults; Urban population	SQ-FFQ	29.5 g	110.6
Gusnedi et al. [19], 2019	Indonesia	20–44	F	Women reproductive age; Healthy adults; Rural population	2-d 24-h recall; 5-d food record	8.8 g	40.0
Alsulami et al. [64], 2020	Indonesia	25–60 (37.08 ± 11.68 and 42.58±8.62)	F	Minangkabau Women (Non-centrally obese WC ≤80 cm and Centrally obese WC >80 cm)	SQ-FFQ	7.6–9.0 g	30.5–36.0
Gusnedi et al. [22], 2020	Indonesia	39.5 (21–44); 35.5 (21–44)	F	Food-Based Recommendation; Non-Food-Based Recommendation (Baseline)	SQ-FFQ	11.9–15.0 g	54.1–68.2
Syauqy et al. [25], 2021	Indonesia	54.8 ± 9.6	M/F	Validated method	9-d food recall	21.0 g	68.3
Zahra et al. [31], 2023	Indonesia	39.5 ± 5.1	M	Offshore and onshore workers at the oil and gas worksite	1-d weighed food record; 24-h dietary recall; 5-d food tally	28.6–30.0 g	93.0–97.6
Daud et al. [78], 2018	Malaysia	20–62	M/F	Obese adults; Triglyceride normal and elevated triglyceride	24-h recall	8.7–14.0 g	37.9–61.2
Karupaiah et al. [42], 2019	Malaysia	38.3 ± 11.4 (20–65)	M/F	Mixed-racial population of healthy free-living adults; Urban population	3-d self-recorded food records; 24-h recall	25.7 g	96.4
Ching et al. [61], 2023	Malaysia	50 ± 5	M/F	Vegetarian - Chinese Group and Vegetarian - Indian Group	FFQ	16.5–23.4 g	53.7–76.1
**6. ω-3 fatty acids**							
Muhammad et al. [18], 2019	Indonesia	41.6 ± 10.2 (19–56)	M/F	Healthy adults; Urban population	SQ-FFQ	0.5 g	34.1
Gusnedi et al. [19], 2019	Indonesia	20–44	F	Women reproductive age; Healthy adults; Rural population	2-d 24-h recall; 5-d food record	0.9 g	78.2
Gusnedi et al. [22], 2020	Indonesia	39.5( 21–44); 35.5 (21–44)	F	Food-Based Recommendation; Non-Food-Based Recommendation (Baseline)	SQ-FFQ	1.8 g	163.6
Zahra et al. [31], 2023	Indonesia	39.5 ± 5.1	M	Offshore and onshore workers at the oil and gas worksite	FFQ	0.8–0.8 g	50.6–51.9
Foo et al. [76], 2023	Malaysia	58.2 ± 6.7	M/F	Chinese-origin adults	FFQ	1.7–1.7 g	94.4–109.7
**7. ω-6 fatty acids**							
Muhammad et al. [18], 2019	Indonesia	41.6 ± 10.2 (19–56)	M/F	Healthy adults; Urban population	SQ-FFQ	0.9 g	5.9
Gusnedi et al. [19], 2019	Indonesia	20–44	F	Women reproductive age; Healthy adults; Rural population	2-d 24-h recall; 5-d food record	2.1 g	17.3
Gusnedi et al. [22], 2020	Indonesia	39.5 (21–44); 35.5 (21–44)	F	Food-Based Recommendation; Non-Food-Based Recommendation (Baseline)	SQ-FFQ	2.2–2.9 g	18.3–24.2
Zahra et al. [31], 2023	Indonesia	39.5 ± 5.1	M	Offshore and onshore workers at the oil and gas worksite	1-d weighed food record; 24-h dietary recall; 5-d food tally	3.0–5.1 g	17.5–30.1
Foo et al. [76], 2023	Malaysia	58.2 ± 6.7	M/F	Chinese-origin adults	FFQ	3.3–3.4 g	28.0–31.3
**8. Protein**							
Pakasi et al. [62], 2009	Indonesia	28 (22–39)	M/F	Tuberculosis Patients	24-h recall; SQ-FFQ	26.4 g	42.2
Wibowo et al. [14], 2015	Indonesia	18–40	F	Women reproductive age	24-h recall	36.4 g	59.0
Yulia et al. [15], 2016	Indonesia	19–49	F	Women reproductive age obese and normal weight	24-h recall	55.9–59.4 g	93.2–99.0
Luglio et al. [16], 2017	Indonesia	21–56	M/F	Healthy adults; Urban population	SQ-FFQ	60.0 g	96.0
Sari et al. [17], 2018	Indonesia	18–60	M/F	Tuberculosis patients	2 × 24-h food recall	43.2 g	69.1
Muhammad et al. [18], 2019	Indonesia	41.6 ± 10.2 (19–56)	M/F	Healthy adults; Urban population	SQ-FFQ	80.8 g	129.3
Gusnedi et al. [19], 2019	Indonesia	20–44	F	Women reproductive age; Healthy Adults; Rural population	2-d 24-h recall; 5-d food record	55.7 g	97.7
Daya et al. [20], 2019	Indonesia	19–59 (31 and 33)	M/F	Obese group and non-obese group	2 × 24-h food recall	47.1–51.2 g	75.3–82.0
Muhammad et al. [18], 2019	Indonesia	19–56 (41.6 ± 10.9)	M/F	Healthy adults; Urban population	SQ-FFQ	80.0 g	133.3
Kartiko Sari et al. [21], 2020	Indonesia	55 ± 3.2; 55.8 ± 3.2	F	Menopausal women; Control and Intervention group; Baseline	3-d 24-h recall	53.0–57.0 g	88.3–95.0
Gusnedi et al. [22], 2020	Indonesia	39.5 (21–44); 35.5 (21–44)	F	Food-Based Recommendation; Non-Food-Based Recommendation (Baseline)	SQ-FFQ	61.9–65.9 g	103.2–109.8
Nuryani et al. [23], 2021	Indonesia	16–45	M/F	Adults with normal weight; Adults with central Obesity	SQ-FFQ	92.4–97.9 g	142.2–150.7
Alathari et al. [24], 2021	Indonesia	43.8 ± 7.8; 40 ± 10.9; 34.3 ± 10.3	M/F	Vitamin D sufficiency, Vitamin D insufficiency, and Vitamin D deficiency	SQ-FFQ	74.2–82.2 g	118.7–131.5
Syauqy et al. [25],	Indonesia	54.8 ± 9.6	M/F	Validated method	9-d food recall	50.0 g	80.0
Sari et al. [26], 2021	Indonesia	20–60	M/F	Healthy adults	2-d food recall	49.1 g	78.6
Sari et al. [27], 2021	Indonesia	41.32 ± 10.68	M/F	Healthy adults not consuming vitamin D supplementation (near rubber and oil palm plantations)	2-d food recall	44.9 g	71.9
Yen et al. [28], 2022	Indonesia	18–55	M/F	Adults with T2DM working at telecommunication company -Intervention Group and Adults with T2DM working at the same company-Control Group; Baseline	24-h recall	68.0–69.0 g	102.6–104.2
Sakai et al. [29], 2022	Indonesia	19–29	M	University Students	5-d of observation questionnaire	40.9–49.4 g	68.2–76.0
Khusun et al. [30], 2023	Indonesia	18–88	M/F	Adults in Urban and rural population	24-h recall	57.7 g	87.1
Zahra et al. [31], 2023	Indonesia	39.5 ± 5.1	M	Offshore and onshore workers at the oil and gas worksite	1-d weighed food record; 24-h dietary recall; 5-d food tally	84.0–85.5 g	129.2– 131.5
Chee et al. [32], 1996	Malaysia	37–39 (18–58)	M	Healthy Malay and Indian adults	3-d weighed food record	47.0–64.0 g	90.4–104.9
Shimbo et al. [33], 1996	Malaysia	18–47	F	Healthy women reproductive age	24-h food recall	56.7–67.1 g	91.5–110.0
Ting et al. [69], 2007	Malaysia	59 ± 3	F	Postmenopausal Chinese women intervention and control group	3-d food records	57.0–66.0 g	114.0–126.9
Gan et al. [34], 2011	Malaysia	19.98–20.62	M/F	University students	2-d 24-h dietary recall	60.7–80.6 g	114.0–130.0
Karupaiah et al. [35], 2013	Malaysia	19–65	F	Women living in high-rise dwellings	3-d diet record	60.0–71.0 g	120.0–134.0
Hejazi et al. [37], 2014	Malaysia	≥18	M/F	HIV patients with and without hypertension	24-h recall	65.6–68.5 g	117.1–122.3
Menon et al. [38], 2014	Malaysia	20–50	M/F	Newly diagnosed cancer patients	24-h recall	57.0 g	100.0
Chan et al. [39], 2015	Malaysia	63.3 ± 10.5	M/F	Diabetes patients with and without NAFLD	FFQ	52.0–53.1 g	96.3–98.3
Yang et al. [40], 2016	Malaysia	39.3 (38.6; 40.0); 41.5 (40.4; 42.7)	M/F	Healthy adults; Urban population	2 × 24-h food recall	57.8–74.3 g	96.3–114.3
Juliana et al. [41], 2017	Malaysia	40–60	F	Obese; urban population; Control and Intervention; Baseline	DHQ	63.0–129.0 g	105.9–216.8
Karupaiah et al. [42], 2019	Malaysia	38.3 ± 11.4 (20–65)	M/F	Mixed-racial population of healthy free-living adults; urban population	3-d self-recorded food records; 24-h recall	63.5 g	101.6
Alaini et al. [43], 2019	Malaysia	≥19	M/F	University students and staff	3-d food record	56.0 g	100.0
Lee & Wan Muda [44], 2019	Malaysia	20–65	M/F	Healthy adults; Urban population	3-d 24-h food recall	69.3 g	123.8
Mitra et al. [45], 2019	Malaysia	18–60 (43.1 and 44·8)	M/F	Overweight/obese adults; Control and Intervention; Baseline	FFQ	74.1–75.5 g	118.6–120.8
Koo et al. [46], 2019	Malaysia	18–29	M/F	Healthy adults; Urban population	3-d 24-h food recall	57.9–65.8 g	106.1–94.9
Chai et al. [47], 2019	Malaysia	30 to >50	F	Adult vegetarians	3-d 24-h food recall	57.7 g	111.0
Chong & Appanah [48], 2019	Malaysia	19–59	F	Women reproductive age; Healthy adults; Rural population	2 × 24-h food recall	55.5–56.7 g	106.7–107.7
Khor & Chong [73], 2020	Malaysia	50.3 ± 14.7; 54.5 ± 13.3	M/F	HD patients	2 × 24-h food recall	44.9 g	81.3
Ali et al. [50], 2020	Malaysia	20–>60 (53 ± 12)	M/F	Multiethnic Asian Dialysis Population	HD-FFQ; 3-d 24-h recall	53.0 g	101.9
Law et al. [51], 2020	Malaysia	20–49	F	Women reproductive age; Healthy adults; Rural population	2 × 24-h food recall	80.7 g	153.8
Hasbullah et al. [52], 2021	Malaysia	21:07	M/F	Undergraduate Student with a family history of T2DM; Undergraduate Student without a family history of T2DM	SQ-FFQ	92.4–92.9 g	160.7–161.6
Shahar et al. [53], 2021	Malaysia	56.3 (6.5)	M/F	Validated method	3-d food recall	48.4 g	85.7
Mazri et al. [54], 2021	Malaysia	40.3 ± 6.9	M/F	Metabolic Healthy Obesity (MHO); Metabolic Unhealthy Obesity (MUO)	7-d DHQ	69.2–70.6 g	122.5–125.0
Zainordin et al. [55], 2021	Malaysia	55 (13); 57.5 (10)	M/F	Diabetic kidney disease patients (Intervention); Diabetic kidney disease patients (Control); Baseline	3-d food diary	38.1–61.1 g	67.4–108.2
Chang et al. [56], 2021	Malaysia	29 ± 7	M/F	Healthy Malaysian adults-Yellowstripe Scad Group; Healthy Malaysian adults-Salmon Group (Baseline)	2-d 24-h food recall	173.5–173.8 g	301.7–302.3
Ong et al. [57], 2022	Malaysia	18–59	M/F	Adults at risk of T2DM (Placebo); Adults at risk of T2DM (Intervention)	24-h recall	73.5–78.0 g	129.0–136.8
Teong et al. [75], 2022	Malaysia	47.5 ± 15.3; 49.15 ± 13.63	M/F	HD treatment patients (Intervention); HD treatment patients (Control); Baseline	3-d dietary recall	60.2–60.9 g	106.6–107.8
Foo et al. [76], 2023	Malaysia	58.2 ± 6.7	M/F	Chinese-origin adults	FFQ	67.3–74.1 g	121.5–129.4
Mognard et al. [58], 2023	Malaysia	18 to ≥60	M/F	Urban and rural population	24-h recall	74.0 g	129.8
Ghazali & Isa [59], 2023	Malaysia	22.8 ± 0.9	M/F	Validated method in young adults in university	3-d food record	50.1 g	87.1
Amsah et al. [60], 2023	Malaysia	52.30 (6.70)	M/F	T2DM patients	3-d 24-h dietary recall data	52.7 g	93.3
Ching et al. [61], 2023	Malaysia	50±5	M/F	Vegetarian - Chinese Group and Vegetarian - Indian Group	FFQ	69.2–94.2 g	122.5–166.7
**9. Saturated fatty acids**							
Muhammad et al. [18], 2019	Indonesia	41.6 ± 10.2 (19–56)	M/F	Healthy adults; Urban population	SQ-FFQ	25.7 g	114.2
Gusnedi et al. [19], 2019	Indonesia	20–44	F	Women reproductive age; Healthy adults; Rural population	2-d 24-h recall; 5-d food record	21.3 g	89.1
Alsulami et al. [64], 2020	Indonesia	25–60 (37.08 ± 11.68 and 42.58 ± 8.62)	F	Minangkabau Women (Non-centrally obese WC ≤80 cm and Centrally obese WC >80 cm)	SQ-FFQ	20.1–21.8 g	95.6–103.7
Gusnedi et al. [22], 2020	Indonesia	39.5(21–44); 35.5 (21–44)	F	Food-Based Recommendation; Non-Food-Based Recommendation (Baseline)	SQ-FFQ	25.6–26.2 g	111.3–113.9
Khusun et al. [30], 2023	Indonesia	18–88	M/F	Adults in Urban and rural population	24-h recall	23.3 g	105.1
Zahra et al. [31], 2023	Indonesia	39.5±5.1	M	Offshore and onshore workers at the oil and gas worksite	1-d weighed food record; 24-h dietary recall; 5-d food tally	38.9–39.9 g	172.9–177.3
Daud et al. [78], 2018	Malaysia	20–62	M/F	Obese adults; Triglyceride normal and elevated triglyceride	24-h recall	12.9–14.4 g	58.4–65.1
Karupaiah et al. [42], 2019	Malaysia	38.3 ± 11.4 (20–65)	M/F	Mixed-racial population of healthy free-living adults; Urban population	3-d self-recorded food records; 24-h recall	28.7 g	129.5
Alaini et al. [43], 2019	Malaysia	≥19	M/F	University students and staff; Healthy adults	3-d food record	15.0 g	67.7
Chang et al. [56], 2021	Malaysia	29 ± 7	M/F	Healthy Malaysian adults-Yellowstripe Scad Group; Healthy Malaysian adults-Salmon Group (Baseline)	2-d 24-h food recall	13.7–14.8 g	60.7–65.6
Mognard et al. [58], 2023	Malaysia	18 to ≥60	M/F	Urban and rural population	24-h recall	29.5 g	133.1
Ching et al. [61], 2023	Malaysia	50 ± 5	M/F	Vegetarian - Chinese Group and Vegetarian - Indian Group	FFQ	23.4–58.8 g	104.0–261.3
**10. Trans fatty acid**							
Muhammad et al. [18], 2019	Indonesia	41.6 ± 10.2 (19–56)	M/F	Healthy adults; Urban population	SQ-FFQ	2.82 g	141.0

Abbreviations: DHQ, diet history questionnaire; EAR, estimated average recommendation; F, female; FFQ, food frequency questionnaire; HD, hemodialysis; HD-FFQ, hemodialysis food frequency questionnaire; M, male; NAFLD, nonalcoholic fatty liver disease; RDA, recommended daily allowance; RNI, recommended nutrient intake; SLE, systemic lupus erythematosus; SQ-FFQ, semiquantitative food frequency questionnaire; T2DM, type 2 diabetes mellitus; WC, waist circumference.

**TABLE 2:** Micronutrient intake among adults in Indonesia and Malaysia.

First author, year	Country	Age	Sex	Characteristics	Dietary assessment method	Dietary intake	%RNI/RDA	%EAR
**1. Calcium**								
Sari et al. [17], 2018	Indonesia	18–60	M/F	Tuberculosis patients	2 × 24-h food recall	101.3 mg	9.5	11.4
Nuryani et al. [23], 2021	Indonesia	16–45	M/F	Adults with normal weight; Adults with central obesity	SQ-FFQ	437.8–618.1 mg	41.1–58.0	49.3–69.6
Syauqy et al. [25], 2021	Indonesia	54.8 ± 9.6	M/F	Validated method	9-d food recall	477.0 mg	39.8	47.7
Sakai et al. [29], 2022	Indonesia	19–29	M	University students	5-d of observation questionnaire	219.5–221.0 mg	18.3–22.1	22.0–26.5
Khusun et al. [30], 2023	Indonesia	18–88	M/F	Adults in Urban and rural population	24-h recall	505.3 mg	45.9	55.1
Chee et al. [32], 1996	Malaysia	37–39 (18–58)	M/F	Healthy Malay and Indian Adults	3-d weighed food record	198.0–273.0 mg	19.8–27.3	23.8–32.8
Karim & Leong [79], 2000	Malaysia	19–25	M/F	Healthy adults	3-d dietary recall	361.0–440.0 mg	36.1–4	43.3–52.8
Ting et al. [69], 2007	Malaysia	59 ± 3	F	Postmenopausal Chinese women intervention and control group	3-d food records	466.0–477.0 mg	39.8–46.6	47.7–55.9
Mirnalini et al. [80], 2008	Malaysia	18–59	M/F	Adults among 4 zones in Peninsular Malaysia	1-d 24-h recall	397.2 mg	39.72	47.7
Gan et al. [34], 2011	Malaysia	19.98–20.62	M/F	University students	2-d 24-h dietary recall	471.7–547.2 mg	47.2–54.7	56.6–65.7
Karupaiah et al. [35], 2013	Malaysia	19–65	F	Women living in high-rise dwellings	3-d diet record	462.0–511.0 mg	46.2–51.1	55.4–61.3
Md Yusop et al. [36], 2013	Malaysia	49.7 ± 14.1	M/F	HD patients	24-h food recall; 1-d food record	250.0 mg	25.0	30.0
Teng et al. [81], 2013	Malaysia	50–70	M	Healthy men	7-d food intake using DHQ	385.0 mg	38.5	46.2
Menon et al. [38], 2014	Malaysia	20–50	M/F	Newly diagnosed cancer patients	24-h recall	264.0 mg	26.4	31.7
Yang et al. [40], 2016	Malaysia	39.3 (38.6; 40.0); 41.5 (40.4; 42.7)	M/F	Healthy adults; Urban population	2 × 24-h food recall	380.8–400.9 mg	38.1–40.1	45.7–48.1
Kruger et al. [82], 2018	Malaysia	59 (3.9) and 60 (4.3)	F	Postmenopausal Malaysian Chinese women intervention and control; Baseline	3-d diet record; FFQ	527.0–547.0 mg	43.9–45.6	52.7–54.7
Jamil et al. [83], 2018	Malaysia	24.6 (20–50)	M/F	Healthy adults; Period: South-west monsoon, North-east monsoon Year-round	A questionnaire which listed common vitamin D-rich foods and supplement intake	73–83 IU/ 1.83–2.08 μg	12.2–13.9	36.6–41.6
Alaini et al. [43], 2019	Malaysia	≥19	M/F	University students and staff; Healthy adults	3-d food record	418.0 mg	41.8	50.2
Lee & Wan Muda [44], 2019	Malaysia	20–65	M/F	Healthy adults; Urban population	3-d 24-h food recall	490.3 mg	46.4	55.7
Koo et al. [46], 2019	Malaysia	18–29	M/F	Healthy adults; Urban population	3-d 24-h food recall	370.6–412.2 mg	37.1–41.2	44.5–49.5
Chai et al. [47], 2019	Malaysia	30 to >50	F	Adult vegetarians	3-d 24-h food recall	745.3 mg	74.5	89.4
Chong & Appanah [48], 2019	Malaysia	19–59	F	Women reproductive age; Healthy adults; Rural population	2 × 24-h food recall	389.8–489.0 mg	39.0–41.8	46.8–50.1
Ali et al. [50], 2020	Malaysia	20 to >60 (53±12)	M/F	Multiethnic Asian Dialysis Population	HD-FFQ; 3-d 24-h recall	313.0 mg	34.2	34.2
Shahar et al. [53], 2021	Malaysia	56.3 (6.5)	M/F	Validated method	3-d food recall	363.4 g	33.0	39.6
Foo et al. [76], 2023	Malaysia	58.2 ± 6.7	M/F	Chinese-origin adults	FFQ	531.5–562.4 mg	44.3–56.2	53.2–67.5
Mognard et al. [58], 2023	Malaysia	18 to ≥60	M/F	Urban and rural population	24-h recall	418.9 mg	38.1	45.7
Ghazali & Isa [59], 2023	Malaysia	22.8 ± 0.9	M/F	Validated method in young adults in university	3-d food record	347.0 mg	31.6	37.9
Green et al. [84], 2008	Malaysia and Indonesia	18–40	F	Non-pregnant women	1-d 24-h recall	357.0 mg	35.7	42.8
**2. Copper**								
Syauqy et al. [25], 2021	Indonesia	54.8 ± 9.6	M/F	Validated method	9-d food recall	578.0 μg	64.2	64.2
Sakai et al. [29], 2022	Indonesia	19–29	M	University students	5-d of observation questionnaire	0.6–0.8 mg	66.7–88.9	66.7–88.9
Lee & Wan Muda [44], 2019	Malaysia	20–65	M/F	Healthy adults; Urban population	3-d 24-h food recall	0.5 mg	55.6	55.6
**3. Iron**								
Muhammad et al. [18], 2019	Indonesia	41.6 ± 10.2 (19–56)	M/F	Healthy adults; Urban population	SQ-FFQ	18.1 mg	164.6	263.3
Gusnedi et al. [22], 2020	Indonesia	39.5(21–44); 35.5 (21–44)	F	Food-Based Recommendation and Non-Food-Based Recommendation (Baseline)	SQ-FFQ	9.8–10.7 mg	54.4–59.4	59.4–87.1
Nuryani et al. [23], 2021	Indonesia	16–45	M/F	Adults with normal weight; Adults with central obesity	SQ-FFQ	9.3–9.8 mg	70.0–73.6	112.0–117.8
Syauqy et al. [25], 2021	Indonesia	54.8 ± 9.6	M/F	Validated method	9-d food recall	10.2 mg	120.0	120.0
Sakai et al. [29], 2022	Indonesia	19–29	M	University students	5 d of observation questionnaire	3.6–4.7 mg	20.0–52.2	20.0–67.9
Khusun et al. [30], 2023	Indonesia	18–88	M/F	Adults in Urban and rural population	24-h recall	13.2 mg	108.9	174.2
Chee et al. [32], 1996	Malaysia	37–39 (18–58)	M/F	Healthy Malay and Indian Adults	3-d weighed food record	8.6–11.5 mg	29.7–82.1	47.4–106.8
Karim & Leong [79], 2000	Malaysia	19–25	M/F	Healthy adults	3-d dietary recall	14.1–18 mg	48.6–128.6	77.8–167.1
Mirnalini et al. [80], 2008	Malaysia	18–59	M/F	Adults among 4 zones in Peninsular Malaysia	1-d 24-h recall	10.7 mg	59.4	85.2
Gan et al. [34], 2011	Malaysia	19.98–20.62	M/F	University students	2-d 24-h dietary recall	17.2–18.8 mg	86.0–134.3	137.6–174.6
Karupaiah et al. [35], 2013	Malaysia	19–65	F	Women living in high-rise dwellings	3-d diet record	15.0–17.0 mg	51.7–154.5	82.8–247.3
Teng et al. [81], 2013	Malaysia	50–70	M	Healthy men	7-d food intake using DHQ	12.6 mg	90.0	117.0
Menon et al. [38], 2014	Malaysia	20–50	M/F	Newly diagnosed cancer patients	24-h recall	10.8 mg	45.0	72.0
Yang et al. [40], 2016	Malaysia	39.3 (38.6; 40.0); 41.5 (40.4; 42.7)	M/F	Healthy adults; Urban population	2 × 24-h food recall	11.5–15.1 mg	39.7–107.9	39.7–140.2
Alaini et al. [43], 2019	Malaysia	≥19	M/F	University students and staff; Healthy adults	3-d food record	13.0 mg	74.6	119.3
Lee & Wan Muda [44], 2019	Malaysia	20–65	M/F	Healthy adults; Urban population	3-d 24-h food recall	15.8 mg	90.7	145.0
Chai et al. [47], 2019	Malaysia	30 to >50	F	Adult vegetarians	3-d 24-h food recall	19.5 mg	67.2	107.5
Chong & Appanah [48], 2019	Malaysia	19–59	F	Women reproductive age; Healthy adults; Rural population	2 × 24-h food recall	9.8–11.0 mg	33.8–100.0	54.4–160.0
Ali et al. [50], 2020	Malaysia	20 to >60 (53 ± 12)	M/F	Multiethnic Asian Dialysis Population	HD-FFQ; 3-d 24-h recall	11.0 mg	168.2	168.2
Shahar et al. [53], 2021	Malaysia	56.3 (6.5)	M/F	Validated method	3-d food recall	10.2 mg	81.6	106.1
Foo et al. [76], 2023	Malaysia	58.2 ± 6.7	M/F	Chinese-origin adults	FFQ	18.7–20.2 mg	224.4–233.6	291.8–374.0
Mognard et al. [58], 2023	Malaysia	18 to ≥60	M/F	Urban and rural population	24-h recall	15.8 mg	107.9	172.6
Ghazali & Isa [59], 2023	Malaysia	22.8 ± 0.9	M/F	Validated method in young adults in university	3-d food record	8.6 mg	47.8	47.8
**4. Magnesium**								
Muhammad et al. [18], 2019	Indonesia	41.6 ± 10.2 (19–56)	M/F	Healthy adults; Urban population	SQ-FFQ	392.1 mg	112.0	106.0
Nuryani et al. [23], 2021	Indonesia	16–45	M/F	Adults with normal weight; Adults with central obesity	SQ-FFQ	295.7–324.8 mg	98.6–108.3	81.0–89.0
Syauqy et al. [25], 2021	Indonesia	54.8 ± 9.6	M/F	Validated method	9-d food recall	364.0 mg	104.0	98.4
Sakai et al. [29], 2022	Indonesia	19–29	M	University students	5 d of observation questionnaire	110.9–132.5 mg	33.6–36.8	33.1–35.8
Khusun et al. [30], 2023	Indonesia	18–88	M/F	Adults in Urban and rural population	24-h recall	143.4 mg	44.3	38.6
Lee & Wan Muda [44], 2019	Malaysia	20–65	M/F	Healthy adults; Urban population	3-d 24-h food recall	113.7 mg	29.4	31.2
Mognard et al. [58], 2023	Malaysia	18 to ≥60	M/F	Urban and rural population	24-h recall	224.4 mg	58.0	60.4
**5. Manganese**								
Sakai et al. [29], 2022	Indonesia	19–29	M	University students	5 d of observation questionnaire	1.6–2.2 mg	88.9–95.7	88.9–95.7
Lee & Wan Muda [44], 2019	Malaysia	20–65	M/F	Healthy adults; Urban population	3-d 24-h food recall	0.4 mg	19.5	19.5
**6. Phosphorus**								
Nuryani et al. [23], 2021	Indonesia	16–45	M/F	Adults with normal weight; Adults with central obesity	SQ-FFQ	1236–1381 mg	140.0–156.4	213.2–238.2
Syauqy et al. [25], 2021	Indonesia	54.8 ± 9.6	M/F	Validated method	9-d food recall	536.0 mg	76.6	92.4
Sakai et al. [29], 2022	Indonesia	19–29	M	University Students	5 d of observation questionnaire	526.8–618.3 mg	75.3–88.3	90.8–106.6
Khusun et al. [30], 2023	Indonesia	18–88	M/F	Adults in Urban and rural population	24-h recall	800.0 mg	95.5	137.9
Karim & Leong [79], 2000	Malaysia	19–25	M/F	Healthy adults	3-d dietary recall	774.0–1026 mg	110.6–146.6	133.4–176.9
Ting et al. [69], 2007	Malaysia	59 ± 3	F	Postmenopausal Chinese women intervention and control group	3-d food records	644.0–869.0 mg	92.0–124.1	111.0–149.8
Menon et al. [38], 2014	Malaysia	20–50	M/F	Newly diagnosed cancer patients	24-h recall	763.0 mg	109.0	131.6
Lee & Wan Muda [44], 2019	Malaysia	20–65	M/F	Healthy adults; Urban population	3-d 24-h food recall	1049 mg	149.8	180.8
Khor & Chong [73], 2020	Malaysia	50.3 ± 14.7; 54.5 ± 13.3	M/F	HD patients	2 × 24-h food recall	930.0 mg	132.9	160.3
Ali et al. [50], 2020	Malaysia	20 to >60 (53±12)	M/F	Multiethnic Asian Dialysis Population	HD-FFQ; 3-d 24-h recall	643.0 mg	91.9	110.9
Shahar et al. [53], 2021	Malaysia	56.3 (6.5)	M/F	Validated method	3-d food recall	631.0 mg	90.1	90.1
Teong et al. [75], 2022	Malaysia	47.5 ± 15.3; 49.15 ± 13.63	M/F	HD treatment patients (Intervention) and HD treatment patients (Control); Baseline	3-d dietary recall	798.0–817.0 mg	114.0–116.0	137.6–140.9
Ghazali & Isa [59], 2023	Malaysia	22.8 ± 0.9	M/F	Validated method in young adults in university	3-d food record	721.0 mg	103.0	124.3
**7. Potassium**								
Nuryani et al. [23], 2021	Indonesia	16–45	M/F	Adults with normal weight; Adults with central obesity	SQ-FFQ	2343–2561 mg	48.3–52.8	49.9–54.5
Syauqy et al. [25], 2021	Indonesia	54.8 ± 9.6	M/F	Validated method	9-d food recall	3328 mg	70.8	70.8
Sakai et al. [29], 2022	Indonesia	19–29	M	University students	5 d of observation questionnaire	1068–1250 mg	22.7–26.6	22.7–26.6
Khusun et al. [30], 2023	Indonesia	18–88	M/F	Adults in Urban and rural population	24-h recall	1048 mg	21.0	22.3
Zhang et al. [85], 1999	Malaysia	≥18	F	Healthy non-smoking women	1-d 24-h food recall	604.0 mg	12.9	12.9
Karim & Leong [79], 2000	Malaysia	19–25	M/F	Healthy adults	3-d dietary recall	1301–1581 mg	27.7–33.6	27.7–33.6
Md Yusop et al. [36], 2013	Malaysia	49.7 ± 14.1	M/F	HD patients	24-h food recall; 1-d food record	909.0 mg	19.3	19.3
Alaini et al. [43], 2019	Malaysia	≥19	M/F	University students and staff; Healthy adults	3-d food record	1181 mg	25.1	25.1
Lee & Wan Muda [44], 2019	Malaysia	20–65	M/F	Healthy adults; Urban population	3-d 24-h food recall	1307 mg	27.8	27.8
Ali et al. [50], 2020	Malaysia	20 to >60 (53 ± 12)	M/F	Multiethnic Asian Dialysis Population	HD-FFQ; 3-d 24-h recall	1093 mg	23.3	23.3
Shahar et al. [53], 2021	Malaysia	56.3 (6.5)	M/F	Validated method	3-d food recall	1099 mg	23.4	23.4
Mognard et al. [58], 2023	Malaysia	18 to ≥60	M/F	Urban and rural population	24-h recall	2143 mg	45.6	45.6
Ghazali & Isa [59], 2023	Malaysia	22.8 ± 0.9	M/F	Validated method in young adults in university	3-d food record	813.0 mg	17.3	17.3
**8. Selenium**								
Adriani et al. [86], 2015	Indonesia	45–64	F	Postmenopausal women with and without Hypertensive	Food recall 2 × 24-h	33.0–53.0 μg	132.0–212.0	158.4–254.4
Muhammad et al. [18], 2019	Indonesia	41.6 ± 10.2 (19–56)	M/F	Healthy adults; Urban population	SQ-FFQ	103.0 μg	374.6	449.5
Ismail et al. [71], 2012	Malaysia	18–30	M/F	Acne vulgaris patients (case) and control	3-d food diaries	27.7–49.6 μg	86.6–155.0	103.9–186.0
Lee & Wan Muda [44], 2019	Malaysia	20–65	M/F	Healthy adults; Urban population	3-d 24-h food recall	33.6 μg	120.7	144.9
Chong & Appanah [48], 2019	Malaysia	19–59	F	Women reproductive age; Healthy adults; Rural population	2 × 24-h food recall	13.5–15.4 μg	56.3–61.6	67.5–73.9
**9. Sodium**								
Nuryani et al. [23], 2021	Indonesia	16–45	M/F	Adults with normal weight; Adults with central obesity	SQ-FFQ	387.3–426.5 mg	25.3–27.8	25.8–28.4
Syauqy et al. [25], 2021	Indonesia	54.8 ± 9.6	M/F	Validated method	9-d food recall	1580 mg	117.0	105.3
Sakai et al. [29], 2022	Indonesia	19–29	M	University students	5 d of observation questionnaire	1922–2536 mg	128.2–169.1	128.2–169.1
Khusun et al. [30], 2023	Indonesia	18–88	M/F	Adults in urban and rural population	1-d 24-h recall	1368 mg	91.2	91.2
Zahra et al. [31], 2023	Indonesia	39.5 ± 5.1	M	Offshore and onshore workers at the oil and gas worksite	1-d weighed food record; 24-h dietary recall; 5-d food tally	3174–3181 mg	211.6–212.1	211.6–212.1
Zhang et al. [85], 1999	Malaysia	≥18	F	Healthy non-smoking women	24-h food recall	1275 mg	85.0	85.0
Karim & Leong [79], 2000	Malaysia	19–25	M/F	Healthy adults	3-d dietary recall	1807–2670 mg	120.5–178.0	120.5–178.0
Mirnalini et al. [80], 2008	Malaysia	18–59	M/F	Adults among 4 zones in Peninsular Malaysia	1-d 24-h recall	2575 μg	171.7	171.7
Gan et al. [34], 2011	Malaysia	19.98–20.62	M/F	University students	2-d 24-h dietary recall	2322–2971 mg	154.8–198.1	154.8–198.1
Karupaiah et al. [35], 2013	Malaysia	19–65	F	Women living in high-rise dwellings	3-d diet record	2683–2833 mg	178.9–188.9	178.9–188.9
Md Yusop et al. [36], 2013	Malaysia	49.7 ± 14.1	M/F	HD patients	24-h food recall; 1-d food record	1943 mg	129.5	129.5
Alaini et al. [43], 2019	Malaysia	≥19	M/F	University students and staff; Healthy adults	3-d food record	2585 mg	172.3	172.3
Lee & Wan Muda [44], 2019	Malaysia	20–65	M/F	Healthy adults; Urban population	3-d 24-h food recall	1697 mg	113.1	113.1
Ali et al. [50], 2020	Malaysia	20 to >60 (53 ± 12)	M/F	Multiethnic Asian Dialysis Population	HD-FFQ; 3-d 24-h recall	2395 mg	159.7	159.7
Shahar et al. [53], 2021	Malaysia	56.3 (6.5)	M/F	Validated Method	3-d food recall	1619 mg	107.9	107.9
Salleh et al. [87], 2021	Malaysia	48.8 ± 15.63	M/F	Adults in Urban and Rural Malaysia	FFQ	2608 mg	173.9	173.9
Mognard et al. [58], 2023	Malaysia	18 to ≥60	M/F	Urban and rural population	24-h recall	2367 mg	157.8	157.8
Ghazali & Isa [59], 2023	Malaysia	22.8 ± 0.9	M/F	Validated method in young adults in university	3-d food record	1876 mg	125.1	125.1
**10. Vitamin A**								
Pakasi et al. [62], 2009	Indonesia	28 (22–39)	M/F	Tuberculosis patients	24-h recall; SQ-FFQ	282.0 mg	47.0	65.8
Jati et al. [96], 2014	Indonesia	20–52	F	Healthy adults	24-h recall	508.6 μg	84.8	118.7
Muhammad et al. [18], 2019	Indonesia	41.6 ± 10.2 (19–56)	M/F	Healthy adults; Urban population	SQ-FFQ	574.3 RE	91.9	128.6
Nuryani et al. [23], 2021	Indonesia	16–45	M/F	Adults with normal weight; Adults with central obesity	SQ-FFQ	2235.5–2478.8 μg	353.0–391.4	494.2–548.0
Widhani et al. [67], 2022	Indonesia	18–60	M/F	SLE Patients (Synbiotic Group); SLE Patients (Control Group); Baseline	FFQ	705.4–836.0 μg	117.6–139.3	164.6–195.1
Sakai et al. [29], 2022	Indonesia	19–29	M	University student	5 d of observation questionnaire	249.1–269.4 μg	41.5–44.9	58.1–62.9
Khusun et al. [30], 2023	Indonesia	18–88	M/F	Adults in Urban; Rural population	24-h recall	833.5 μg	138.9	194.5
Ismail et al. [88], 1995	Malaysia	20–31	M	Athlete (sepak takraw)	3-d weighed food record	2023 μg (RE)	337.2	472.0
Chee et al. [32], 1996	Malaysia	37–39 (18–58)	M/F	Healthy Malay; Indian Adults	3-d weighed food record	399.0–503.0 μg (RE)	66.5–83.8	93.1–117.4
Moy & Rahman [89], 2002	Malaysia	40–60+	M/F	Diabetes patients	24-h recall	432.0–614.0 mg	72.0–102.3	100.8–114.2
Gan et al. [34], 2011	Malaysia	19.98–20.62	M/F	University students	2-d 24-h dietary recall	803.2–982.1 μg	133.9–163.7	187.4–229.2
Ismail et al. [71], 2012	Malaysia	18–30	M/F	Acne vulgaris patients (case) and control	3-d food diaries	1260–1515 RE	210.0–252.4	293.9–353.4
Karupaiah et al. [35], 2013	Malaysia	19–65	F	Women living in high-rise dwellings	3-d diet record	699.0–972.0 mg	116.5–162.0	163.1–226.8
Pirabbasi et al. [90], 2016	Malaysia	43–76	M	COPD patients; Control and Intervention; Baseline	CNAQ; CNAQ+ Vitamin C + NAC supplement; CNAQ+ Vitamin C supplement; CNAQ + NAC supplement	1104–1353 RE	184.1–225.6	257.7–315.9
Yang et al. [40], 2016	Malaysia	39.3 (38.6; 40.0); 41.5 (40.4; 42.7)	M/F	Healthy adults; Urban population	2 × 24-h food recall	670.3 μg	89.3–111.7	125.0–156.4
Chai et al. [47], 2019	Malaysia	30 to >50	F	Adult vegetarians	3-d 24-h food recall	992.7 RE	165.4	231.6
Chong & Appanah [48], 2019	Malaysia	19–59	F	Women reproductive age; Healthy adults; Rural population	2 × 24-h food recall	531.2–647.0 μg	88.5–107.8	124.0–151.0
Shahar et al. [53], 2021	Malaysia	56.3 (6.5)	M/F	Validated Method	3-d food recall	495.2 μg	82.5	115.6
Mognard et al. [58], 2023	Malaysia	18 to ≥60	M/F	Urban and rural population	24-h recall	456.6 μg	76.1	106.5
**11. Vitamin D**								
Sari et al. [17], 2018	Indonesia	18–60	M/F	Tuberculosis patients	2 × 24-h food recall	4.2 μg	28.0	84.0
Muhammad et al. [18], 2019	Indonesia	41.6 ± 10.2 (19–56)	M/F	Healthy adults; urban population	SQ-FFQ	3.4 μg	22.9	68.8
Syauqy et al. [25], 2021	Indonesia	54.8 ± 9.6	M/F	Validated method	9-d food recall	2.5 μg	16.6	49.8
Sari et al. [26 or 27], 2021	Indonesia	20–60	M/F	Healthy adults	2-d food recall	17.4 mg	116.0	348.0
Sari et al. [22 or 115], 2021	Indonesia	41.32 ± 10.68	M/F	Healthy Adults not consuming vitamin D supplementation (near rubber and oil palm plantations)	2-d food recall	3.6 g	23.9	71.8
Sakai et al. [29], 2022	Indonesia	19–29	M	University students	5 d of observation questionnaire	1.9–2.0 μg	12.7–13.3	38.0–40.0
Khusun et al. [30], 2023	Indonesia	18–88	M/F	Adults in urban and rural population	24-h recall	1.8 μg	12.0	36.0
Jamil et al. [83], 2018	Malaysia	20–55	F	Malay office worker; Healthy adults; Urban population	FFQ	5.2 μg	34.7	104.0
Foo et al. [76], 2023	Malaysia	58.2 ± 6.7	M/F	Chinese-origin adults	FFQ	4.1–5.4 μg	27.3–36.0	82.0–108.0
Mognard et al. [58], 2023	Malaysia	18 to ≥60	M/F	Urban and rural population	24-h recall	1.9 μg	12.7	38.0
**12. Vitamin E**								
Muhammad et al. [18], 2019	Indonesia	41.6 ± 10.2 (19–56)	M/F	Healthy adults; Urban population	SQ-FFQ	2.7 mg	18.0	22.1
Nuryani et al. [23], 2021	Indonesia	16–45	M/F	Adults with normal weight; Adults with central obesity	SQ-FFQ	8.3–8.3 mg	55.3–55.4	66.3–66.5
Syauqy et al. [25], 2021	Indonesia	54.8 ± 9.6	M/F	Validated method	9-d food recall	2.0 mg	13.5	17.6
Widhani et al. [67], 2022	Indonesia	18–60	M/F	SLE Patients (Synbiotic Group); SLE Patients (Control Group); Baseline	FFQ	3.2–3.6 mg	21.4–24.0	27.8–31.2
Sakai et al. [29], 2022	Indonesia	19–29	M	University students	5 d of observation questionnaire	5.2–5.6 mg	34.7–37.3	41.6–48.5
Khusun et al. [30], 2023	Indonesia	18–88	M/F	Adults in Urban and rural population	24-h recall	7.9 mg	52.7	68.5
Ismail et al. [71], 2012	Malaysia	18–30	M/F	Acne vulgaris patients (case) and control	3-d food diaries	4.1–5.9 mg	27.3–59.0	33.5–75.2
Pirabbasi et al. [90], 2016	Malaysia	43–76	M	COPD patient; Control and Intervention; Baseline	CNAQ; CNAQ+ Vitamin C + NAC supplement; CNAQ+ Vitamin C supplement; CNAQ + NAC supplement	2.8–3.6 mg	27.7–35.5	36.0–46.2
Mognard et al. [58], 2023	Malaysia	18 to ≥60	M/F	Urban and rural population	24-h recall	0.9 mg	7.2	9.4
**13. Vitamin K**								
Sakai et al. [29], 2022	Indonesia	19–29	M	University students	5 d of observation questionnaire	79.2–91.6 μg	140.9–144.0	76.3–88.0
**14. Vitamin B1/thiamine**								
Muhammad et al. [18], 2019	Indonesia	41.6 ± 10.2 (19–56)	M/F	Healthy adults; Urban population	SQ-FFQ	1.4 mg	120.9	145.0
Sofyan et al. [91], 2021	Indonesia	28.7 ± 4.25	M/F	Nurses with vitamin B supplementation and Nurses without vitamin B supplementation	SQ-FFQ	0.7–26.7 mg	61.7–2,321	74.1–2,785
Syauqy et al. [25], 2021	Indonesia	28.7 ± 4.25	M/F	Nurses with vitamin B supplementation and Nurses without vitamin B supplementation	9-d food recall	0.7–26.7 mg	61.7–2,321	74.1–2,785
Sakai et al. [29], 2022	Indonesia	19–29	M	University students	5 d of observation questionnaire	0.3–0.4 mg	27.3–33.3	32.7–40.0
Khusun et al. [30], 2023	Indonesia	18–88	M/F	Adults in Urban and rural population	24-h recall	0.7 mg	60.9	73.0
Chee et al. [32], 1996	Malaysia	37–39 (18–58)	M/F	Healthy Malay and Indian Adults	3-d weighed food record	0.5–0.8 mg	45.5–66.7	54.5–80.0
Gan et al. [34], 2011	Malaysia	19.98–20.62	M/F	University students	2-d 24-h dietary recall	0.7–1.0 mg	67.3–80.0	80.7–96.0
Karupaiah et al. [35], 2013	Malaysia	19–65	F	Women living in high-rise dwellings	3-d diet record	0.8–2.1 mg	72.7–190.9	87.3–229.1
Menon et al. [38], 2014	Malaysia	20–50	M/F	Newly diagnosed cancer patients	24-h recall	0.7 mg	60.9	73.
Yang et al. [40], 2016	Malaysia	39.3 (38.6; 40.0); 41.5 (40.4; 42.7)	M/F	Healthy adults; Urban population	2 × 24-h food recall	0.6–0.7 mg	54.6–58.3	65.5–70.0
Koo et al. [46], 2019	Malaysia	18–29	M/F	Healthy adults; Urban population	3-d 24-h food recall	0.7–0.8 mg	58.3–72.7	70.0–87.3
Chong & Appanah [48], 2019	Malaysia	19–59	F	Women reproductive age; Healthy adults; Rural population	2 × 24-h food recall	0.6 mg	54.6	65.5
Mognard et al. [58], 2023	Malaysia	18 to ≥60	M/F	Urban and rural population	24-h recall	1.1 mg	95.7	114.8
Ghazali & Isa [59], 2023	Malaysia	22.8 ± 0.9	M/F	Validated method in young adults in university	3-d food record	0.5 mg	43.5	52.2
**15. Vitamin B2/riboflavin**								
Muhammad et al. [18], 2019	Indonesia	41.6 ± 10.2 (19–56)	M/F	Healthy adults; Urban population	SQ-FFQ	2.0 mg	164.2	197.0
Syauqy et al. [25], 2021	Indonesia	54.8 ± 9.6	M/F	Validated Method	9-d food recall	1.6 mg	136.7	164.0
Sakai et al. [29], 2022	Indonesia	19–29	M	University students	5 d of observation questionnaire	0.5–0.6 mg	45.5–46.2	54.6–55.4
Khusun et al. [30], 2023	Indonesia	18–88	M/F	Adults in Urban and rural population	24-h recall	1.0 mg	83.3	100.0
Ismail et al. [88], 1995	Malaysia	20–31	M	Athlete (sepak takraw)	3-d weighed food record	2.5 mg	192.3	230.8
Chee et al. [32], 1996	Malaysia	37–39 (18–58)	M	Healthy Malay and Indian Adults	3-d weighed food record	0.7–1.0 mg	63.6–76.9	76.4–92.3
Moy & Rahman [89], 2002	Malaysia	40–60+	M/F	Diabetes patients	24-h recall	0.9–1.2 mg	79.1–100.0	94.9–120.0
Gan et al. [34], 2011	Malaysia	19.98–20.62	M/F	University students	2-d 24-h dietary recall	1.0–1.1 mg	87.7–88.2	105.2–105.8
Karupaiah et al. [35], 2013	Malaysia	19–65	F	Women living in high-rise dwellings	3-d diet record	1.2–2.6 mg	109.1–236.4	130.9–283.6
Menon et al. [38], 2014	Malaysia	20–50	M/F	Newly diagnosed cancer patients	24-h recall	0.9 mg	75.0	90.0
Yang et al. [40], 2016	Malaysia	39.3 (38.6; 40.0); 41.5 (40.4; 42.7)	M/F	Healthy adults; Urban population	2 × 24-h food recall	0.8–1.0 mg	72.7–76.9	87.3–92.3
Koo et al. [46], 2019	Malaysia	18–29	M/F	Healthy adults; Urban population	3-d 24-h food recall	1.1–1.2 mg	84.6–92.3	101.5–110.8
Chong & Appanah [48], 2019	Malaysia	19–59	F	Women reproductive age; Healthy adults; Rural population	2 × 24-h food recall	0.8 mg	72.7	87.3
Mognard et al. [58], 2023	Malaysia	18 to ≥60	M/F	Urban and rural population	24-h recall	1.2 mg	100.0	120.0
Ghazali & Isa [59], 2023	Malaysia	22.8 ± 0.9	M/F	Validated method in young adults in university	3-d food record	0.7 mg	58.3	70.0
**16. Vitamin B3/niacin**								
Muhammad et al. [18], 2019	Indonesia	41.6 ± 10.2 (19–56)	M/F	Healthy adults; Urban population	SQ-FFQ	19.6 mg	130.5	169.6
Syauqy et al. [25], 2021	Indonesia	54.8 ± 9.6	M/F	Validated method	9-d food recall	12.7 mg	84.7	110.1
Sakai et al. [29], 2022	Indonesia	19–29	M	University students	5 d of observation questionnaire	6.9–8.1 mg	49.3–50.6	64.1–65.8
Khusun et al. [30], 2023	Indonesia	18–88	M/F	Adults in urban and rural population	24-h recall	13.0 mg	86.7	112.7
Ismail et al. [88], 1995	Malaysia	20–31	M	Athlete (sepak takraw)	3-d weighed food record	19.5 mg	121.9	158.4
Chee et al. [32], 1996	Malaysia	37–39 (18–58)	M/F	Healthy Malay and Indian Adults	3-d weighed food record	5.3–72.0 mg	37.9–450.0	49.2–585.0
Moy & Rahman [89], 2002	Malaysia	40–60+	M/F	Diabetes patients	24-h recall	7.1–10.9 mg	50.7–68.1	65.9–88.6
Gan et al. [34], 2011	Malaysia	19.98–20.62	M/F	University students	2-d 24-h dietary recall	8.5–9.8 mg	60.7–61.3	78.9–79.6
Karupaiah et al. [35], 2013	Malaysia	19–65	F	Women living in high-rise dwellings	3-d diet record	14.0–15.0 mg	100.0–107.1	130.0–139.3
Menon et al. [38], 2014	Malaysia	20–50	M/F	Newly diagnosed cancer patients	24-h recall	9.0 mg	60.0	78.0
Yang et al. [40], 2016	Malaysia	39.3 (38.6; 40.0); 41.5 (40.4; 42.7)	M/F	Healthy adults; Urban population	2 × 24-h food recall	6.4–9.0 mg	45.7–56.3	59.4–73.1
Koo et al. [46], 2019	Malaysia	18–29	M/F	Healthy adults; Urban population	3-d 24-h food recall	10.2–11.7 mg	63.8–73.1	82.9–95.1
Chong & Appanah [48], 2019	Malaysia	19–59	F	Women reproductive age; Healthy adults; Rural population	2 × 24-h food recall	7.7–7.9 mg	55.0–56.4	71.5–73.4
Mognard et al. [58], 2023	Malaysia	18 to ≥60	M/F	Urban and rural population	24-h recall	21.70 mg	144.7	188.1
Ghazali & Isa [59], 2023	Malaysia	22.8 ± 0.9	M/F	Validated method in young adults in university	3-d food record	6.9 mg	46.0	59.8
**17. Vitamin B6**								
Muhammad et al. [18], 2019	Indonesia	41.6 ± 10.2 (19–56)	M/F	Healthy adults; Urban population	SQ-FFQ	4.6 mg	316.6	379.9
Sofyan et al. [91], 2021	Indonesia	28.7 ± 4.25	M/F	Nurses with vitamin B supplementation and Nurses without vitamin B supplementation	SQ-FFQ	1.8–9.1 mg	134.6–698.5	161.5–838.2
Sakai et al. [29], 2022	Indonesia	19–29	M	University students	5 d of observation questionnaire	0.5–0.6 mg	38.5–46.2	46.2–55.4
**18. Vitamin B9/folate**								
Sofyan et al. [91], 2021	Indonesia	28.7 ± 4.25	M/F	Nurses with vitamin B supplementation and Nurses without vitamin B supplementation	SQ-FFQ	228.6–305.0 μg	57.2–76.3	71.5–95.3
Koo et al. [46], 2019	Malaysia	18–29	M/F	Healthy adults; Urban population	3-d 24-h food recall	53.6–59.6 μg	13.4–14.9	16.8–18.6
Chai et al. [47], 2019	Malaysia	30 to >50	F	Adult vegetarians	3-d 24-h food recall	192.9 μg	48.2	60.3
Mognard et al. [58], 2023	Malaysia	18 to ≥60	M/F	Urban and rural population	24-h recall	305.9 μg	76.5	95.6
**19. Vitamin B12**								
Muhammad et al. [18], 2019	Indonesia	41.6 ± 10.2 (19–56)	M/F	Healthy adults; Urban population	SQ-FFQ	7.4 μg	184.5	221.4
Sofyan et al. [91], 2021	Indonesia	28.7 ± 4.25	M/F	Nurses with vitamin B supplementation and Nurses without vitamin B supplementation	SQ-FFQ	6.9–9.3 mg	173.0–233.5	207.6–280.2
Sakai et al. [29], 2022	Indonesia	19–29	M	University students	5 d of observation questionnaire	2.5–3.2 μg	62.5–80.0	75.0–96.0
Chai et al. [47], 2019	Malaysia	30 to >50	F	Adult vegetarians	3-d 24-h food recall	0.5 μg	12.8	15.3
**20. Vitamin C**								
Muhammad et al. [18], 2019	Indonesia	41.6 ± 10.2 (19–56)	M/F	Healthy adults; Urban population	SQ-FFQ	152.3 mg	184.6	233.8
Nuryani et al. [23], 2021	Indonesia	16–45	M/F	Adults with normal weight; Adults with central obesity	SQ-FFQ	61.1–62.7 mg	71.9–73.7	86.3–88.5
Syauqy et al. [25], 2021	Indonesia	54.8 ± 9.6	M/F	Validated method	9-d food recall	90.0 mg	105.9	137.7
Widhani et al. [67], 2022	Indonesia	18–60	M/F	SLE Patients (Synbiotic Group); SLE Patients (Control Group); Baseline	FFQ	37.7–67.1 mg	44.3–78.9	57.6–102.6
Sakai et al. [29], 2022	Indonesia	19–29	M	University students	5 d of observation questionnaire	29.2–37.5 mg	30.7–50.0	36.9–65.0
Khusun et al. [30], 2023	Indonesia	18–88	M/F	Adults in Urban and rural population	24-h recall	12.8 mg	18.3	23.8
Ismail et al. [88], 1995	Malaysia	20–31	M	Athlete (sepak takraw)	3-d weighed food record	100.0 mg	85.7	171.4
Chee et al. [32], 1996	Malaysia	37–39 (18–58)	M/F	Healthy Malay and Indian Adults	3-d weighed food record	25.0–29.0 mg	27.1–28.6	46.4–49.7
Moy & Rahman [89], 2002	Malaysia	40–60+	M/F	Diabetes patients	24-h recall	50.3–86.7 mg	71.9–123.9	93.4–161.0
Gan et al. [34], 2011	Malaysia	19.98–20.62	M/F	University students	2-d 24-h dietary recall	42.0–46.5 mg	60.0–66.4	72.0–86.4
Karupaiah et al. [35], 2013	Malaysia	19–65	F	Women living in high-rise dwellings	3-d diet record	83.0–104.0 mg	118.6–148.6	148.2–193.1
Menon et al. [38], 2014	Malaysia	20–50	M/F	Newly diagnosed cancer patients	24-h recall	35.0 mg	50.0	60.0
Pirabbasi et al. [90], 2016	Malaysia	43–76	M	COPD patient; Control and Intervention; Baseline	CNAQ; CNAQ+ Vitamin C + NAC supplement; CNAQ+ Vitamin C supplement; CNAQ + NAC supplement	23.8–37.9 mg	34.0–54.1	40.8–64.9
Yang et al. [40], 2016	Malaysia	39.3 (38.6; 40.0); 41.5 (40.4; 42.7)	M/F	Healthy adults; Urban population	2 × 24-h food recall	28.5–28.7 mg	30.2–38.0	36.3–45.6
Koo et al. [46], 2019	Malaysia	18–29	M/F	Healthy adults; Urban population	3-d 24-h food recall	19.7–25.4 mg	28.1–36.3	36.6–47.2
Chai et al. [47], 2019	Malaysia	30 to >50	F	Adult vegetarians	3-d 24-h food recall	190.8 mg	272.6	354.3
Chong & Appanah [48], 2019	Malaysia	19–59	F	Women reproductive age; Healthy adults; Rural population	2 × 24-h food recall	50.3–75.0 mg	71.9–107.1	93.4–139.3
Shahar et al. [53], 2021	Malaysia	56.3 (6.5)	M/F	Validated method	3-d food recall	57.1 mg	81.6	106.0
Mognard et al. [58], 2023	Malaysia	18 to ≥60	M/F	Urban and rural population	24-h recall	45.6 mg	65.1	84.7
Ghazali & Isa [59], 2023	Malaysia	22.8 ± 0.9	M/F	Validated method in young adults in university	3-d food record	20.5 mg	29.3	38.1
**21. Zinc**								
Pakasi et al. [62], 2009	Indonesia	28 (22–39)	M/F	Tuberculosis patients	24-h recall; SQ-FFQ	3.3 mg	34.7	41.7
Muhammad et al. [18], 2019	Indonesia	41.6 ± 10.2 (19–56)	M/F	Healthy adults; Urban population	SQ-FFQ	9.6 mg	101.0	121.1
Kasuma [92], 2019	Indonesia	21.15 and 23.39	M/F	Minangkabau Ethnics	FFQ	5.4 mg	56.5	67.8
Gusnedi et al. [22], 2020	Indonesia	39.5 (21–44); 35.5 (21–44)	F	Food-Based Recommendation and Non-Food-Based Recommendation (Baseline)	SQ-FFQ	6.4–6.4 mg	79.6–80.0	95.6–96.0
Nuryani et al. [23], 2021	Indonesia	16–45	M/F	Adults with normal weight; Adults with central obesity	SQ-FFQ	7.1–7.8 mg	73.7–80.7	88.5–96.8
Syauqy et al. [25], 2021	Indonesia	54.8 ± 9.6	M/F	Validated method	9-d food recall	6.9 mg	72.6;	87.2
Widhani et al. [67], 2022	Indonesia	18–60	M/F	SLE Patients (Synbiotic Group); SLE Patients (Control Group); Baseline	FFQ	4.9–5.3 mg	50.0–54.0	59.9–64.7
Sakai et al. [29], 2022	Indonesia	19–29	M	University Students	5 d of observation questionnaire	4.5–6.2 mg	56.3–56.4	67.5–67.6
Khusun et al. [30], 2023	Indonesia	18–88	M/F	Adults in Urban and rural population	24-h recall	6.2 mg	63.6	76.3
Ismail et al. [71], 2012	Malaysia	18–30	M/F	Acne vulgaris patients (case) and control	3-d food diaries	4.7–6.7 mg	100.0–101.5	120.0–121.8
Teng et al. [81], 2013	Malaysia	50–70	M	Healthy men	7-d food intake using DHQ	5.4 mg	84.4	101.3
Lee & Wan Muda [44], 2019	Malaysia	20–65	M/F	Healthy adults; Urban population	3-d 24-h food recall	4.8 mg	87.0	104.4
Koo et al. [46], 2019	Malaysia	18–29	M/F	Healthy adults; Urban population	3-d 24-h food recall	4.2–5.2 mg	78.8–89.4	94.6–107.2
Chai et al. [47], 2019	Malaysia	30 to >50	F	Adult vegetarians	3-d 24-h food recall	5.1 mg	110.2	132.3
Chong & Appanah [48], 2019	Malaysia	19–59	F	Women reproductive age; Healthy adults; Rural population	2 × 24-h food recall	2.4–2.7 mg	51.1–58.7	61.3–70.4
Mognard et al. [58], 2023	Malaysia	18 to ≥60	M/F	Urban and rural population	24-h recall	7.5 mg	136.0	163.1
Ghazali & Isa [59], 2023	Malaysia	22.8 ± 0.9	M/F	Validated method in young adults in university	3-d food record	3.7 mg	65.5	78.6

Abbreviations: CNAQ, Council on Nutrition Appetite Questionnaire; COPD, chronic obstructive pulmonary disease; DHQ, diet history questionnaire; EAR, estimated average recommendation; F, female; FFQ, food frequency questionnaire; HD, hemodialysis; HD-FFQ, hemodialysis food frequency questionnaire; M, male; NAC, N-acetylcysteine; RDA, recommended daily allowance; RE, retinol equivalents; RNI, recommended nutrient intake; SLE, systemic lupus erythematosus; SQ-FFQ, semiquantitative food frequency questionnaire.

### Energy and macronutrient (carbohydrate, protein, fat, and fiber) intake

A total of 63 studies examined energy intake, 26 studies among Indonesians [[14], [15], [16], [17], [18], [19], [20], [22], [23], [24], [25], [27], [28], [29], [30], [31], [62], [63], [64], [65], [67], [68]] and 37 studies among Malaysian adults [[21], [32], [33], [34], [35], [38], [39], [41], [42], [43], [44], [45], [46], [47], [48], [49], [50], [51], [52], [53], [54], [55], [56], [57], [58], [59], [60], [61], [69], [70], [71], [72], [73], [74], [75], [76], [77]]. Of the 30 studies analyzed both Indonesian and Malaysian adults exhibited insufficient energy intake ranging from 34.8% to 89.6% RDA/RNI. Five studies from Malaysia [46,49,51,52,61] and four studies from Indonesia [18,21,93,66] showed excessive energy intake with Malaysian adults consuming between 100.5% to 175.8% RNI and Indonesian adults consuming between 107.9% to 119.7% RDA. Studies assessing energy intake among patients with comorbidities (tuberculosis, cancer, type 2 diabetes, kidney disease, systemic lupus erythematosus) highlighted dissatisfaction with energy intake, which ranged from 34.8% to 79.3% RDA/RNI [62,67,28,68,17,72,50,55,75,60,38]. Several studies exploring the intake of obese female adults showed unexpected energy intake ranging from ∼66.5% to 111.3% RDA/RNI [64,65,31,20,41,45,54].

Nineteen studies conducted among Indonesian adults revealed a wide range of carbohydrate intake varying from 10.39% to 121.28% RDA [22,[23], [24], [25], [27],^28^,^29^,^31^,[16], [17], [18], [19], [20], [21],^14^]. In contrast, studies among Malaysian adults (30 studies) presented a higher carbohydrate intake than Indonesian adults, with a range of 22.9% to 226.9% RNI [[32], [33], [41],^43^,[45], [46], [47], [48], [49],[50], [51], [52], [53], [54], [55], [56], [57],[34], [35], [38], [39], [40], [58], [59], [60], [61], [71], [77],^36^,^44^]. A study by Karupaiah et al. [35] in 2013 specifically presented the nutrient intakes of women living in high-rise dwellings, which resulted in a high carbohydrate intake of 109% to 113% RNI, similarly an Indonesian study by Muhammad et al. in 2019 [66] assessed carbohydrate intake in urban adult populations, finding an intake of 121.2% RDA. Additionally, vegetarians and athletes among Malaysian adults reported a high amount of carbohydrates intake of 121% and 167.9% RNI, respectively. Carbohydrates are an essential source of energy, however excessive intake could lead to weight gain and other health problems [47,49]. Among 30 studies examining fiber intake in Indonesian and Malaysian adults, only 4 specifically focused on vegetarians, athletes, and individuals living in urban and high-density environments. These 4 studies reported adequate level of fiber intake, ranging from 80.3% to 244% of the RDA or RNI. In contrast, the remaining 26 studies indicated insufficient fiber intake, ranging from 10.7% to 72.7% of the RDA/RNI. This low fiber intake may lead to digestive problem and increased risk of chronic diseases [64,[23], [24], [25], [27], [65],^67^,^29^,^31^,^17^,^19^,^21^,^33^,^43^,^46^,^47^,^49^,^52^,^57^,^58^,^59^,^60^,^93^,^44^], as well as highlighted the low consumption of fruits and vegetables, which are the primary sources of dietary fiber, which are crucial for maintaining a healthy digestive system.

Studies examining protein intake among Indonesian and Malaysian adults revealed wide variations ranging from 42.2% to 216.8% RDA/RNI. In Malaysia, adult's protein intake varied from 67.5% to 216.8% RNI [41,55], while studies in Indonesia showed a lower range, of approximately 42.2% to 150.6% RNI [62,23]. Notably, two out of five studies [62,14,27,29,17] indicated that tuberculosis patients in Indonesia had low protein intake ranging between 42.2% and 76% of RDA). This deficiency may lead to muscle wasting and weakened immune function, particularly in severe tuberculosis patients, who are often more protein-deficient [62,17,94,95]. A study conducted by Zahra et al. [31] in Indonesia demonstrated that the median of protein intake was around 45.6 g/d, which is significantly below the recommendation of protein intake of 57 g/d. The typical sources of protein of these individuals were chicken and eggs.

A total of 52 studies examined fat intake among Indonesian and Malaysian adults ranging from 41.3% to 216.8% RDA/RNI. The lowest intake was from an Indonesian study in 2018, involving 13 tuberculosis patients, while the highest intake was reported from a Malaysian study in 2011 [34] assessed university students population. Of 31 studies in Malaysia, 16 indicated excessive fat intake measuring 103.9% to 234.6% of the RNI. While in Indonesia, 7 out of 20 studies showed similar findings ranging from 101.8% to 179.9% of the RDA. Four studies assessing omega (ω)-3 and ω-6 fatty acids among Indonesian and Malaysian adults found that the intake levels varied significantly between 34.1% to 163.6% RDA/RNI and 5.9% to 31.2% RDA/RNI, respectively [22,31,19,76,93]. Additionally, studies among Indonesian and Malaysian adults revealed a low intake of monounsaturated fatty acids (MUFAs) among obese adults ranging from 30.4% to 61.2% RDA/RNI [64,78]. Conversely, twelve studies from Indonesia and Malaysia showed varied results for saturated fatty acids (SFAs), ranging from 58.3% to 177.3% RDA/RNI. Specifically, a study from Indonesia focusing on a male adult population working at oil and gas worksites showed a high intake of SFAs ranging from 172.8% to 177.3% RDA) [31].

### Fat-soluble vitamins: vitamin A, vitamin D, vitamin E, and vitamin K

Six studies assessing vitamin A intake among Indonesian adults were conducted in both urban and rural areas [62,23,67,29,18,96], revealing a wide range of vitamin A intake from 41.5% to 391.4% RDA (58.1%–548.0% EAR). The lowest recorded intake at 282 μg, was observed among university students (18–29 y) in urban areas in 2021, indicating a potential risk of vitamin A deficiency in this population [29]. In contrast, the highest intake was found in a study among obese adults in rural areas in 2022 with a consumption of 2479 μg [30]. Similarly, 12 Malaysian studies [64,17,[21], [32], [33],^43^,[49], [69], [72],^55^,^59^,^44^] showed a wide range of vitamin A intake from 66.5% to 337.2% RNI (93.1%–472.0% EAR). One study among Malay and Indian female adults conducted in rural areas in 1996 had the lowest vitamin A intake (432 μg). Conversly, a study among male athletes in urban areas in 1995 had the highest intake (2023 μg) [32,88].

Five studies [62,31,71,40,94] reported inadequate intake of vitamin D among Indonesian adults, with level ranging from 12.0% to 28.0% RDA (36.0%–84.0% EAR), except for 1 study in rural areas of North Sumatera (116.0% RDA [348.0% EAR]) [94]. Likewise, 4 Malaysian studies primarily conducted in urban areas showed low vitamin D intake [58,59,95,78], ranging from 12.2% to 36.0% RNI (36.6%–108.0% EAR), which could potentially lead to vitamin D deficiency and related health issues.

Five studies reported inadequate vitamin E intake among Indonesian adults [23,25,67,29,93], ranging from 18.3% to 55.3% RDA (22.6%–68.4% EAR). Only 3 studies explored vitamin E intake in Malaysian adults [58,71,90], with the lowest intake of 7.20% RNI (9.36% EAR) [58] and the highest of 59.0% RNI (75.23% EAR) [71].

A study observing vitamin K intake among Indonesian populations showed adequate intake among university students, ranging from 140.9% to 144.0% RDA (76.3%–88.0% EAR), highlighting the need for further research. No data were available on the intake of vitamin K among Malaysian adults. According to the retrieved studies, the main sources of vitamin A, D, and K among Indonesian male and female university students were eggs, and vitamin E came mostly from oils and fats. This finding opens avenues for future studies to explore the dietary sources of these vitamins in more detail [29]. Jati et al. 2014 [96] reported different findings: dark green leafy vegetables were the primary source of vitamin A for women of childbearing age in Indonesia. Among Malaysian adults, the source of vitamin D was mainly from fresh oily fish (56%) [83].

### Water-soluble vitamins: vitamin B (thiamine, riboflavin, niacin, pyridoxine, cobalamin, folate), and vitamin C

Three studies from Indonesia [25,29,93] and 11 studies from Malaysia [32,46,48,58,59,34,35,38,40,89,88] investigated riboflavin intake in the adult population. In Malaysia, riboflavin intake among sportsmen engaged in sepak takraw (a sport played with a ball made of rattan or synthetic plastic) ranged from 18 to 56 mg per day, showcasing potential variations in dietary habits and nutritional awareness among athletes. Similarly, male and female Malaysian estate workers exhibited riboflavin intake of 52 to 56 mg, suggesting disparities in nutrient consumption patterns within rural occupational settings. Moreover, research focusing on type 2 diabetes patients attending outpatient clinics in Malaysia revealed a wide range of riboflavin intake, varying from 28 to 124 mg, emphasizing the importance of tailored dietary management for individuals with specific health conditions.

A notable difference in riboflavin intake was observed between male and female university students in Malaysia cohorts, with intake ranging from 155 to 347 mg, indicating potential disparities in dietary behaviors and nutritional knowledge among young adults. In urban settings, such as Kuala Lumpur, female adults living in high-rise dwellings exhibited riboflavin intake ranging from 5 to 85 mg, highlighting variations in dietary practices and access to nutrient-rich foods within urban environments. Conversely, newly diagnosed cancer patients from rural Malaysia demonstrated a low mean riboflavin intake (40 mg), underscoring the need for targeted nutritional support interventions in resource-limited settings. In Indonesia, riboflavin intake ranged from 10 to 503 mg daily.

Two studies from Indonesia [25,29] and 9 studies from Malaysia [32,46,48,58,34,35,38,40,93] explored thiamine consumption in the adult population. In rural and urban communities of Malaysia, the mean thiamine intake among males ranged between 0.80 and 1.39 mg. Those reports are similar to the mean intake of 0.75 mg among Indonesian males and females in this review.

Twelve studies from Malaysia [32,46,48,58,59,34,35,38,40,89,88] and 2 studies from Indonesia [25,29] examined niacin consumption among adults. Niacin consumption among male and female Malaysian adults varied, with mean intake from 8.50 to 72.50 and 5.30 to 21.70 mg, respectively. Male and female adults in Indonesia showed similar niacin intake, ranging between 6.90 and 8.10 mg.

Only three studies from Indonesia [29,93,91] reported on pyridoxine consumption among male and female adults. The consumption among university students was reported to be between 0.50 and 0.60 mg. The mean intake among nurses who consumed vitamin B supplementation was higher (9.08 mg) than that of those without supplementation (1.75 mg). Moreover, the mean intake for the male and female general population ranged from 3.31 to 4.59 mg.

Three studies from Indonesia [29,93,91] and 1 study from Malaysia [47] reported the mean cobalamin intake among vegetarian Malaysian females was lower (0.51 μg), although the highest mean intake came from a group of nurses from Indonesia who consumed vitamin B supplements (9.34 μg). Moreover, among university students, the consumption ranged from 2.50 to 2.90 μg.

Three studies from Indonesia [29,93,91] and 3 studies from Malaysia [46,47,58] investigated folate consumption ranging from 53.60 to 403 μg. The mean intake among Indonesian university students was between 103.7 and 123.7 μg. Intake of folate among vegetarian Malaysian females was higher (192.9 μg) than that of a regular diet (53.6 μg). Furthermore, nurses who consumed vitamin B supplements reported a higher mean intake (305 μg) than nurses who did not take vitamin B supplements (228.6 μg).

Fourteen studies from Malaysia [32,[46], [47], [48],^53^,^58^,^59^,^34^,^35^,^38^,^40^,[88], [89], [90]] and 5 studies from Indonesia [23,25,67,29,93] investigated vitamin C intake between 25 and 104 mg (Malaysia) and 32.9 to 152.3 mg (Indonesia). The mean intake of Malaysian adults varied between 25 and 190.8 mg (female) and from 26.84 to 100 mg (male). Furthermore, among college students in Indonesia, the mean intake was 29.20 in males and 37.50 mg in females. Regarding food sources, almost all included studies that recorded water-soluble vitamin intake did not mention the primary food source for this nutrient, especially in the adult population.

### Macrominerals: calcium, phosphorus, magnesium, sodium, potassium, and chloride

Assessment of calcium intake in the reported studies was predominantly in the Malaysian adult populations, with 23 studies conducted [32,43,[46], [47], [48],^69^,^50^,^53^,[58], [59], [76],^34^,^35^,^38^,^40^,^36^,^44^,^83^,[79], [80], [81], [82], [84]], while only 5 studies were conducted in the Indonesian adult populations [23,25,29,17,84]. The findings of calcium intake revealed a concerning trend, showing that calcium intake in adults in both countries did not meet 100% RDA/RNI/EAR. In most of the assessed studies, Malaysian adults' calcium intake ranged from ∼12.2% to 56% RNI or 23.8% to 68% EAR. Only 1 particular study by Chai et al. in 2019 [47] presented 74.5% RNI or 89.4 % EAR in vegetarian adult Malaysian women. These findings highlight the need for immediate action to address the calcium deficiency in the adult population. Likewise, calcium intake in the Indonesian population was not much different, ranging from ∼9.5% to 58% RDA or 11.4% to 70% EAR.

Phosphorus intake among Indonesian adults was 75% to 156% RDA (90%–156% EAR) [27,31]. In contrast, phosphorus intake among the Malaysian adult population met ∼90% RNI (90%–180% EAR) [47,55]. Magnesium intake in 2 studies conducted on Malaysian adults illustrated intake <60% RNI (31%–60% EAR) [[44], [58]]. Meanwhile, magnesium intake among the Indonesian adult population was in the minimum range of 33.6% to 112% RDA (33.1%–106% EAR) [29,93].

Three studies among Indonesian adults showed adequate sodium intake, with a minimum of 91% RDA or 91.2% to 212% EAR [24,68,29]. Only 1 specific study in rural areas showed insufficient sodium intake <30% RDA (25%–28% EAR) [23]. In more Malaysian studies, sodium intake was ≥85% RNI (85%–198% EAR) [43,50,53,58,59,34,35,[36], [44],^79^,^80^,^85^,^87^]. In the dialysis patient population in Malaysia, sodium intake slightly exceeded 100% EAR (129%–159% RNI/EAR) [50,36].

Potassium intake among adult population in Malaysia was below its national nutrient intake recommendations (12%–46% RNI/EAR) [33,58]. In similar results, potassium intake in Indonesian adults was between 20% to 70.8% RDA (22.3%–70.8% EAR) [25,29]. In the retrieved studies, no data addressed chloride intake in both countries.

### Trace elements: iron, manganese, copper, iodine, zinc, cobalt, fluoride, and selenium

Iron and zinc were the 2 most researched trace elements. Iron intake in the Indonesian adult population varied significantly. Three Indonesian studies revealed iron intake that surpasses 100% EAR (108%–165% RDA or 174%–263.3% EAR) [25,30,93]. Otherwise, in 3 other studies [22,23,29], the iron intake was below 90% EAR (20%–73.6% RDA or 20%–87.1% EAR). Iron intake among Malaysian adults ranged from 29% to 234% RNI (47%–374% EAR) [32,76]. As for zinc, the intake in Malaysian adults was within 51% to 136% RNI (61%–163% EAR) with women of reproductive age residing in rural areas having the lowest zinc intake [48,58]. In Indonesian adults, the tendency for zinc intake was lower, around 34% to 80% RDA (41%–97% EAR). The tuberculosis patient population exhibited a significantly lower zinc intake than the other populations [14,23]. A study by Muhammad et al. in 2019 [93] investigated zinc intake among healthy adults in urban areas of Indonesia and found that healthy adults achieved 100% RDA (121.1% EAR). Furthermore, Mognard et al. 2023 [58] conducted a study on Malaysian adults and found that breakfast accounted for over 30% of their daily calcium and potassium intake. However, it negatively affected their sodium, iron, and zinc intake levels. Additionally, a study by Sakai et al. in 2022 [29] on university students in Indonesia revealed that rice products, such as cereals, were the primary source of their iron intake.

Data on manganese, copper, and selenium intake among adult populations of Indonesia and Malaysia has limited study sources. A single study was conducted in each country for manganese intake, resulting in an inversely proportional one. Inadequate manganese intake is found among Malaysian adults in urban areas (<20% RNI/EAR) [44]. Meanwhile, according to a study of university students, manganese intake in Indonesian adults is ∼88% to 96% RDA/EAR [29]. Copper intake in 3 studies illustrated that intake does not meet 100% EAR in both countries (64%–89% RDA/EAR for Indonesians and 55.56% for Malaysian adults) [25,[29], [44]].

Dietary selenium intake among Indonesian adults exceeded 100% of the national recommended nutritional intake (158%–450% EAR) [93,86]. In contrast, selenium intake assessed in Malaysian studies was within the range of 56% to 155% RNI (67.5%–186% EAR) [32,69]. No data on the intake of iodine, cobalt, and fluoride among the adult population in Indonesia and Malaysia are available.

### Meta-analysis

#### Macronutrients (protein and fat)

**FIGURE 2:**
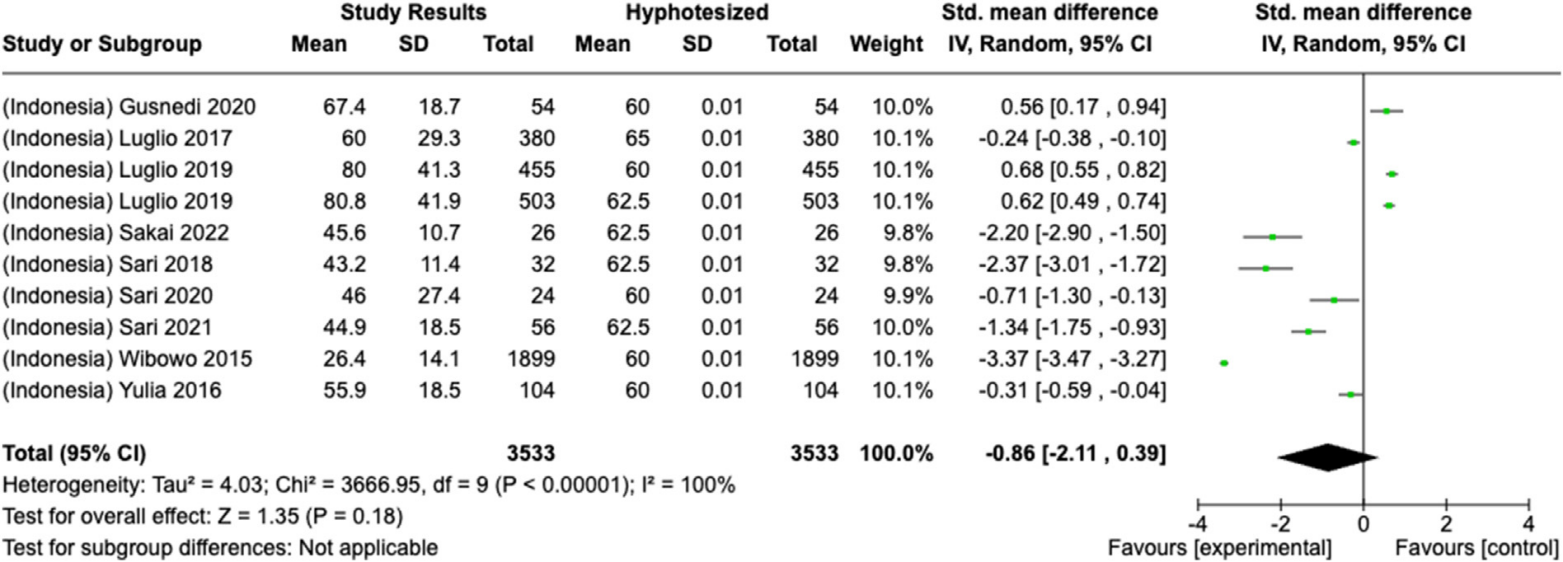
Protein intake among Indonesian adults. Values are presented as standardized mean differences (SMDs) and confidence interval (CI) comparing protein intake across all included studies in Indonesia against the recommended daily allowance (RDA) of Indonesian adults. The variability of results among the studies is expressed using Tau-squared (Tau^2^), Chi-squared (Chi^2^), or I-squared (I^2^).

**FIGURE 3:**
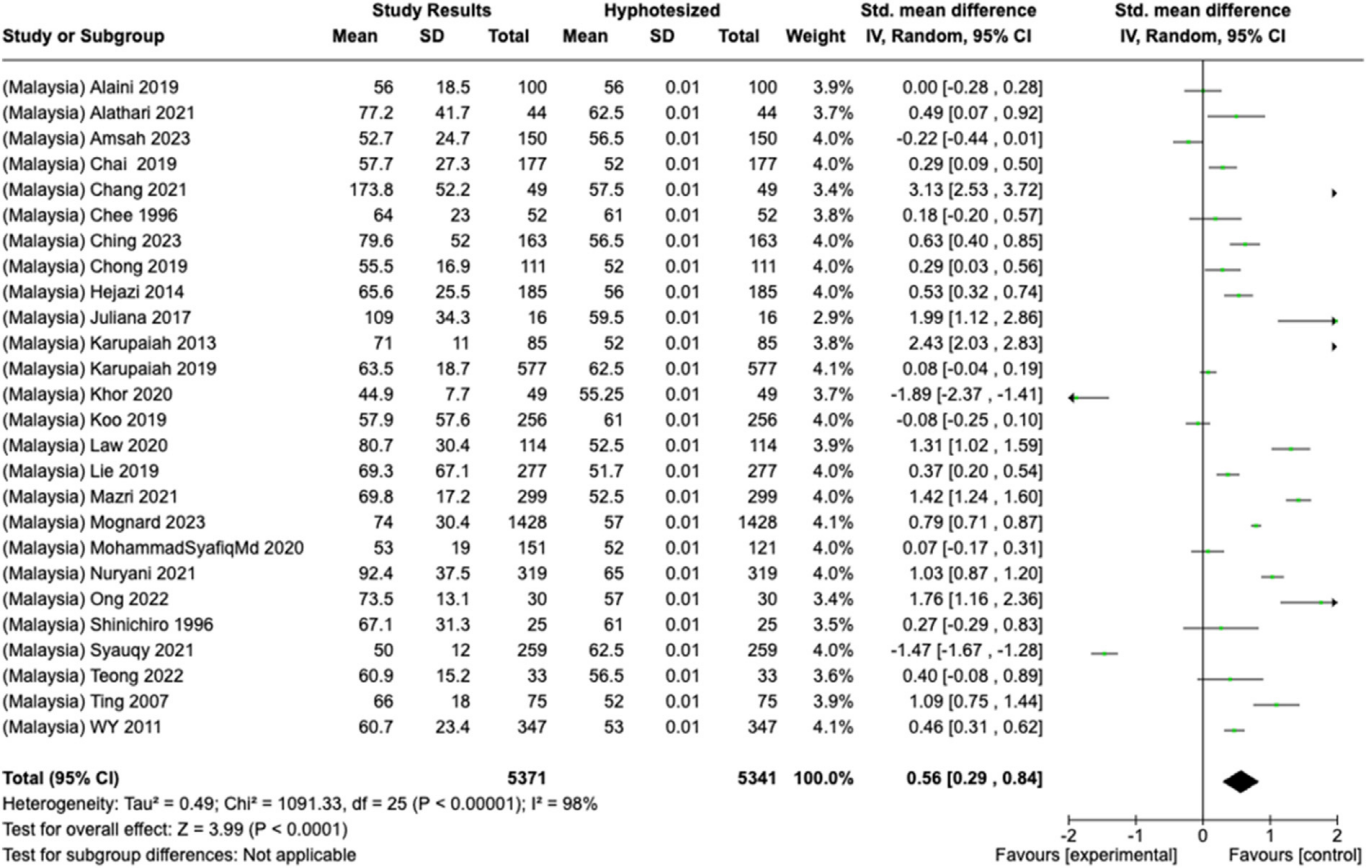
Protein intake among Malaysian adults. Values are presented as standardized mean differences (SMDs) and confidence interval (CI) comparing protein intake across all included studies in Malaysia against the recommended nutrient intake (RNI) of Malaysian adults. The variability of results among the studies is expressed using Tau-squared (Tau^2^), Chi-squared (Chi^2^), or I-squared (I^2^).

The meta-analysis performed in this study was stratified by country. Figure 2 presents the protein intake among Indonesian adults that did not meet the recommendation, as SMD was −0.86 (95% CI: −2.11, 0.39). On the contrary, Figure 3 shows a meta-analysis of protein intake among Malaysian adults. It highlights that overall intake was more than the national recommendation, as indicated by an SMD of 0.56 (95% CI: 0.29, 0.84). The overall protein intake in both countries was adequate [SMD: 0.18; 95% CI: −0.27, 0.64].

**FIGURE 4:**
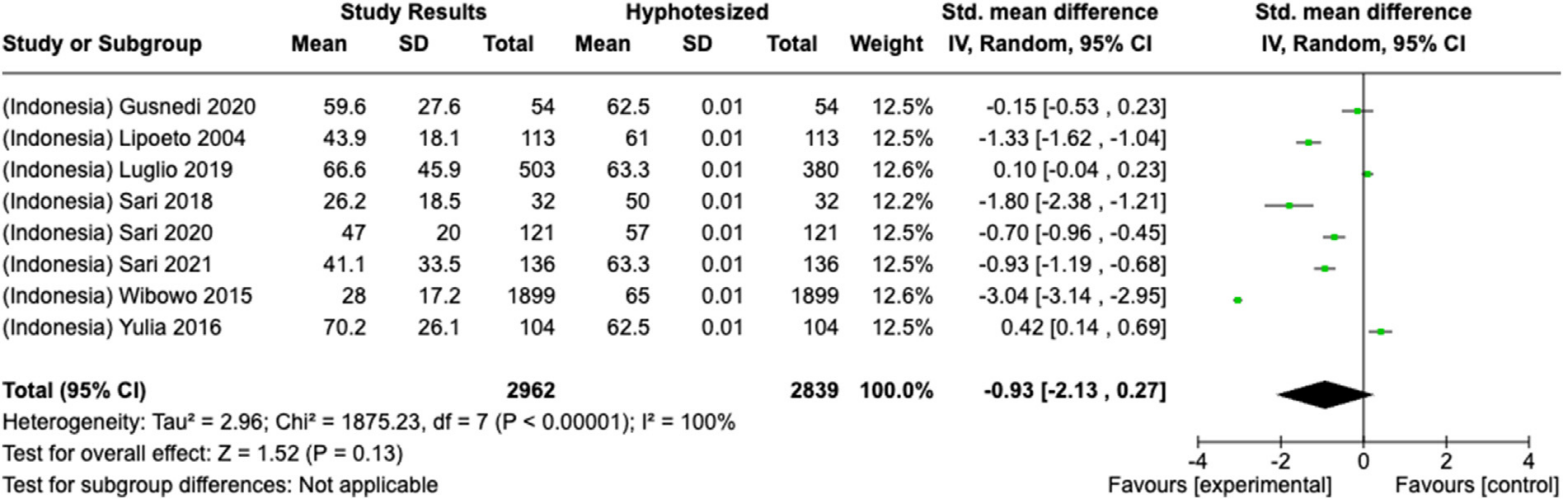
Fat intake among Indonesian adults. Values are presented as standardized mean differences (SMDs) and confidence interval (CI) comparing fat intake across all included studies in Indonesia against the recommended daily allowance (RDA) of Indonesian adults. The variability of results among the studies is expressed using Tau-squared (Tau^2^), Chi-squared (Chi^2^), or I-squared (I^2^).

**FIGURE 5:**
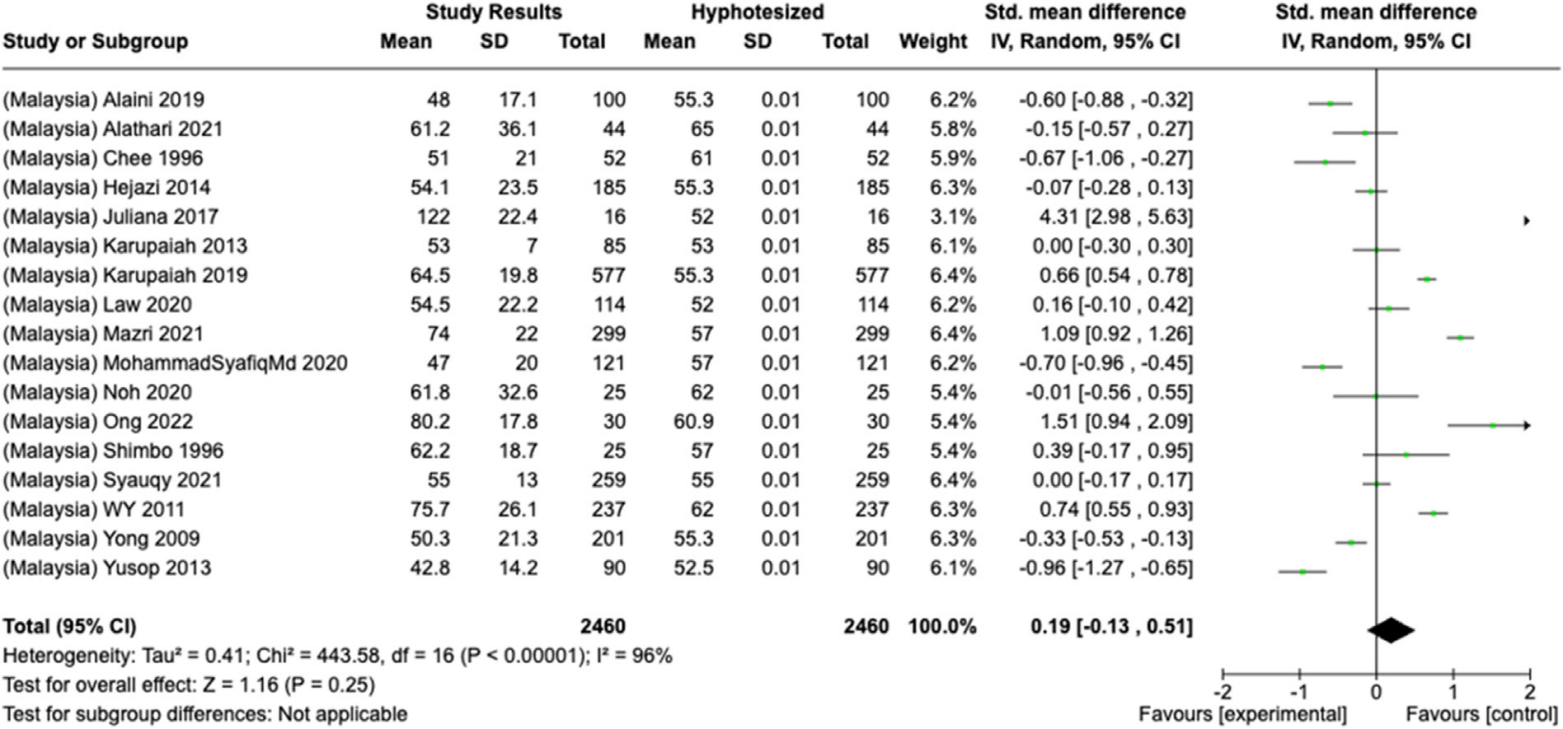
Fat intake among Malaysian adults. Values are presented as standardized mean differences (SMDs) and confidence interval (CI) comparing fat intake across all included studies in Malaysia against the recommended nutrient intake (RNI) of Malaysian adults. The variability of results among the studies is expressed using Tau-squared (Tau^2^), Chi-squared (Chi^2^), or I-squared (I^2^).

Figure 4 presents a meta-analysis of 8 studies determining that fat intake among Indonesian adults was below the recommendation (SMD: −0.93; 95% CI: −2.13, 0.27). Similarly, Figure 5 suggests the fat intake among Malaysian adults is higher than the recommendation, with an SMD of 0.19 (95% CI: −0.13, 0.51)

#### Micronutrients (thiamine, pyridoxine, calcium, zinc, iron, and sodium)

**FIGURE 6:**
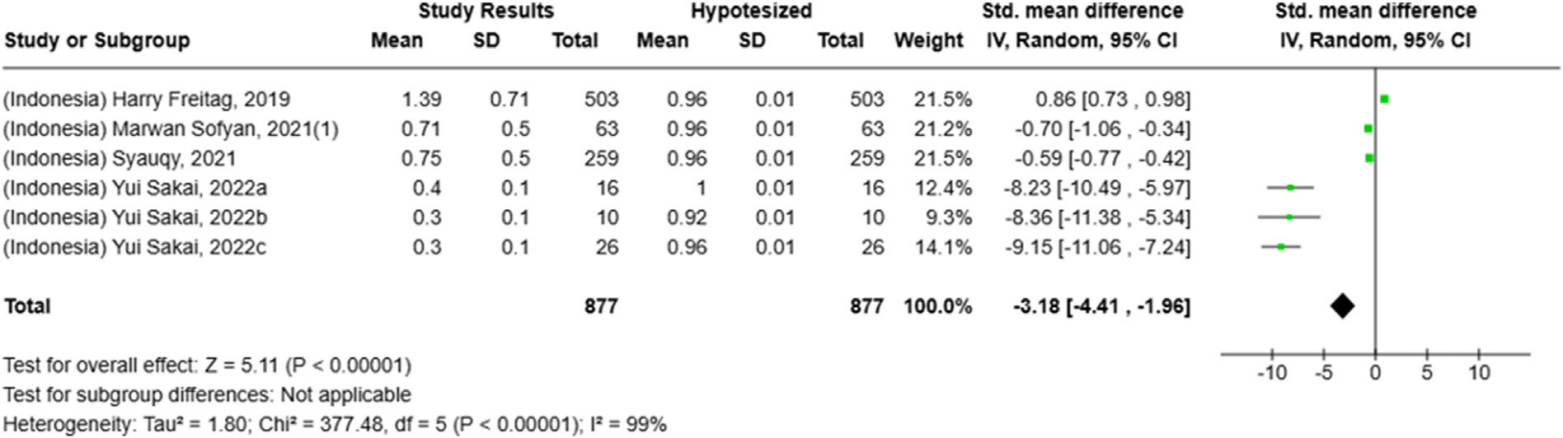
Thiamine intake among Indonesian adults. Values are presented as standardized mean differences (SMDs) and confidence interval (CI) comparing thiamine intake across all included studies in Indonesia against the recommended daily allowance (RDA) of Indonesian adults. The variability of results among the studies is expressed using Tau-squared (Tau^2^), Chi-squared (Chi^2^), or I-squared (I^2^).

**FIGURE 7:**
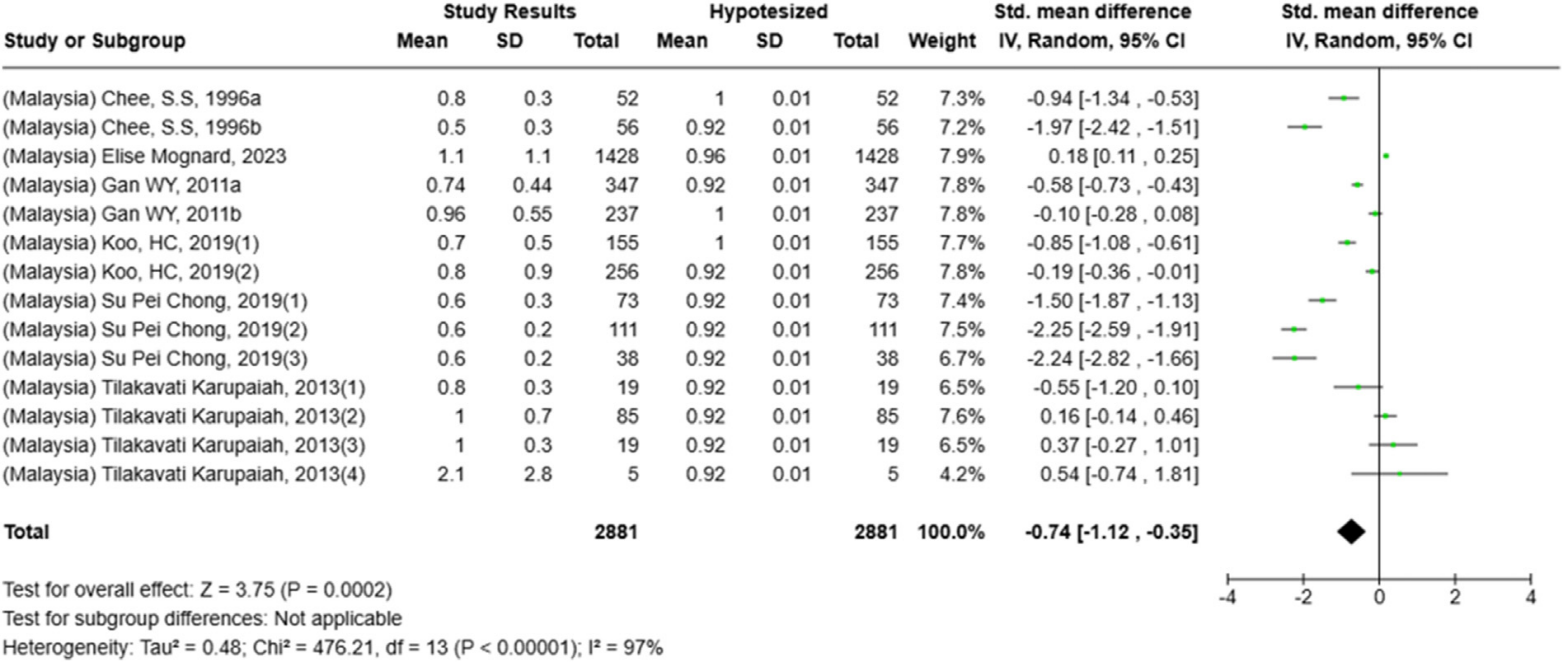
Thiamine intake among Malaysian adults. Values are presented as standardized mean differences (SMDs) and confidence interval (CI) comparing thiamine intake across all included studies in Malaysia against the recommended nutrient intake (RNI) of Malaysian adults. The variability of results among the studies is expressed using Tau-squared (Tau^2^), Chi-squared (Chi^2^), or I-squared (I^2^).

**FIGURE 8:**
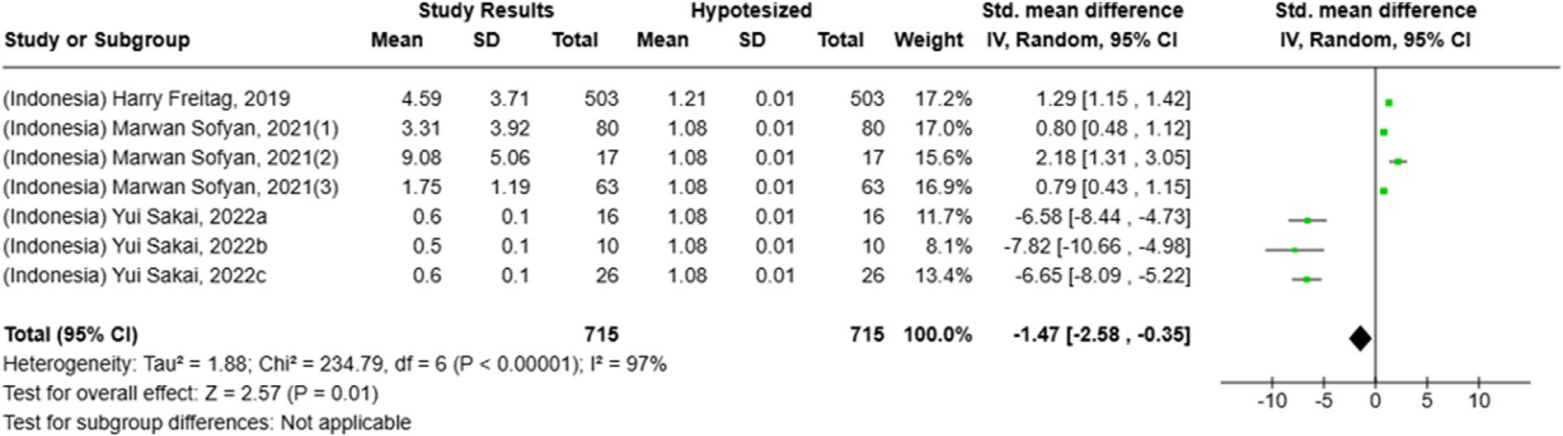
Pyridoxine intake among Indonesian adults. Values are presented as standardized mean differences (SMDs) and confidence interval (CI) comparing pyridoxine intake across all included studies in Indonesia against the recommended daily allowance (RDA) of Indonesian adults. The variability of results among the studies is expressed using Tau-squared (Tau^2^), Chi-squared (Chi^2^), or I-squared (I^2^).

A meta-analysis in 4 Indonesian studies, as shown in Figure 6, revealed low thiamine consumption among Indonesians (SMD: −3.18; 95% CI: −4.41, −1.96). Similarly, thiamine intake among Malaysian (6 studies) demonstrated a lower intake compared to 100% EAR (SMD: −0.74; 95% CI: −1.12, −0.35) (Figure 7). Studies that presented mean and SD of pyridoxine intake were only available from Indonesia, as shown in Figure 8; 3 studies were included in the meta-analysis, resulting in an insufficiency of pyridoxine (vitamin B6) intake among Indonesian adults (SMD: −1.47; 95% CI: −2.58, −0.35) in a total of 715 samples. No study from Malaysia was included in the meta-analysis.

**FIGURE 9:**
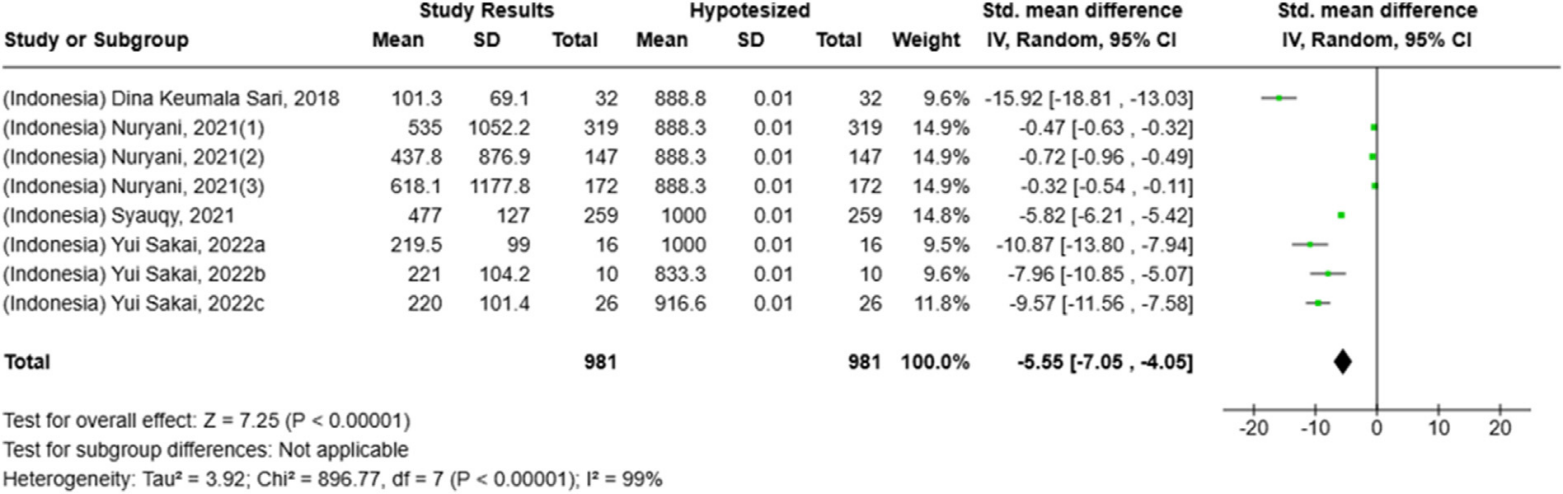
Calcium intake among Indonesian adults. Values are presented as standardized mean differences (SMDs) and confidence interval (CI) comparing calcium intake across all included studies in Indonesia against the recommended daily allowance (RDA) of Indonesian adults. The variability of results among the studies is expressed using Tau-squared (Tau^2^), Chi-squared (Chi^2^), or I-squared (I^2^).

**FIGURE 10:**
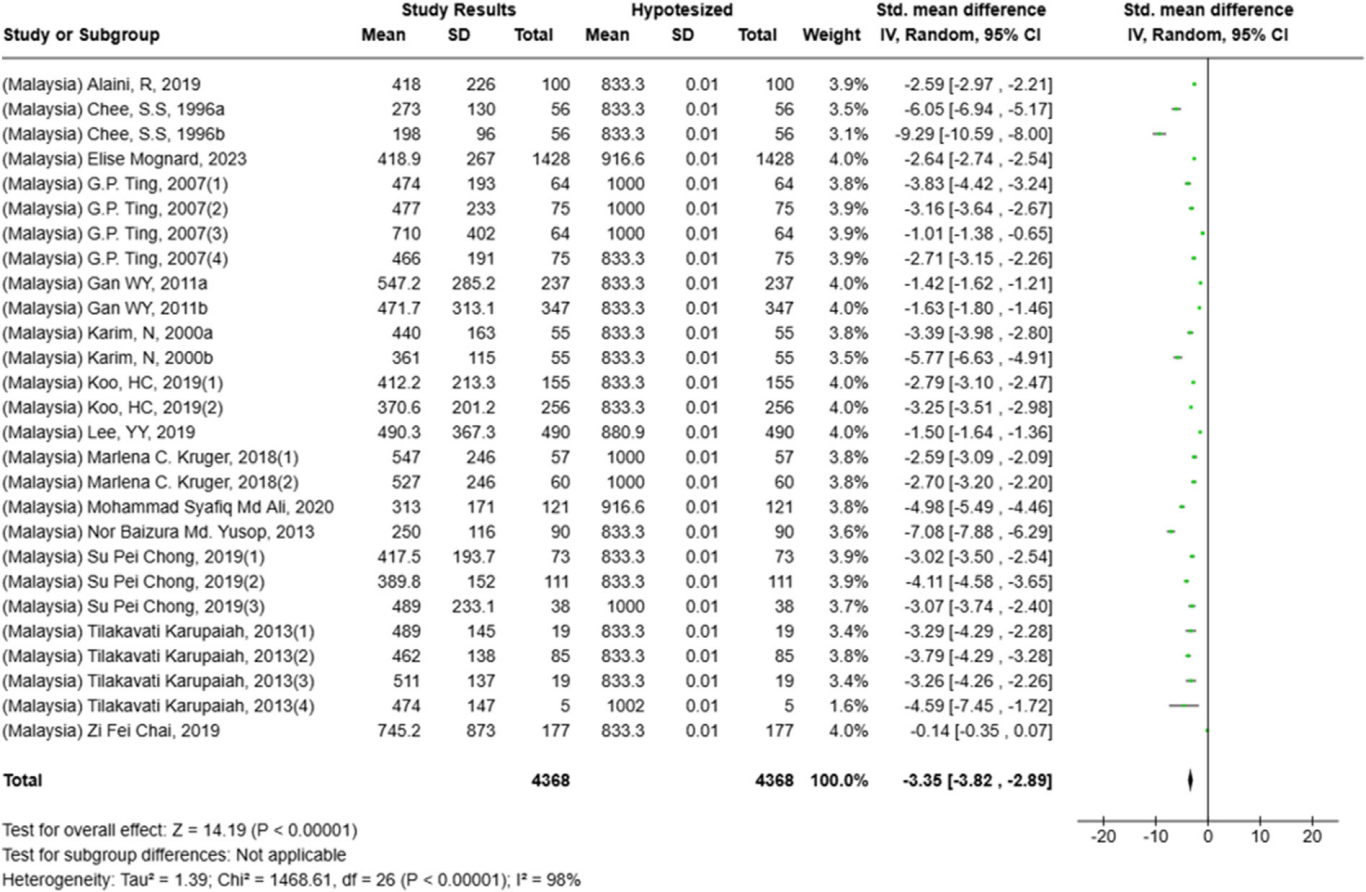
Calcium intake among Malaysian adults. Values are presented as standardized mean differences (SMDs) and confidence interval (CI) comparing calcium intake across all included studies in Malaysia against the recommended nutrient intake (RNI) of Malaysian adults. The variability of results among the studies is expressed using Tau-squared (Tau^2^), Chi-squared (Chi^2^), or I-squared (I^2^).

Figure 9 shows there is low calcium intake among Indonesian adults compared with 100% EAR (SMD: −5.55; 95% CI: −7.05, −4.05), with mean daily intake deficit of 573.46 mg. Additionally, the Malaysian adults showed similar results of insufficient calcium intake, with SMD: −3.35 (95% CI: −3.82, −2.89), as shown in Figure 10. Overall, calcium intake in both countries was reported as insufficient in 18 studies (*N* = 5394) (SMD: −3.69; 95% CI: −4.18, −3.19).

**FIGURE 11:**
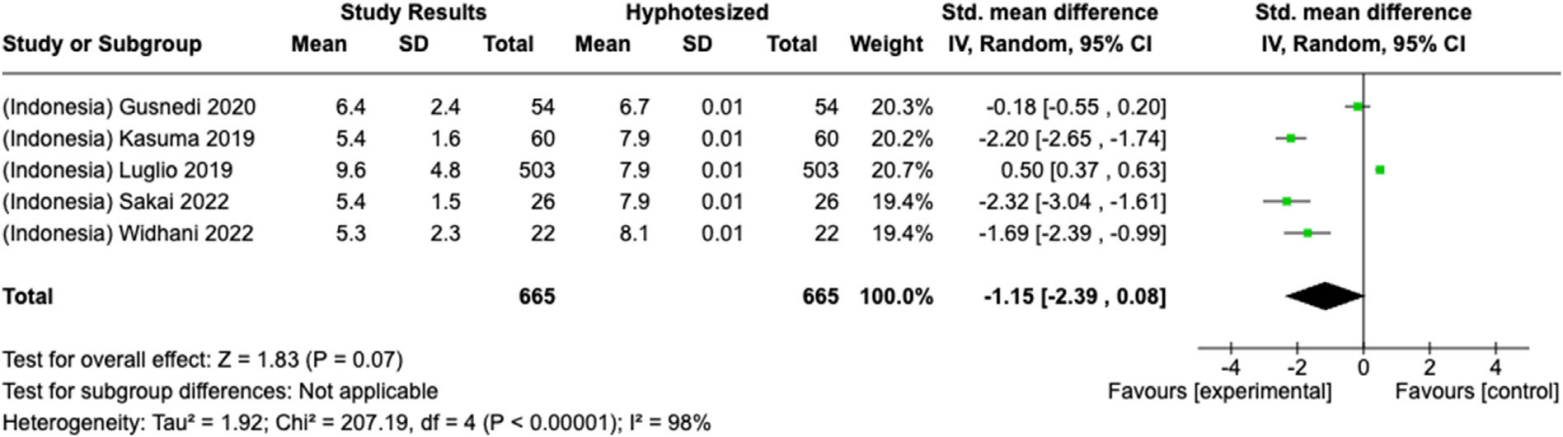
Zinc intake among Indonesian adults. Values are presented as standardized mean differences (SMDs) and confidence interval (CI) comparing zinc intake across all included studies in Indonesia against the recommended daily allowance (RDA) of Indonesian adults. The variability of results among the studies is expressed using Tau-squared (Tau^2^), Chi-squared (Chi^2^), or I-squared (I^2^).

**FIGURE 12:**
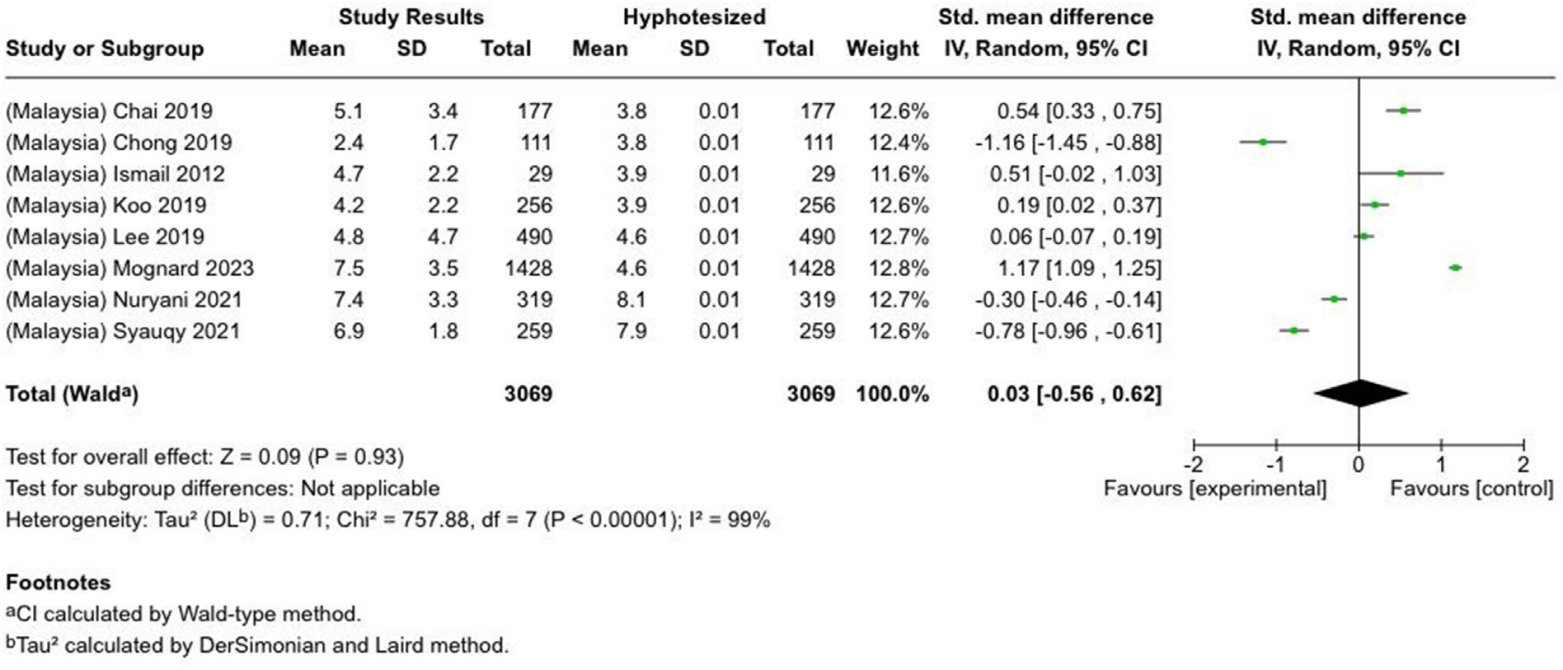
Zinc intake among Malaysian adults. Values are presented as standardized mean differences (SMDs) and confidence interval (CI) comparing zinc intake across all included studies in Malaysia against the recommended nutrient intake (RNI) of Malaysian adults. The variability of results among the studies is expressed using Tau-squared (Tau^2^), Chi-squared (Chi^2^), or I-squared (I^2^).

In addition**,** Figure 11 depicts zinc intake among Indonesian adults, indicating a lower intake of zinc, which was <100% EAR (SMD: −1.15; 95% CI: −2.39, 0.08). Apart from that, Indonesian adults demonstrated a lower intake of zinc, which was <100% EAR, as shown in Figure 12.

**FIGURE 13:**
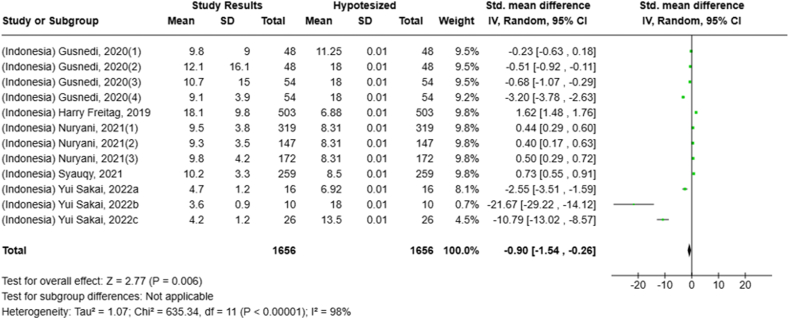
Iron intake among Indonesian adults. Values are presented as standardized mean differences (SMDs) and confidence interval (CI) comparing iron intake across all included studies in Indonesia against the recommended daily allowance (RDA) of Indonesian adults. The variability of results among the studies is expressed using Tau-squared (Tau^2^), Chi-squared (Chi^2^), or I-squared (I^2^).

**FIGURE 14:**
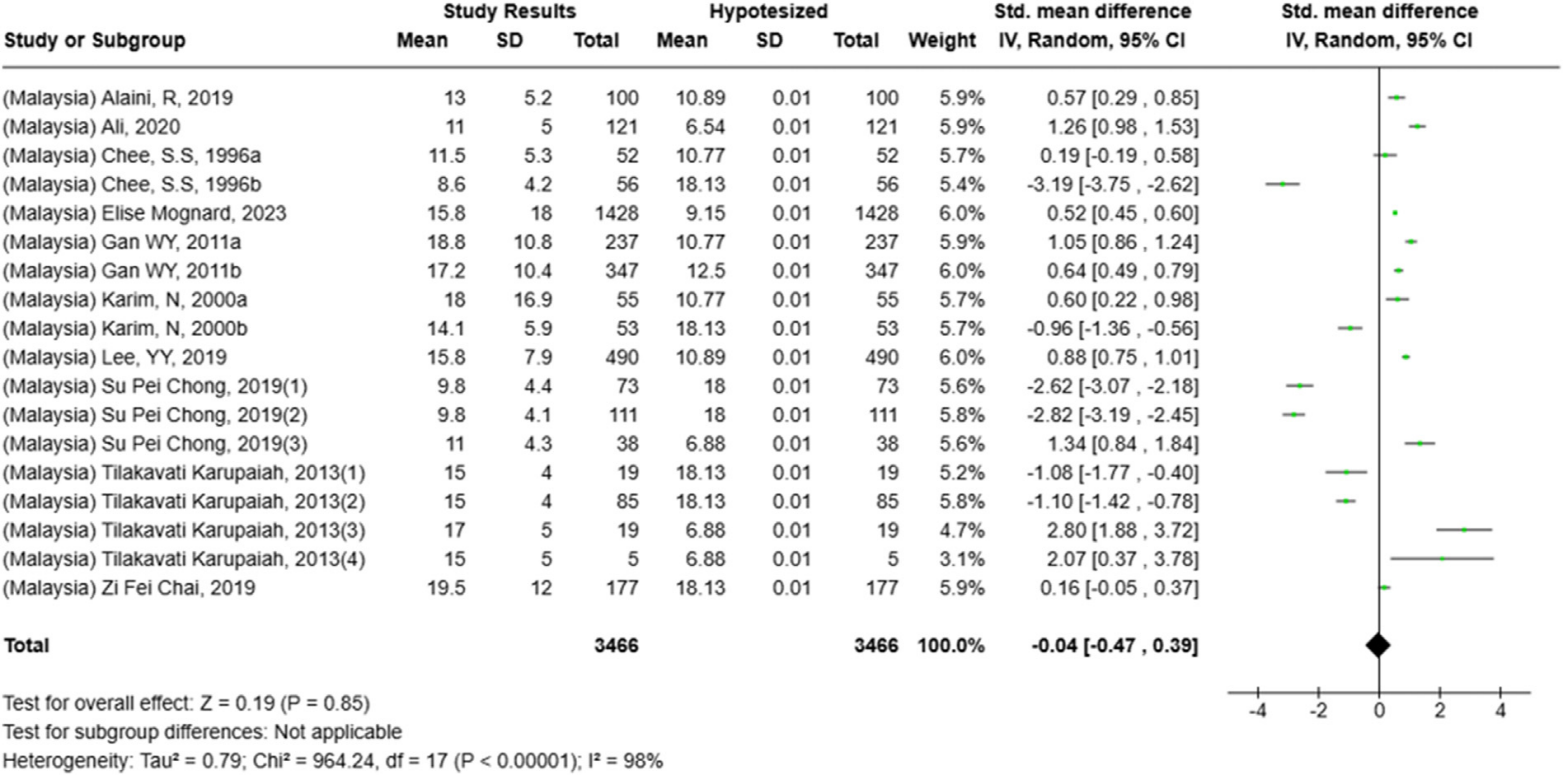
Iron intake among Malaysian adults. Values are presented as standardized mean differences (SMDs) and confidence interval (CI) comparing iron intake across all included studies in Malaysia against the recommended nutrient intake (RNI) of Malaysian adults. The variability of results among the studies is expressed using Tau-squared (Tau^2^), Chi-squared (Chi^2^), or I-squared (I^2^).

A meta-analysis of 5 studies (Figure 13) of iron intake among Indonesian adults shows a lower intake of iron compared with 100% EAR (SMD: −0.90; 95% CI: −1.54, −0.26), with mean deficit of 2.68 mg/d. Of 10 studies among Malaysian adults, the meta-analysis showed slight inadequacy (SMD: −0.04; 95% CI: −0.47, 0.39) (Figure 14).

**FIGURE 15:**
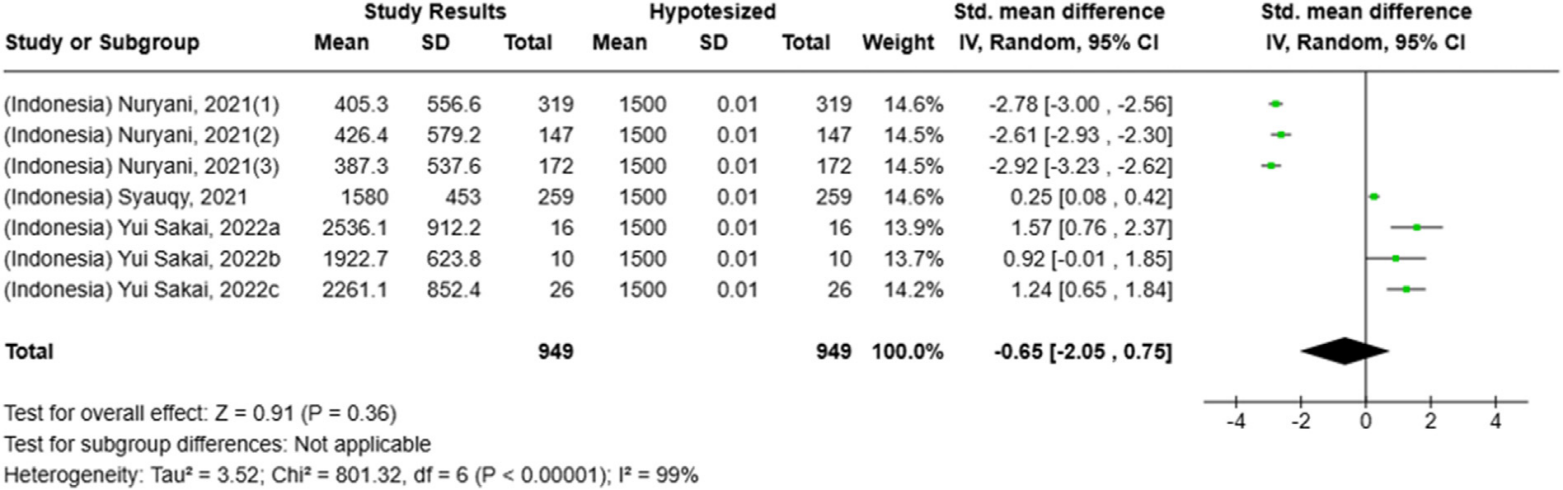
Sodium intake among Indonesian adults. Values are presented as standardized mean differences (SMDs) and confidence interval (CI) comparing sodium intake across all included studies in Indonesia against the recommended daily allowance (RDA) of Indonesian adults. The variability of results among the studies is expressed using Tau-squared (Tau^2^), Chi-squared (Chi^2^), or I-squared (I^2^).

**FIGURE 16:**
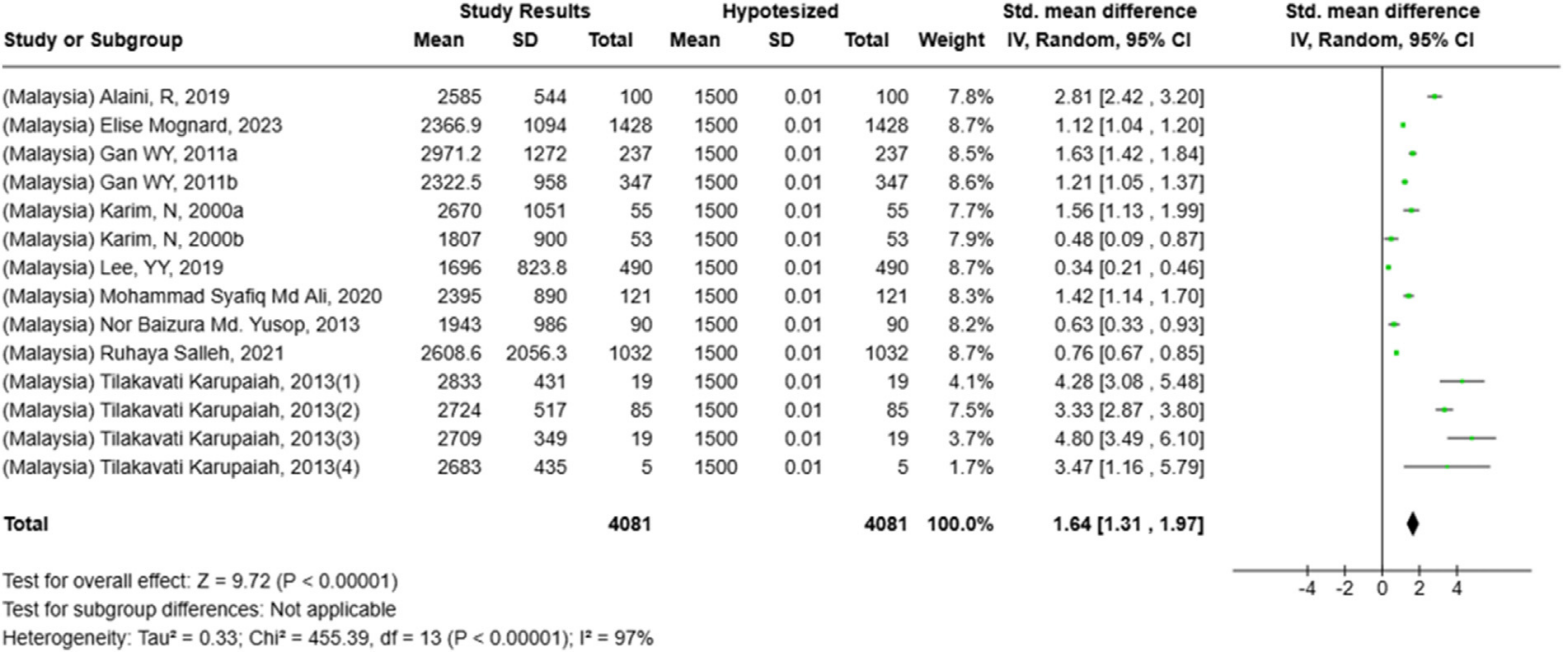
Sodium intake among Malaysian adults. Values are presented as standardized mean differences (SMDs) and confidence interval (CI) comparing sodium intake across all included studies in Malaysia against the recommended nutrient intake (RNI) of Malaysian adults. The variability of results among the studies is expressed using Tau-squared (Tau^2^), Chi-squared (Chi^2^), or I-squared (I^2^).

Meta-analysis of sodium intake in Indonesia and Malaysia revealed contrasting results. As shown in Figure 15, Indonesian studies showed deficient sodium intake below 100% EAR with mean deficit of 168.36 mg/d (SMD: −0.65; 95% CI: −2.05, 0.75). In contrast, all 9 Malaysian studies demonstrated excessive sodium intake by mean of 948.36 mg/d (SMD:1.64; 95% CI: 1.31, 1.97) (Figure 16). However, the only study that reported insufficiency from Indonesia was conducted by Nuryani et al. in 2021 [23]. The food frequently consumed in the study was rice, fresh fish, kale, tomato, chili, coconut oil, and palm oil. Rice, being the staple food, was consumed at every mealtime, while protein sources mostly came from fresh fish such as fish float, bonito, tuna, herring, and mackerel.

## Discussion

This review found that Indonesian and Malaysian adults had a diverse range of energy, carbohydrate, protein, and fat intake. Malaysians generally had a higher intake of macronutrients compared to Indonesians. Potein intake among Malaysian exceeded the recommendation, but was insufficient among Indonesian adults. Fiber intake were lower than 80% RDA/RNI in both countries. Most fat-soluble vitamins were also below 100 % EAR, although vitamin A showed a varied intake. Water-soluble vitamin intake among Indonesian and Malaysian adult populations varied widely with disparities influenced by factors such as occupation, health status, dietary habits, and supplementation. Calcium and potassium intake in both countries as well as magnesium intake in Malaysia could not meet 100% of the EAR. Furthermore, intake of phosphorus and sodium among Malaysian adults is ≥85% EAR. Iron and zinc intake among Indonesian adults were mostly below 100% EAR; in contrast, selenium intake exceeds 100% of EAR. In Malaysia, the intake of these nutrients also varied. Manganese and copper intake for adults in both countries were below 100% of the EAR indicating insufficiency. Information on vitamin K, chloride, manganese, copper, and selenium, iodine, cobalt, and fluoride intake were lacking.

### Energy and macronutrient intake

This review showed the wide range of energy intake among Indonesian and Malaysian adults. In a study by Mohd Noh et al. in 2020 [72] among Malaysian athletes, the energy intake exceeded the RDA, which is in line with their higher energy expenditure compared to nonathletes. However, almost half of the studies showed energy intake deficiency [97]. Comparably, previous studies showed dissatisfaction with energy intake, <80% RDA in other Southeast Asian countries such as Thailand [98] and Myanmar [99]. One factor reducing daily energy intake is frequent skipping meals due to the perceived lack of time or diet among the adult population [100], resulting in decreased body weight but lower diet quality and negative impact on health [101]. Irregular meal patterns is also directly associated with higher waist circumference, lower high-density lipoprotein cholesterol, insulin resistance, and metabolic syndrome [102].

A subtle carbohydrate and protein intake has been observed among Indonesian and Malaysian adults; in contrast, several studies showed that fat intake tends to increase gradually. The high intake of fat mostly came from individuals who were obese or overweight [41,45]. Most of the increased calorie consumption was obtained from fat, although lower or stable amounts of carbohydrate and protein intake were also captured in rural and urban areas of Vietnam [103]. Similarly, a 13-y longitudinal study in China demonstrated a tendency for a higher percentage of energy from fat, resulting in a high risk of obesity [104]. A previous study by Edwards et al. in 2011 [105] suggested the effects of high-fat diets on brain damage, such as increased time of reaction and decreased attention power; this finding aligned with a review by Tan and Norhaizan in 2019 [106] showing an association of high-fat diets with Alzheimer’s disease, mild cognitive impairment, or dementia [97,98]. A previous review in 2022 mentioned that the number of dietary carbohydrates and fats was associated with better and worse sleep quality (deep sleep, rapid eye movement sleep, sleep efficiency, latency, and wake after sleep onset). However, the kinds of carbohydrates and fat have different risk factors [107]. This study found a low intake of MUFAs among obese Indonesian and Malaysian adults. This aligns with a previous study by Agraib et al. 2023 [108] that showed male and female obese adults have a low intake of energy, carbohydrates, fibers, SFAs, MUFAs, and PUFAs. A previous study in 2021 presented the benefits of rich dietary MUFAs, which have anti-inflammatory effects compared with SFA-rich diets [109]. Another study in 2017 showed that the MUFA proportion found in the adipose tissue (subcutaneous and visceral) was inversely correlated with inflammation, insulin resistance, and type 2 diabetes related to obesity [110]. Meanwhile, a high intake of SFAs is associated with more body fat and reduced satiety than diets richer in PUFAs [108]. Nutrition transitions include the increased consumption of dairy products, vegetable oils, and red meat, resulting in higher intake of SFAs.

This study found that nearly half of all studies from Indonesia and Malaysia revealed relatively insufficient energy intake. Among healthy populations, higher energy intake was observed among particular subgroups such as athletes and urban populations, reflecting increased activity levels and dietary adequacy. Higher energy intake was also observed in healthy populations compared to those with clinical conditions. For instance, tuberculosis patients in Indonesia showed a low energy intake of 1113 kcal according to Sari et al. (2018) [17], which was significantly lower than healthy populations and the daily recommended intake. Similar results were also shown in Malaysia, where tuberculosis patients consumed only 922.4 kcal, as mentioned in a study by Pakasi et al. (2009) [62]. In patients with type 2 diabetes mellitus, energy intakes were also reported to be lower than in healthy populations; these findings were similar to previous studies. Energy intake was found to be associated with diabetes mellitus [111]. Among hemodialysis patients with type 2 diabetes, daily energy intake is often found to be less than required [112].

Healthy adults in Malaysia generally met the daily protein intake recommendation. A study with university students and working adults in Malaysia showed protein intake near or above the respective RNIs [34,113]. Various factors, including socioeconomic changes, cultural shifts, and increased availability of protein-rich foods, contribute to the exceeded protein intake of Malaysian adults [[44], [53]]. On the contrary, individuals with specific clinical conditions, including tuberculosis and HIV, reported protein intake below the recommended levels [26,114]. Protein deficiency in populations with chronic infections such as tuberculosis is concerning because it may weaken the immune system and contribute to other complications [115].

The comparison of fat intake among healthy populations and those with clinical conditions also highlighted notable differences. Healthy individuals, particularly in urban area, tended to have higher fat intake, as reported by Chai et al. (2019) [47] and Gan et al. (2011) [34]. Meanwhile, patients with clinical conditions such as tuberculosis showed significantly lower fat intake. This finding aligns with a previous study that indicated patients had reduced total fat consumption [94]. The low fat intake observed in clinical populations is concerning as it can exacerbate energy deficits, and hinder the absorption of fat-soluble vitamins (A, D, E, K) [116].

Indonesian and Malaysian adults are facing a severe nutritional crisis characterized by significantly low intake of fiber. This alarming trend is not unique to Southeast Asia, as a study in Mexico also found low intake of fiber across populations, coupled with excessive intake of added sugars and saturated fat. The global burden of disease study in 2017 further emphasized the gravity of the situation, identifying low intake of fiber-source foods such as whole grains and fruits as 2 of the leading causes of diet-related deaths and two-thirds of diet-related disability-adjusted life-years. A prospective study in Japan by Katagiri et al. in 2020 [117] underscored the life-threatening consequences of this deficiency, showing a significant association between fiber intake from beans, vegetables, and fruits and total mortality. Previous studies have also highlighted the importance of fiber intake in reducing the risk of chronic obstructive pulmonary disease (COPD), CVD, and coronary artery disease. Additonally, a study by Stefani et al. 2018 [37] found that poor diet quality among Indonesian women, specifically due to low consumption of fruit, vegetables, and protein, is associated with an increased risk of elevated fasting blood glucose and HbA1c.

### Fat-soluble vitamins: vitamin A, vitamin D, vitamin E, and vitamin K

Our study discovered that vitamin A intake differs between two countries, although Indonesia's intake is slightly lower. Studies in North America and Canada found insufficient intake of vitamin A (45% and 47%, respectively) in the adult population [118,119]. A study conducted by Liu et al. in 2019 [120] indicated that vitamin A intake among older adults was inadequate, and that socioeconomic status has a possible impact on their consumption. A study performed by Beydoun et al. in 2019 [121] investigating the effect of vitamin A deficiency presented that total and individual carotenoids have an inverse relationship with metabolic syndrome, although retinol did not show similar correlation. Vitamin A effect on bone health has various results. According to a previous review in 2021, dietary intake of vitamin A could increase bone density and lower the risk of fracture, although the form of supplementation could increase the risk of fracture [122].

A low vitamin D intake among Malaysian and Indonesian adults in this study was aligned with a review by Isa et al. in 2022 [123] that reported Malaysian adults from various ethnicities and ages have low levels of vitamin D. A study among Malaysian university students found that the median vitamin D intake only amounted to 1.37 μg/d (9.1% RNI) [124]. Vitamin D deficiency in adults could accelerate fractures, osteoporosis, and osteopenia [125]. In addition, an earlier study of Malaysian urban adults found that insufficient vitamin D intake could increase the risk of metabolic syndrome [126].

Vitamin E intake among Malaysian and Indonesian adults did not meet the recommended dietary intake. These results are aligns with a study from North America, which reported insufficient dietary vitamin E intake among United States adults [127]. In comparison, a study conducted by Kim et al. in 2015 [128] showed that adults in South Korea had adequate vitamin E intake. Inadequacy of vitamin E intake has been associated with health risks in adults. According to a national health survey in North America, higher vitamin E intake was associated with a decreased incidence of COPD [127]. A meta-analysis by Cheng et al. (2018) [129] also reported that a higher dietary intake of vitamin E was associated with a lower risk of stroke.

Among the retrieved studies, 1 reported that vitamin K intake of an Indonesian university's students met the recommended dietary intake. A study using national health survey data in South Korea has similar findings: vitamin K intake among adults was higher than recommended [128]. Adequate vitamin K intake positively impacts reducing risk of NCDs. Previous cohort studies found that increased vitamin K2 intake was associated with a decreased risk of coronary artery disease [130] and a lower risk of metabolic syndrome [131].

### Water-soluble vitamins: vitamin B (thiamine, riboflavin, niacin, pyridoxine, cobalamin, folate) and vitamin C

Water-soluble vitamin intake in Indonesia and Malaysia was complex, influenced by demographic factors, dietary habits, and supplementation. This variability underscored the need for tailored interventions that consider these diverse factors. In Malaysia, riboflavin intake among various groups demonstrated a wide range of values, from sportsmen engaged in ‘sepak takraw’ who reported intake from 18 to 56 mg daily, indicating potential dietary enhancements linked to their athletic activities and nutritional awareness, to Malaysian estate workers who exhibited 52 to 56 mg intake, suggesting higher nutrient consumption, possibly due to occupational energy demands. Among type 2 diabetes patients, riboflavin intake varied dramatically from 28 to 124 mg, underscoring the necessity for tailored dietary management for chronic disease conditions. University students showed significant sex-based disparities, with intake ranging from 155 to 347 mg, reflecting differences in dietary behaviors and nutritional knowledge. These findings highlighted the need for targeted interventions addressing these specific groups and their unique nutritional needs.

Urban-rural differences were also evident, with adult women in Kuala Lumpur consuming between 5 and 85 mg of riboflavin, likely reflecting varying access to nutrient-rich foods and differing dietary practices in urban environments. In rural Malaysia, newly diagnosed cancer patients had a relatively consistent riboflavin intake of 40 mg, pointing to the need for targeted nutritional support. Indonesian studies reported riboflavin intakes ranging from 10 to 503 mg daily among university students and the general population, highlighting significant variability and potential gaps in nutritional adequacy.

Thiamine consumption among adults in both countries showed more consistency but still varied. In Malaysia, male intake ranged from 0.8 to 1.39 mg, similar to the mean intake among Indonesian adults (0.75 mg). Although the meta-analysis showed insufficient intake of thiamine in both countries, these findings suggest that there is still a need to ensure adequate intake, especially in rural and low-income settings with limited dietary diversity.

Niacin intake among Malaysian males varied significantly, with mean intake ranging from 8.5 to 72.5 mg. In comparison, females had lower and more consistent intake (5.3 to 21.7 mg) than males, indicating potential differences in dietary habits and nutritional needs. In Indonesia, the consistent intake (6.9 to 8.1 mg) suggested more uniform dietary patterns but also suggests more uniform dietary patterns and generally lower intake levels than in Malaysia, which could reflected economic or cultural dietary limitations. In healthy subjects, present study found varied niacin intake in urban population, the highest intake were both in Malaysian and Indonesian at 19.6 mg (144.7% RNI/RDA) and 21.7 mg (130% RNI/RDA) [18,58]. Subjects with clinical conditions such as diabetes reported lower niacin intake [33]. This finding aligned with previous study that also reported low niacin intake in diabetic groups [132]. Low niacin intake may be due to niacin effect that could slightly increase glucose levels [133].

Pyridoxine (vitamin B6) intake among Indonesian adults varied, with university students consuming between 0.5 and 0.6 mg, which was lower than the intake among nurses who consumed vitamin B supplements (9.08 mg). This stark difference underscored the impact of supplementation on nutrient intake. Meanwhile, the general population's intake (3.31 to 4.59 mg) indicated a moderate concentration of consumption that might still require nutritional improvements.

The disparities in cobalamin (vitamin B12) intake were not just numbers, they represented a significant issue that demands immediate attention. For instance, vegetarian Malaysian females had the lowest intake at 0.51 μg, indicating significant dietary restrictions. Conversely, Indonesian nurses who took vitamin B supplements had the highest intake at 9.34 μg. Among university students, intake varied between 2.5 to 2.9 μg, which might be adequate but suggesting the importance of dietary diversity and potential supplementation, especially in vegetarian populations. Folate intake also varied widely. Malaysian vegetarian females had higher intake (192.9 μg) compared to those on a regular diet (53.6 μg), suggesting that vegetarian diets may place a greater emphasis on folate-rich foods or supplements. In Indonesia, university students had folate intake ranging from 103.7 to 123.7 μg, indicating a moderate level of consumption. Nurses who took vitamin B supplements reported higher intake (305 μg) than those who did not take supplements (229 μg), further highlighting the role of supplementation in achieving adequate nutrient intake.

The variety of vitamin B intake in Indonesia and Malaysia's adult population was similar to the current status in other Asian countries. Research in South Asia had found a high prevalence of vitamin B12 deficiency, particularly among vegetarians, with a significant difference in deficiency rates across age groups [134]. This was consistent with findings in New Zealand, where South Asian communities were identified as being at risk of compromised vitamin B12 status [135]. A study of South Asian women in New Zealand found that a dietary intake of <2.4 μg/day increased the possibility of B12 insufficiency. This study revealed a startling fact: a dietary intake of <2.4 μg/day significantly increased risk of B12 insufficiency, potentially leading to serious health complications. This underscores the importance of addressing vitamin B12 deficiency in South Asia [136]. The collective findings from these studies underscored the urgent need for further research and public health interventions to address vitamin B12 deficiency in South Asia. Despite ongoing efforts to improved vitamin B intake through dietary changes and supplementation, the region still faces significant hurdles. Ensuring adequate intake of B vitamins among adults required a multifaceted approach, including better dietary education, improved food security, and access to fortified foods and supplements.

Our study showed the most variation regarding vitamin C intake. In vegetarian subjects, it was found that they have a high vitamin C intake, 190.8 mg [47]. This aligned with a previous study that also reported a high vitamin C intake in individuals who followed a vegetarian diet compared to non-vegetarian individuals [137,138]. This could be due to the high availability of dietary vitamin C in fruits and vegetables [139]. In Malaysia, female intake ranged from 25 to 190.8 mg and male intake from 26.84 to 100 mg, reflecting diverse dietary patterns. Indonesian adults had intake ranging from 32.9 to 152.3 mg, with university students consuming 29.2 (male) and 37.5 mg (female). Similarly, in South Asia countries, particularly in Pakistan and India, there was a significant decrease in vitamin C levels, with a high prevalence of deficiency [140]. In China, the dietary intake of vitamin C was inadequate, with a high risk of insufficiency [141]. Likewise, a high prevalence of vitamin C deficiency was reported in India, particularly in older adults and those with poor nutrition [142]. The variations suggest differences in fruit and vegetable consumption and point to potential areas for dietary improvement. Although there are concerted efforts across Southeast Asia to improve vitamin C intake, the results vary significantly. Economic factors, dietary habits, and access to fresh produce play crucial roles in determining the vitamin C status of the populations [143]. Like several other countries in the region, Indonesia and Malaysia face significant challenges but are actively working toward improving the situation through various public health strategies. Addressing these challenges requires a multifaceted approach involving education, improved food distribution systems, and ongoing public health campaigns to ensure that all populations can meet their nutritional needs.

### Macro minerals: calcium, phosphorus, magnesium, sodium, potassium, and chloride

Inadequate calcium intake, as seen in Indonesian and Malaysian adults, is also prevalent in several other Southeast Asian countries, including Thailand, Vietnam, and the Philippines. Regardless of various characteristics, such as areas and gender, the daily calcium intake in these 3 countries is below recommendation [[144], [145], [146]]. A study by Neufingerl et al. in 2022 [147] examined the risk of inadequate calcium intake among vegetarians, vegans, and meat-eaters in Europe, South Asia, or North Asia, with mean intake of around 800 mg/d. Adequate calcium consumption in adults can lead to positive health outcomes, including reduced risk of high blood pressure, lower low-density lipoprotein cholesterol, and prevention of osteoporosis and colorectal cancer [148]. According to Bourassa et al. in 2022 [149], policy interventions are crucial to increase calcium intake, not only through animal sources with high bioavailability of calcium and plant foods but also through fortification of staple foods with calcium and changes in food processing techniques.

Our study discovered that adults in Indonesia and Malaysia have ≥90% EAR of phosphorus intake. This finding indicated that most adults in these countries meet the minimum phosphorus intake (700-1250 mg) necessary for good health. In a study conducted on Indonesian adults, Khusun et al. (2023) [30] suggested that regular breakfast consumption is linked to higher phosphorus intake. Another study noted the importance of considering weight status when assessing phosphorus intake, as body weight increased, the impact of phosphorus intake on individuals decreased in mg/kg. These findings highlight the need for dietary recommendations tailored to adults in Indonesia and Malaysia, considering individual factors such as meal patterns and body weight [150]. The magnesium intake in Indonesia and Malaysia varied, with a minimum of 30% EAR. Comparatively, a study in South Korea found that magnesium intake of Korean exceeded 100% EAR [151]. A study by Wei et al. in 2016 [152] aimed to examined a correlation between magnesium intake and diabetes occurrence. However, the results indicated no significant correlation.

This study found that Indonesian and Malaysian adults had sodium intake of >85% EAR. This high sodium intake is concerning because it is associated with elevated blood pressure and impaired functioning of several organs, including blood vessels, the heart, kidneys, and brain regions that control autonomic function [153]. In addition, the study by Md Yusop et al. (2013) [36] showed that hemodialysis patients also had high sodium intake, which is particularly problematic since they should limit their sodium intake due to its major role in adverse health outcomes [154].

A broader study conducted in Southeast Asia, which included adult populations in 5 countries (Indonesia, Malaysia, Singapore, Philippines, and Thailand) demonstrated that sodium intake surpassed recommendations (>2 g/d). These findings emphasized the urgent need for public health interventions aimed at reducing sodium intake. Such interventions included educating the public about the health risks associated with high-sodium diets and promoting low-sodium food options [155]. The role of public health interventions cannot be overstated in addressing these nutritional challenges. A meta-analysis outlined that global potassium intake also fell short of recommendations, with Asia having the lowest potassium intake compared to Western and Eastern Europe which had the highest intake [156]. Similarly, the potassium intake of Malaysians and Indonesians is below the recommendations outline in this review. Aburto et al. (2013) [157] examined that optimal potassium intake of 90 to 120 mmol/d might help reduce blood pressure and lower the risk of stroke. However, intake above 120 mmol/d did not provide additional benefits.

### Trace elements: iron, manganese, copper, iodine, zinc, cobalt, fluoride, and selenium

Indonesia and Malaysia have been found to have a significant prevalence of iron deficiency among their adult populations, which is commonly associated with anemia. A previous study highlighted the strong correlation between the prevalence of anemia in adult Indonesian women and the lack of egg consumption (iron source food) [158]. In Malaysia, it was reported that half of the anemia cases in adult women, which accounted for 20% of the population were attributed to iron deficiency. Among different ethnicities, the Indian population had the highest prevalence of iron deficiency anemia. This is primarily due to their preference for legumes (dhal), which contained low levels of iron bioavailability due to their phytate content, that inhibits iron absorption. Another study supported these findings, noting that iron deficiency anemia is more common in women of reproductive age due to blood loss during menstruation. Additionally, adults with vegetarian diets were reported to have low iron intake [47]. This result is in contrast with previous studies, which indicated that vegetarians had met the recommended iron intake. The low level of iron intake on vegetarian could be due to lower bioavailibility of non-heme iron in plant-based foods [137,159]. It is important to note that a lack of iron intake can lead to various health issues, such as fatigue and restless legs. These findings emphasize the urgent need to address iron deficiency in these populations to improve their health outcomes.

Zinc intake in Indonesian and Malaysian adults did not meet 100% EAR. Similarly, research on Thai adults depicted comparable results (<80% Thai Dietary Reference Intakes or DRIs) [98]. In Vietnam, a study discovered that 85% of women of reproductive age had zinc deficiency, with 35% experiencing severe deficiency [160]. As an enzyme cofactor in metabolic processes, the role of zinc is critical in the pathophysiology of diseases such as cancer, diabetes, and obesity. Inadequate zinc intake can increase oxidative damage to the body, contributing to cell damage in chronic diseases [161]. Furthermore, a study by Pakasi et al. (2009) [62] showed that tuberculosis patients tended to have a lower zinc intake, as micronutrient deficiencies are common in individuals with this condition [162]. This finding was consistent with a trial that found plasma zinc concentrations were not significantly higher in the micronutrient group compared to the placebo group after 6 mo of intervention [163]. Additionally, a zinc-rich diet had been shown to reduces the risk of depression, type 2 diabetes, and cancers of the digestive system in adults. A recommendation is to consume an extra 5 mg zinc daily to lower the risk of esophageal and colorectal cancers [164].

Healthy adults in Malaysia had a significant deficiency in manganese intake, at around 0.4 mg per day [15]. In contrast, healthy adults in Indonesia consumed between 1.6 to 2.2 mg manganese per day [29]. This amount is considerably lower than that of Japanese adults who had intake of 5.1 mg/d for men and 4.9 for women. In Japan, white rice contributes to 20% – 30% of the total manganese intake among adults [165]. Similarly, rice served as a primary source of manganese intake in both Indonesia and Malaysia given its status as a staple food [166,167]. Fruits and legumes such as tempeh and tofu can provide good alternatives for manganese food sources [166]. However, the lack of research on manganese intake in Indonesia and Malaysia highlights the necessity for further study. Adequate consumption of manganese is essential for maintaining bone health, particularly in preventing bone loss through the synthesis and mineralization of cartilage and bone collagen. This makes manganese intake is a crucial area for future research [168].

Copper intake in both countries was below 100% of the recommended EAR. Insufficient copper intake is associated with neurodegenerative diseases, including Alzheimer, Parkinson, Wilson's disease, and Menkes disease [169]. Likewise, excessive copper consumption can increase the risk of atherosclerotic CVD [170]. In term of selenium intake, Indonesia has met the recommended levels, while Malaysia has wider range of intake level. A previous study found a reduced risk of hypertension with adequate daily intake of selenium-source foods [86]. Additionally, sufficient selenium consumption can contribute to thyroid hormone synthesis and help maintain thyroid function [171]. Rice and paddy are good source of selenium. However, the amount of selenium content in these foods can be influenced by the soil conditions. Therefore, in selenium-deficient environments, these foods are likely to contained less selenium increasing the risk of selenium deficiency [144,145].

### Strengths and limitations of the study

It was stimulating to draw the percentage adequacy of each nutrient particularly due to the overlap in age categories between dietary intake recommendations in Indonesia and Malaysia with the age group used in the studies. Moreover, many studies combined the data between male and female adult populations. This is the first comprehensive review evaluating macro- and micronutrient intake among Indonesian and Malaysian adults. Eventually, we included all relevant studies involving Indonesian and Malaysian populations, regardless the sample size or specific health conditions, such as diabetis or kidney diseases, while assessing the potential for moderate or high risk of bias in the studies. The current review found a limited studies for specific nutrient intake among Indonesian and Malaysian adults, especially micronutrients such as vitamin K, iodine, cobalt, and fluoride. Further investigation is neccessary, as previous studies demonstrated the imbalances in nutrient intake can lead to brain damage. This includes impairments in areas responsible for autonomic control, as well as conditions such as Alzheimer's disease, mild cognitive impairment, and dementia [97,98,89]. These findings emphasize the importance of gathering substantial evidence on the relationship between macronutrient and micronutrient intake, diet quality, brain damage, and NCDs among adult populations in Indonesia and Malaysia.

## Conclusion

This comprehensive review revealed variations in energy and macronutrient intake among Indonesian and Malaysian adults. Malaysian had a higher intake of macronutrients than Indonesian adults. Potein intake exceeded the recommendation among Malaysian but was insufficient among Indonesian adults. Low intake of fiber, fat-soluble vitamins (except for vitamin A), calcium, potassium, manganese, and copper persisted in both countries. Indonesian lacked iron and zinc intake, while Malaysian had low magnesium intake. In contrast, excessive sodium and phosphorus intake had been observed in Malaysian, while Indonesians showed excessive selenium intake.

Therefore, there is a need for a well-built multistakeholder collaboration to create nutrient sensitive and specific programs aiming for a sufficient and high-quality dietary intake among this population. Two key recommendations for enhancing nutrition interventions are the regulation and implementation of food fortification using high bioavailability food sources and advanced food processing technologies to minimize nutrient loss. Dietary diversification should be promoted at the individual-level through government regulation, such as updating dietary guidelines based on local food sources, supporting farmers in enhancing the production of local plants and, and maintaining the price floors and ceilings for local foods to ensure food accessibility. National-level surveys should be conducted regularly with appropriate dietary assessment to monitor the nutrient intake, involving academic experts to evaluate the effectiveness and stipulate the dietary guidelines in response to the recent nutrient intake condition.

## Author contributions

The authors‘ responsibilities were as follows – RA, RM: conceptualization; RA, RM, DASS: methodology and validation; RM, WL, DASS: software, visualization, and project administration; RM, AM, AD, DASS, WL: formal analysis; RA, RM, WL, AM, AD, DASS, STL: writing—original draft preparation; RA, EP, NRMM, PS: writing—review and editing, supervision, and funding acquisition; and all authors: read and approved the final manuscript.

## Funding

This research was funded by Blackmores Institute (Research and Education Division of Blackmores Limited) through Pusat Pengembangan Kedokteran Indonesia Fakultas Kedokteran Universitas Indonesia (PUSBANGKI FKUI), the Ministry of Research through Basic Research grant number NKB-018/UN2.RST/HKP.05.00/2021 (Ristekdikti-Penelitian Dasar), and Universitas Indonesia through PUTI Q1 DRPM UI 2022 to RA (Number: NKB-432/UN2.RST/HKP.05.00/2022).

## Conflict of interest

The authors report no conflicts of interest.
