# Supplementary material for: Nutrient Intake Adequacy among Adults in Indonesia and Malaysia: A Systematic Review and Meta-Analysis
Source: Curr Dev Nutr. 2025 Mar 24;9(5):106010. doi: 10.1016/j.cdnut.2025.106010 (PMC12142331; doi:10.1016/j.cdnut.2025.106010)
Supplement: Multimedia component 1 [file mmc1.docx]

**Nutrient intake adequacy among adults in Indonesia and Malaysia: a systematic review and meta-analysis**

First Author: Rina Agustina^1,2,3^

^1^Department of Nutrition, Faculty of Medicine, Universitas Indonesia, Dr. Cipto Mangunkusumo General Hospital, Jakarta, Indonesia

^2^Human Nutrition Research Center, Indonesian Medical Education and Research Institute, Faculty of Medicine, Universitas Indonesia, Jakarta, Indonesia

Supplementary Table 1. Risk of Bias

The following criteria were applied to evaluate bias in the studies included:

- **Selection Bias**: This was assessed based on the sampling method. A score of 1 was given for random sampling, while purposive or convenience sampling received a score of 2. Additionally, a score of 1 was assigned if the sample represented adults across all age groups, whereas studies focusing on specific populations were scored as 2.
- **Performance Bias**: This was evaluated according to the dietary assessment method used. Scoring was as follows: food weighing (1), multiple 24-hour recalls (2), food diaries over several days (3), diet history (4), a single 24-hour recall (5), and food frequency questionnaires (FFQs) assessing usual consumption (6). Furthermore, studies were scored 1 if they measured usual intake and 2 if they did not.
- **Reporting Bias**: This was determined using two factors. A score of 1 was given if studies excluded over- or under-reporters, and 2 if they included them. Similarly, a score of 1 was assigned if all available databases were utilized, while studies using only free-access databases scored 2.

The total bias risk score was categorized as follows: low risk of bias (6–9), moderate risk (10–13), and high risk (14–16).

| **Author, Year** | **Title of the study** | **Selection bias** | | **Performance bias** | | **Reporting bias** | | **Total score** | **Classification of risk of bias** |
| --- | --- | --- | --- | --- | --- | --- | --- | --- | --- |
|  |  | **Sampling method** | **Representative of age** | **Dietary assessment method used** | **Usual intake measured** | **considering under/over reported** | **Primary data collection vs paid database** |  |  |
| Angeles-Agdepa, 2019^49^ | Usual energy and nutrient intakes and food sources of Filipino children aged 6-12 years from the 2013 national nutrition survey | 1 | 1 | 2 | 1 | 2 | 1 | 8 | Low Risk |
| Angelin, 2021^45^ | Growth, dietary intake, and vitamin D receptor (VDR) promoter genotype in Indonesia school-age children | 2 | 1 | 2 | 2 | 1 | 1 | 9 | Low Risk |
| Boonchoo, 2017^53^ | Dietary intake and weight status Thai preadolescents in the context of food environment | 2 | 2 | 1 | 2 | 1 | 1 | 9 | Low Risk |
| Chee, 2021^34^ | Vitamin D status is associated with modifiable lifestyle factors in pre-adolescent childen living in urban Kuala Lumpur, Malaysia | 2 | 2 | 4 | 2 | 2 | 1 | 13 | Moderate Risk |
| Citrakesumasari, 2020^44^ | Analysis of nutrition intake based on gender in adolescents | 1 | 2 | 5 | 2 | 2 | 1 | 13 | Moderate Risk |
| Ekawati, 2021^46^ | Evaluation of dietary antioxidant intake of school-age children in gold mining area Sekotong,, West Lombok, Indonesia | 2 | 2 | 5 | 2 | 2 | 1 | 14 | High Risk |
| Elias, et al., 2007^30^ | Relationship between blood lead concentration and nutrtuional status among malay primary school children in Kuala Lumpur, Malaysia | 1 | 2 | 2 | 1 | 2 | 1 | 9 | Low Risk |
| Golloso-Gubat, 2020^50^ | Gut microbiota and dietary intake of normal-weight and overweight Filipino children | 2 | 2 | 6 | 1 | 2 | 1 | 14 | High Risk |
| Kuralneethi, et al., 2021^35^ | Association between dietary quality and growth of the aboriginal primary school children in Negeri Sembilan | 2 | 2 | 2 | 2 | 2 | 1 | 11 | Moderate Risk |
| Kurniawan, et al., 2006^40^ | Anaemia and iron deficiency anaemia among young adolescent girls from peri urban coastal area of Indonesia | 2 | 2 | 5 | 2 | 2 | 1 | 14 | High Risk |
| Meli, 2023^28^ | Risk assessment of low cognitive performance among fishermen's children in Malaysia | 2 | 2 | 2 | 1 | 2 | 1 | 10 | Moderate Risk |
| Ai Kah Ng, et al., 2019^33^ | Dietary inatke, physical activity and muscle strength among adolescents: the Malaysian Health and adolescents longitudinal research team (MyHeART) study | 1 | 2 | 3 | 2 | 2 | 2 | 12 | Moderate Risk |
| Nguyen, 2013^54^ | Double burden of undernutrition and overnutrition in Vietnam in 2011: results of SEANUTS study 0,5 - 11-year-old children | 1 | 1 | 5 | 2 | 2 | 1 | 12 | Moderate Risk |
| Norhayati, et al., 1995^27^ | Nutrient intake and socio-economic status among children attending a health exhibition in Malaysian rural villages | 2 | 2 | 2 | 2 | 2 | 1 | 11 | Moderate Risk |
| Palaniveloo, et al., 2021^36^ | Associations of Gender and BMI-for-age status (BAZ) with nutrient intake among adolescents in Malaysia: findings from adolescent nutrition survey (ANS) 2017 | 1 | 1 | 5 | 2 | 1 | 1 | 11 | Moderate Risk |
| Rahfiludin, et al., 2021^47^ | Plant-based diet and iron deficiancy anemia in Sundanese adolescent girls at Islamic boarding schools in Indonesia | 1 | 1 | 5 | 2 | 2 | 1 | 12 | Moderate Risk |
| Rojroongwasinkul, et al., 2013^52^ | SEANUTS: the nutritional status and dietary intakes of 0.5-12years-old Thai children | 1 | 1 | 5 | 1 | 2 | 1 | 11 | Moderate Risk |
| Sekiyama, et al., 2017^43^ | Locally sustainable school lunch intervention improves hemoglobin and hematocrit levels and body mass index among elementary schoolchidlren in Rural West Java, Indonesia | 2 | 2 | 5 | 2 | 2 | 1 | 14 | High Risk |
| Sallehuddin, et al., 2021^37^ | Calcium and iron intakes of adolescents in Malaysia and their relationships with body mass index (BMI): Findings from adolescent nutrition survey 2017 | 1 | 1 | 5 | 2 | 2 | 1 | 12 | Moderate Risk |
| Sandjaja, 2013^41^ | Food consumption and nutritional bichemical status of 0.5-12 years old Indonesian children: the SEANUTS study | 2 | 1 | 5 | 2 | 2 | 1 | 13 | Moderate Risk |
| Sari, et al., 2022^48^ | Iron deficiency anemia and associated factors among adolescent girls and women in a rural area of Jatinangor, Indonesia | 1 | 2 | 5 | 2 | 2 | 1 | 13 | Moderate Risk |
| Setiawan, et al., 1999^39^ | Vitamin B-6 inadequacy is prevalent in rural and urban Indonesia children | 1 | 2 | 2 | 1 | 2 | 1 | 9 | Low Risk |
| Suriawati, et al., 2016^31^ | Vitamin D and Calcium intakes, physica; activity, and calcaneus BMC among school-going 13-year-old malaysian adolescents | 1 | 2 | 4 | 2 | 1 | 1 | 11 | Moderate Risk |
| Susanto, et al., 2015^42^ | Nutrient intake evaluation among school age children in Jakarta, Indonesia | 1 | 1 | 5 | 2 | 2 | 2 | 13 | Moderate Risk |
| Tay, et al., 2023^38^ | Food security and diet quality among urban poor adolescents in Kuala Lumpur, Malaysia | 1 | 1 | 2 | 2 | 2 | 1 | 9 | Low Risk |
| Wei, et al., 2018^32^ | Breakfast consumption and its relationship with cognitive performance among fishermen's children in terengganu | 1 | 1 | 2 | 2 | 2 | 1 | 9 | Low Risk |
| Zalilah M S, et al., 2006^29^ | Dietary intake, physical activity, and energy expenditure of Malaysian adolescents | 1 | 2 | 3 | 2 | 2 | 1 | 11 | Moderate Risk |

**Supplementary Table 2. Micronutrients among Children and Adolescent in Five Countries**

| **Author (Year)** | **Study Design** | **Sample size (n)** | **Subject characteristics (age, gender, area/characteristics)** | **Dietary assessment method, source of nutrient** | **Nutrient identified** | **Intake (%RDA/%RNI/%EAR)** | **Nutrient (food source identified)** |
| --- | --- | --- | --- | --- | --- | --- | --- |
| **Indonesia** | | | | | | | |
| Setiawan et al. (1999) | Cross-sectional | 18 | 8-9/female/rural | 24-hour recall questionnaire, food | Vitamin B6 | 0.4 mg (40) |  |
| Setiawan et al. (1999) | Cross-sectional | 20 | 8-9/male/rural | 24-hour recall questionnaire, food | Vitamin B6 | 0.52 mg (52) |  |
| Setiawan et al. (1999) | Cross-sectional | 38 | 8-9/male and female/rural | 24-hour recall questionnaire, food | Vitamin B6 | 0.46 mg (46) |  |
| Setiawan et al. (1999) | Cross-sectional | 20 | 8-9/female/urban | 24-hour recall questionnaire, food | Vitamin B6 | 0.64 mg (64) |  |
| Setiawan et al. (1999) | Cross-sectional | 19 | 8-9/male/urban | 24-hour recall questionnaire, food | Vitamin B6 | 0.7 mg (70) |  |
| Setiawan et al. (1999) | Cross-sectional | 39 | 8-9/male and female/urban | 24-hour recall questionnaire, food | Vitamin B6 | 0.67 mg (67) |  |
| Setiawan et al. (1999) | Cross-sectional | 38 | 8-9/female | 24-hour recall questionnaire, food | Vitamin B6 | 0.53 mg (53) |  |
| Setiawan et al. (1999) | Cross-sectional | 39 | 8-9/male | 24-hour recall questionnaire, food | Vitamin B6 | 0.62 mg (62) |  |
| Kurniawan et al. (2006) | Cross-sectional | 104 | 10-12/female | 24-hour food recall and FFQ, food | Vitamin C | 33 mg (66) |  |
| Sandjaja et al. (2013) | Cross-sectional | 682 | 5-12/male/urban | 24-hour recall questionnaire; food | Vitamin C | 18 mg (38.5) |  |
| Sandjaja et al. (2013) | Cross-sectional | 670 | 5-12/female/urban | 24-hour recall questionnaire; food | Vitamin C | 16 mg (34.3) |  |
| Sandjaja et al. (2013) | Cross-sectional | 729 | 5-12/male/rural | 24-hour recall questionnaire; food | Vitamin C | 14 mg (30) |  |
| Sandjaja et al. (2013) | Cross-sectional | 729 | 5-12/female/rural | 24-hour recall questionnaire; food | Vitamin C | 14 mg (30) |  |
| Susanto et al. (2015) | Cross-sectional | 945 | 6-12/male and female | 24-hr dietary recall, food | Vitamin B6 | 26.99 mg (2249.2) |  |
| Susanto et al. (2015) | Cross-sectional | 945 | 6-12/male and female | 24-hr dietary recall, food | Vitamin C | 425.75 mg (881.5) |  |
| Susanto et al. (2015) | Cross-sectional | 137 | 6-12/male and female/Low SES | 24-hr dietary recall, food | Vitamin B6 | 0.59 mg (49.2) |  |
| Susanto et al. (2015) | Cross-sectional | 137 | 6-12/male and female/Low SES | 24-hr dietary recall, food | Vitamin C | 17.4 mg (36) |  |
| Susanto et al. (2015) | Cross-sectional | 198 | 6-12/male and female/medium SES | 24-hr dietary recall, food | Vitamin B6 | 0.75 mg (62.5) |  |
| Susanto et al. (2015) | Cross-sectional | 198 | 6-12/male and female/medium SES | 24-hr dietary recall, food | Vitamin C | 0.75 mg (1.6) |  |
| Susanto et al. (2015) | Cross-sectional | 610 | 6-12/male and female/high SES | 24-hr dietary recall, food | Vitamin B6 | 0.77 mg (64.2) |  |
| Susanto et al. (2015) | Cross-sectional | 610 | 6-12/male and female/high SES | 24-hr dietary recall, food | Vitamin C | 30.9 mg (64) |  |
| Citrakesumasari et al. (2020) | Cross-sectional | 72 | 15-17/female | 24-hour recall, food and supplement | Vitamin B6 | 0.87 mg (72.5) |  |
| Citrakesumasari et al. (2020) | Cross-sectional | 72 | 15-17/female | 24-hour recall, food and supplement | Vitamin B9 | 94.48 mcg (23.6) |  |
| Citrakesumasari et al. (2020) | Cross-sectional | 72 | 15-17/female | 24-hour recall, food and supplement | Vitamin C | 19.74 mg (26.3) |  |
| Ekawanti et al. (2021) | Cross-sectional | 21 | 10-12/male | 24-hour recall, food and supplement | Vitamin E | 1.17 mcg (10.6) |  |
| Ekawanti et al. (2021) | Cross-sectional | 21 | 10-12/male | 24-hour recall, food and supplement | Vitamin C | 61.85 mg (123.7) |  |
| Ekawanti et al. (2021) | Cross-sectional | 21 | 10-12/male | 24-hour recall, food and supplement | Copper | 3.87 mcg (0.6) |  |
| Ekawanti et al. (2021) | Cross-sectional | 21 | 10-12/male | 24-hour recall, food and supplement | Mangan | 2.25 mg (118.4) |  |
| Ekawanti et al. (2021) | Cross-sectional | 50 | 7-9/female | 24-hour recall, food and supplement | Vitamin E | 1.47 mcg (18.4) |  |
| Ekawanti et al. (2021) | Cross-sectional | 50 | 7-9/female | 24-hour recall, food and supplement | Vitamin C | 6.33 mg (14.1) |  |
| Ekawanti et al. (2021) | Cross-sectional | 50 | 7-9/female | 24-hour recall, food and supplement | Copper | 0.24 mcg (0.04) |  |
| Ekawanti et al. (2021) | Cross-sectional | 50 | 7-9/female | 24-hour recall, food and supplement | Mangan | 1.1 mg (64.7) |  |
| Ekawanti et al. (2021) | Cross-sectional | 50 | 10-12/female | 24-hour recall, food and supplement | Vitamin E | 2.14 mcg (14.27) |  |
| Ekawanti et al. (2021) | Cross-sectional | 50 | 10-12/female | 24-hour recall, food and supplement | Vitamin C | 27.08 mg (54.16) |  |
| Ekawanti et al. (2021) | Cross-sectional | 50 | 10-12/female | 24-hour recall, food and supplement | Copper | 0.37 mcg (0.05) |  |
| Ekawanti et al. (2021) | Cross-sectional | 50 | 10-12/female | 24-hour recall, food and supplement | Mangan | 1.87 mg (116.88) |  |
| Ekawanti et al. (2021) | Cross-sectional | 50 | 13-15/female | 24-hour recall, food and supplement | Vitamin E | 1.8 mcg (12.0) |  |
| Ekawanti et al. (2021) | Cross-sectional | 50 | 13-15/female | 24-hour recall, food and supplement | Vitamin C | 31.6 mg (48.6) |  |
| Ekawanti et al. (2021) | Cross-sectional | 50 | 13-15/female | 24-hour recall, food and supplement | Copper | 0.4 mcg (0.1) |  |
| Ekawanti et al. (2021) | Cross-sectional | 50 | 13-15/female | 24-hour recall, food and supplement | Mangan | 3.2 mg (200.0) |  |
| Rahfiludin et al. (2021) | Cross-sectional | 176 | 15.2/female | 24-hour dietary recall, food | Vitamin C | 6.34 mg (9.8) |  |
| Sari et al. (2022) | Cross-sectional | 95 | 10-19/female | 24-hour recall questionnaire, food | Vitamin C | 41.09 mg (65.2) |  |
| **Malaysia** | | | | | | | |
| Norhayati et al. (1995) | Cross-sectional | 31 | 6/Boys and girls | One-week food recall, food | Thiamine | 0.5 mg (83.33) |  |
| Norhayati et al. (1995) | Cross-sectional | 31 | 6/Boys and girls | One-week food recall, food | Niacin | 4.9 mg (61.25) |  |
| Norhayati et al. (1995) | Cross-sectional | 31 | 6/Boys and girls | One-week food recall, food | Ascorbic acid | 26.9 mg (89.67) |  |
| Norhayati et al. (1995) | Cross-sectional | 21 | 7/Boys and girls | One-week food recall, food | Thiamine | 0.6 mg (66.67) |  |
| Norhayati et al. (1995) | Cross-sectional | 21 | 7/Boys and girls | One-week food recall, food | Niacin | 5.4 mg (45) |  |
| Norhayati et al. (1995) | Cross-sectional | 21 | 7/Boys and girls | One-week food recall, food | Ascorbic acid | 45.5 mg (130) |  |
| Meli et al. (2003) | Cross-sectional | 54 | 7-9/boys | Two 24-hour dietary recall surveys, food | Thiamine | 0.26 mg (28.89) |  |
| Meli et al. (2003) | Cross-sectional | 54 | 7-9/boys | Two 24-hour dietary recall surveys, food | Niacin | 8.08 mg (67.33) |  |
| Meli et al. (2003) | Cross-sectional | 54 | 7-9/boys | Two 24-hour dietary recall surveys, food | Vitamin B6 | 0.65 mg (65) |  |
| Meli et al. (2003) | Cross-sectional | 54 | 7-9/boys | Two 24-hour dietary recall surveys, food | Vitamin C | 27.07 mg (77.34) |  |
| Meli et al. (2003) | Cross-sectional | 54 | 7-9/boys | Two 24-hour dietary recall surveys, food | Vitamin E | 2.2 mg (31.43) |  |
| Meli et al. (2003) | Cross-sectional | 54 | 7-9/girls | Two 24-hour dietary recall surveys, food | Thiamine | 0.34 mg (37.78) |  |
| Meli et al. (2003) | Cross-sectional | 54 | 7-9/girls | Two 24-hour dietary recall surveys, food | Niacin | 9.97 mg (83.08) |  |
| Meli et al. (2003) | Cross-sectional | 54 | 7-9/girls | Two 24-hour dietary recall surveys, food | Vitamin B6 | 0.79 mg (79) |  |
| Meli et al. (2003) | Cross-sectional | 54 | 7-9/girls | Two 24-hour dietary recall surveys, food | Vitamin C | 19.58 mg (55.94) |  |
| Meli et al. (2003) | Cross-sectional | 54 | 7-9/girls | Two 24-hour dietary recall surveys, food | Vitamin E | 3.05 mg (43.57) |  |
| Meli et al. (2003) | Cross-sectional | 40 | 10-11/Boys | Two 24-hour dietary recall surveys, food | Thiamine | 0.34 mg (28.33) |  |
| Meli et al. (2003) | Cross-sectional | 40 | 10-11/Boys | Two 24-hour dietary recall surveys, food | Niacin | 10.96 mg (68.50) |  |
| Meli et al. (2003) | Cross-sectional | 40 | 10-11/Boys | Two 24-hour dietary recall surveys, food | Vitamin B6 | 0.9 mg (69.23) |  |
| Meli et al. (2003) | Cross-sectional | 40 | 10-11/Boys | Two 24-hour dietary recall surveys, food | Vitamin C | 27.93 mg (42.97) |  |
| Meli et al. (2003) | Cross-sectional | 40 | 10-11/Boys | Two 24-hour dietary recall surveys, food | Vitamin E | 3.48 mg (34.80) |  |
| Meli et al. (2003) | Cross-sectional | 40 | 10-11/girls | Two 24-hour dietary recall surveys, food | Thiamine | 0.3 mg (27.27) |  |
| Meli et al. (2003) | Cross-sectional | 40 | 10-11/girls | Two 24-hour dietary recall surveys, food | Niacin | 9.67 mg (60.44) |  |
| Meli et al. (2003) | Cross-sectional | 40 | 10-11/girls | Two 24-hour dietary recall surveys, food | Vitamin B6 | 0.76 mg (63.33) |  |
| Meli et al. (2003) | Cross-sectional | 40 | 10-11/girls | Two 24-hour dietary recall surveys, food | Vitamin C | 29.39 mg (45.22) |  |
| Meli et al. (2003) | Cross-sectional | 40 | 10-11/girls | Two 24-hour dietary recall surveys, food | Vitamin E | 2.86 mg (38.13) |  |
| Wei et al. (2018) | Cross-sectional | 48 | 7-9/male and female/fishermen's children | 3-days dietary recall, food | Vitamin C | 31.83 mg (90.9) |  |
| Wei et al. (2018) | Cross-sectional | 48 | 7-9/male and female/fishermen's children | 3-days dietary recall, food | Vitamin B9 | 14.19 mg (4.7) |  |
| Wei et al. (2018) | Cross-sectional | 52 | 10-12/male and female/fisherman's child | 3-days dietary recall, food | Vitamin C | 24.99 mg (38.4) |  |
| Wei et al. (2018) | Cross-sectional | 52 | 10-12/male and female/fisherman's child | 3-days dietary recall, food | Vitamin B9 | 14.9 mg (3.7) |  |
| Wei et al. (2018) | Cross-sectional | 35 | 7-8/male and female/fisherman's child | 3-days dietary recall, food | Copper | 230 mg (52.3) |  |
| Wei et al. (2018) | Cross-sectional | 65 | 9-12/male and female/fisherman's child | 3-days dietary recall, food | Copper | 230 mg (40.4) |  |
| Kuralneethi et al. (2021) | Cross-sectional | 97 | 7-9/male and female | 3-days dietary recall, food | Niacin | 13.13 mg (93.8) |  |
| Kuralneethi et al. (2021) | Cross-sectional | 97 | 7-9/male and female | 3-days dietary recall, food | Vitamin C | 53.4 mg (106.8) |  |
| Kuralneethi et al. (2021) | Cross-sectional | 97 | 10-12/male and female | 3-days dietary recall, food | Niacin | 16.68 mg (119.1) |  |
| Kuralneethi et al. (2021) | Cross-sectional | 97 | 10-12/male and female | 3-days dietary recall, food | Vitamin C | 39.55 mg (79.1) |  |
| Palaniveloo et al. (2021) | Cross-sectional | 449 | 13-17/Boys | 24-hours dietary recall, food | Thiamin | 0.9 mg (75) |  |
| Palaniveloo et al. (2021) | Cross-sectional | 449 | 13-17/Boys | 24-hours dietary recall, food | Vitamin C | 60.7 mg (93.4) |  |
| Palaniveloo et al. (2021) | Cross-sectional | 449 | 13-17/Boys | 24-hours dietary recall, food | Vitamin E | 5.8 mg (58) |  |
| Palaniveloo et al. (2021) | Cross-sectional | 449 | 13-17/Boys | 24-hours dietary recall, food | Sodium | 3371.7 mg (224.8) |  |
| Palaniveloo et al. (2021) | Cross-sectional | 449 | 13-17/Boys | 24-hours dietary recall, food | Potassium | 1416.1 mg (30.1) |  |
| Palaniveloo et al. (2021) | Cross-sectional | 550 | 13-17/Girls | 24-hours dietary recall, food | Thiamin | 0.8 mg (72.7) |  |
| Palaniveloo et al. (2021) | Cross-sectional | 550 | 13-17/Girls | 24-hours dietary recall, food | Vitamin C | 85.3 mg (131.2) |  |
| Palaniveloo et al. (2021) | Cross-sectional | 550 | 13-17/Girls | 24-hours dietary recall, food | Vitamin E | 11.2 mg (149.3) |  |
| Palaniveloo et al. (2021) | Cross-sectional | 550 | 13-17/Girls | 24-hours dietary recall, food | Sodium | 2602.5 mg (173.5) |  |
| Palaniveloo et al. (2021) | Cross-sectional | 550 | 13-17/Girls | 24-hours dietary recall, food | Potassium | 1170.8 mg (24.9) |  |
| Tay et al. (2022) | Cross-sectional | 188 | 13-18/male and female/urban poor | 2-day 24-h dietary recall, food | Thiamine | 1.02 mg (88.7) |  |
| Tay et al. (2022) | Cross-sectional | 188 | 13-18/male and female/urban poor | 2-day 24-h dietary recall, food | Niacin | 15.56 mg (97.3) |  |
| Tay et al. (2022) | Cross-sectional | 188 | 13-18/male and female/urban poor | 2-day 24-h dietary recall, food | Vitamin C | 56.71 mg (87.2) |  |
| **Philippines** | | | | | | | |
| Angeles-Agdeppa et al. (2017) | Cross-sectional | 6565 | 6-12/male and female | Two nonconsecutive-day 24-h dietary recalls, food | Thiamine | 0.8 mg (106.7) |  |
| Angeles-Agdeppa et al. (2017) | Cross-sectional | 6565 | 6-12/male and female | Two nonconsecutive-day 24-h dietary recalls, food | Niacin | 15.4 mg (162.1) |  |
| Angeles-Agdeppa et al. (2017) | Cross-sectional | 6565 | 6-12/male and female | Two nonconsecutive-day 24-h dietary recalls, food | Vitamin C | 23.8 mg (69.0) |  |
| Angeles-Agdeppa et al. (2017) | Cross-sectional | 6565 | 6-12/male and female | Two nonconsecutive-day 24-h dietary recalls, food | Vitamin B6 | 1.4 mg (155.6) |  |
| Angeles-Agdeppa et al. (2017) | Cross-sectional | 6565 | 6-12/male and female | Two nonconsecutive-day 24-h dietary recalls, food | Vitamin E | 2.6 mg (32.5) |  |
| Angeles-Agdeppa et al. (2017) | Cross-sectional | 6565 | 6-12/male and female | Two nonconsecutive-day 24-h dietary recalls, food | Phosporus | 1050 mg (113.3) |  |
| Angeles-Agdeppa et al. (2017) | Cross-sectional | 6565 | 6-12/male and female | Two nonconsecutive-day 24-h dietary recalls, food | Sodium | 500 mg (169.7) |  |
| Angeles-Agdeppa et al. (2017) | Cross-sectional | 6565 | 6-12/male and female | Two nonconsecutive-day 24-h dietary recalls, food | Magnesium | 155 mg (83.6) |  |
| Angeles-Agdeppa et al. (2017) | Cross-sectional | 6565 | 6-12/male and female | Two nonconsecutive-day 24-h dietary recalls, food | Potassium | 2000 mg (42.7) |  |
| Angeles-Agdeppa et al. (2017) | Cross-sectional | 6565 | 6-12/male and female | Two nonconsecutive-day 24-h dietary recalls, food | Selenium | 17.25 mg (456.8) |  |
| Torrico et al. (2021) | Secondary analysis of data derived | 3386 | 6-12/male and female | 24-hour food recall, food | VItamin C | 24.1 mg (53.6) |  |
| Torrico et al. (2021) | Secondary analysis of data derived | 3386 | 6-12/male and female | 24-hour food recall, food | Thiamin | 0.65 mg (81.3) |  |
| Torrico et al. (2021) | Secondary analysis of data derived | 3386 | 6-12/male and female | 24-hour food recall, food | Niacin | 12.4 mg (124) |  |
| Torrico et al. (2021) | Secondary analysis of data derived | 2800 | 13-18/male and female | 24-hour food recall, food | VItamin C | 28.3 mg (46.4) |  |
| Torrico et al. (2021) | Secondary analysis of data derived | 2800 | 13-18/male and female | 24-hour food recall, food | Thiamin | 0.81 mg (72.3) |  |
| Torrico et al. (2021) | Secondary analysis of data derived | 2800 | 13-18/male and female | 24-hour food recall, food | Niacin | 16.7 mg (111.3) |  |
| **Thailand** | | | | | | | |
| Rojroongwasinkul et al. (2013) | Cross-sectional | 511 | 6-12.9/boys and girls/urban | 24-hour recall questionnaire; food | Vitamin C | 31.5 mg (80.8) |  |
| Rojroongwasinkul et al. (2013) | Cross-sectional | 1059 | 6-12.9/boys and girls/rural | 24-hour recall questionnaire; food | Vitamin C | 23 mg (59) |  |
| **Vietnam** | | | | | | | |
| Nguyen et al. (2013) | Cross-sectional | 483 | 5-11.9/Girls/Urban | 24 h recall questionnaire, food | Vitamin C | 48 mg (111.63) |  |
| Nguyen et al. (2013) | Cross-sectional | 483 | 5-11.9/Girls/Urban | 24 h recall questionnaire, food | Vitamin C | 49 mg (113.16) |  |
| Nguyen et al. (2013) | Cross-sectional | 483 | 5-11.9/Girls/Urban | 24 h recall questionnaire, food | Vitamin C | 47 mg (109.3) |  |
| Nguyen et al. (2013) | Cross-sectional | 483 | 5-11.9/Girls/Urban | 24 h recall questionnaire, food | Vitamin C | 38 mg (87.76) |  |

**Fat-soluble vitamin intake**

**Vitamin E and Vitamin K**

Inadequacy of vitamin E intake was also observed among children in Indonesia, Malaysia, and the Philippines. Three studies from those countries reported that vitamin E intake ranged from 1.17 to 3.48 mcg/day (10.6%-34.8% EAR) (27,38,46). No data on this nutrient was found among Thai and Vietnamese children. Two studies exploring vitamin E intake in adolescents in Indonesia (49) and Malaysia (33) reported that vitamin E intake among this population was in various ranges, accounting for 12%-149.3% EAR. No data was found among adolescents in Thailand, Vietnam, and the Philippines. This review found no study of vitamin K intakes among school-age children and adolescents in Indonesia, Malaysia, Thailand, Vietnam, and Philippines.

**Water-soluble vitamin intake**

**Vitamin C**

Four studies from Indonesia explored vitamin C intake among children, and the results varied, ranging from 0.75 mg to 425.8 mg/day (35,39,40,49). One study (35) classified the population according to socioeconomic status (SES) and reported that vitamin C intake tended to be higher in children with high SES compared to low and medium SES. Another study revealed that vitamin C intake was considerably similar between urban and rural children (40). Four studies from Malaysia reported various vitamin C intakes among children ranging from 25 to 53,4 mg/day. A study in Indonesia and two studies in Malaysia exploring vitamin C among adolescents reported that vitamin C intake among this population varied from 20.2 to 93.4% RDA/RNI. No data was found among adolescents in Indonesia, Thailand, Vietnam, and the Philippines.

**Vitamin B3**

Three studies from Malaysia (27,30,36) and two studies from the Philippines (43,45) explored vitamin B3 (niacin) intake among children, and the results varied. While most of the studies from Malaysia reported that the intake level was below the recommended level, studies from the Philippines reported that intake exceeded the recommended level. Only one study about vitamin B3 intake from Malaysia was found, and it was reported to be nearly adequate (34). Data was not found among Indonesia, Thailand, and Vietnam. Intake studies from Malaysia and the Philippines (34,45) explored niacin intake, and the results varied from 97.3% EAR to 111.3% EAR. No study was found among Indonesian, Thai, and Vietnamese adolescents.

**Vitamin B6**

Three studies from Indonesia, Malaysia, and the Philippines investigated children’s vitamin B6 (pyridoxin) intake. The Philippines investigated vitamin B6 intake in children and reported that the intake was below recommended levels, ranging from 40% to 155.6% EAR. Data was not found among Thailand and Vietnam. As for adolescents, only one study from Indonesia (31) reported that the intake was below the recommended intake. Regarding vitamin B7 intake, studies have yet to be found to assess vitamin B7 (biotin) intake among children and adolescents across the five countries.

**Mineral and trace-elements intake**

**Phosphorus**

One study investigated other macrominerals among children (43) from the Philippines. The findings revealed varying levels of intake for different minerals. For phosphorus, the mean intake was reported as 1050 mg (113.3% RDA), and sodium intake was 500 mg (169.7% RDA), and both exceeded the recommended intake level. However, magnesium intake was relatively lower at 155 mg (83.6% of the RDA). Similarly, potassium intake was below the recommended level, with a mean intake of 2000 mg, corresponding to only 42.7% of the RDA.

**Sodium and Potassium**

Other macromineral intake among adolescents was also investigated only in one study from Malaysia. (33) which aimed to assess the dietary intake of sodium and potassium among adolescents aged 13-17, with separate analyses conducted for boys and girls. The findings revealed significant differences in the intake levels of these minerals between boys and girls. For sodium intake among boys, the mean intake was reported as 3371.7 mg (224.8% of RDA). The mean sodium intake among girls was lower at 2602.5 mg but significantly exceeded the RDA. In contrast, potassium intake was relatively lower for both boys and girls. The mean potassium intake among boys was 1416.1 mg (30.1% RDA). Similarly, the mean potassium intake among girls was 1170.8 mg (24.9% RDA).

**Copper**

Regarding copper intake among children, two studies each originated from Indonesia and Malaysia were found (37,49) and suggested significant disparities in copper intake levels between the Indonesian and Malaysian populations. Ekawanti (49) reported the varying levels of copper intake across different age and gender groups. Among 7-9-year-old males, the mean copper intake was 0.2 mcg (0.04% RDA). The mean intake for 10-12-year-old males increased to 3.87 mcg (0.6% of the RDA). Similarly, among females aged 7-9, the mean copper intake was 0.24 mcg, and for females aged 10-12, it was slightly higher at 0.37 mcg, both representing minimal percentages of the RDA. In contrast, study from Malaysia(37) found that among 7-8-year-olds, the mean copper intake was 230 mg (52.3% of the RNI) while among 9-12-year-olds, it was 230 mg (40.4% of the RNI). No studies on children's copper intake came from Vietnam, Thailand, and the Philippines.

Ekawanti et al (2021) (49) the only study assessed copper intake among adolescents across the five countries. The finding was copper intake among adolescents was 0.4 mcg (0.1% RDA). No study on adolescents’ copper intake from Malaysia, Thailand, Vietnam, and the Philippines.

**Mangan**

Only one study found assessed mangan intake among children and adolescents (49) and it was from Indonesia. This study reported varying levels of manganese intake across different age and gender groups. Among 7-9-year-old males, the mean manganese intake was reported as 0.96 mg (56.5% RDA). For 10-12-year-old males, the mean intake increased to 2.25 mg (118.4% RDA). Similarly, among females aged 7-9, the mean manganese intake was 1.1 mg (64.7% RDA), and for females aged 10-12, it was 1.87 mg (116.88% RDA). Among adolescent females aged 13-15, the mean manganese intake was notably higher at 3.2 mg (200% RDA).

**Selenium**

The study from the Philippines was the only one aimed to assess the dietary intake of selenium among children across the five countries(43). The findings revealed a mean selenium intake of 17.25 mg (456.8% of the RDA). No study was found on adolescents' selenium intake across the five countries, as well as iodine intake among children and adolescents.
